# Schistosomiasis as an urban disease: 13 years of studies in the neighborhoods of Salvador, BA, Brazil

**DOI:** 10.1371/journal.pntd.0013418

**Published:** 2026-10-23

**Authors:** Pedro Santos-Muccillo, Fernanda Mac-Allister Cedraz, Luciano K. Silva, Lúcio M. Barbosa, Vanessa T. Moretto, Vanessa S. Zanardi, Camila F. Chaves, João R. P. Cruz-Filho, Lee S. A. Andrade, Cláudio R. A. Santos, Eliana A. G. Reis, Cristiane W. Cardoso, Rogerio S. Teixeira, Fabiano M. Simões, Roberto J. M. Nascimento, Jackson M. L. Costa, Mitermayer G. Reis, Ronald E. Blanton

**Affiliations:** 1 Laboratory for Pathology and Molecular Biology, Gonçalo Moniz Institute, Oswaldo Cruz Foundation, Salvador, Bahia, Brazil; 2 Laboratory of Clinical Parasitology, Bahiana School of Medicine and Public Health, Salvador, Bahia, Brazil; 3 Health Surveillance Directorate, Municipal Health Secretariat of Salvador, Salvador, Bahia, Brazil; 4 Health Sciences Institute, Federal University of Bahia, Salvador, Bahia, Brazil; 5 Epidemiology of Microbial Diseases, Yale University, New Haven, Connecticut, United States of America; 6 Department of Tropical Medicine and Infectious Disease, Tulane Celia Scott Weatherhead School of Public Health and Tropical Medicine, New Orleans, Louisiana, United States of America; Zhejiang Wanli University, CHINA

## Abstract

**Background:**

Rapid urbanization in Brazil has shifted the epidemiological landscape of parasitic diseases, such as schistosomiasis and malaria. Although typically considered a rural infection, *Schistosoma mansoni* transmission persists in urban settings where sanitation and water access remain uneven. This study investigates the persistence and dynamics of urban schistosomiasis across neighborhoods of Salvador, Bahia, Brazil.

**Methods:**

Between 2011 and 2024 cross-sectional surveys were conducted to identify neighborhoods with the highest prevalence in the major metropolitan area of Salvador, Bahia. A total of 6,863 residents provided demographic information and Kato-Katz examination of at least one stool sample. We conducted demographic surveys and stool examination on 3,964 individuals who were re-examined on up to three occasions at intervals ranging from 1-5 years.

**Findings:**

The overall prevalence of initial examination was 7%. Regardless of treatment intervals, the community-wide prevalence declined by more than half and did not rebound. Apparent Incidence decreased to <0.5%/year following a second period of treatment. In nearly all communities, most infections occurred among individuals born in Salvador with no travel history outside the city.

**Interpretation:**

Overall, the study provides one of the first individual-level longitudinal assessments of schistosomiasis persistence in an urban Brazilian setting, combining repeated parasitological surveys with demographic and environmental data across multiple neighborhoods. These findings indicate that schistosomiasis transmission in Salvador is largely local rather than imported. The decline in prevalence coincides with observed community-level improvements in sanitation and reduced contact with surface water. f prevalence rates were applied across the population of Salvador, this city could be considered one of the state’s most important endemic areas.

## Author summary

Rapid urbanization and the lack of accompanying sanitation is urbanizing diseases previously considered rural. While sanitation in Salvador is generally good, *Schistosoma mansoni* infection was identified in all poor neighborhoods examined, and most evidence indicates this is a result of active transmission. Studies in urban environments pose difficulties not experienced or experienced to a lesser degree in most rural areas. Participation in research is lower, and violence is generally higher. Urban areas also offer opportunities for effective intervention due to the greater impact of infrastructure modifications and population density. Interventions tend to more easily affect a greater number of people. The prevalence of infection and the size of the city make urban infection an important focus for management. The Brazilian setting may provide a preview of schistosomiasis transmission as endemic areas become increasingly urbanized.

## 1. Introduction

Along with population growth and aging, one of humanity’s greatest demographic transitions over the past 120 years has been urbanization. According to the United Nations, the global population became predominantly urban in 2007 [1], and the process continues [2]. Although urbanization has been slower in parts of Africa and Asia, these regions now are also the most rapidly urbanizing [3]. Consequently, future studies of human disease should focus even more on urban areas, where most of humanity now resides. Much of this growth results from rural-to-urban migration rather than increased fecundity within cities [4]. As a result, diseases historically considered rural may emerge, and sometimes become established within urban environments [5–8].

In Brazil, rapid urbanization began after World War II, and the country became predominantly urban in the 1960s. Today, Brazil is more urbanized than the United States, with over 88% of its population residing in cities [9]. However, urbanization in Brazil has occurred amid substantial social and infrastructure disparities. Many urban neighborhoods, especially informal settlements and slums, face inadequate sanitation, unsafe water access, and limited healthcare coverage. Salvador, the capital of Bahia and Brazil's first colonial capital, exemplifies this pattern. Since 1980, the city’s growth has been explosive, with its population tripling in 20 years, reaching over 4 million residents [10]. Rapid urban expansion has led to the proliferation of densely populated neighborhoods where environmental conditions can sustain transmission of infections traditionally considered rural.

Schistosomiasis is considered the second most important parasitic disease of public health significance after malaria [11]. In 2019, schistosomiasis was responsible for 1.6 million Disability-Adjusted Life Years [11]. While most studies of the parasite have involved rural sites, *Schistosoma mansoni* was first identified in Brazil in the city of Salvador based on autopsies performed by Pirajá da Silva in 1908 [12]. More than a century later, transmission persists within the city’s limits. The identification of a high prevalence of *S. mansoni* among school-aged children in the Salvador neighborhood of São Bartholomeu [13] prompted further investigation of urban disease in São Bartolomeu [14] and other neighborhoods of the city.

To understand the persistence, distribution, and local dynamics of *S. mansoni* infection in Salvador, we conducted pilot studies in eight neighborhoods, longitudinal, cross-sectional studies in three neighborhoods, and longitudinal epidemiological and parasitological surveys with repeated treatment in three neighborhoods over the course of 13 years. Specifically, we sought to (i) determine the prevalence and temporal changes in infection following treatment, (ii) identify sociodemographic and environmental factors associated with *S. mansoni* infection; and (iii) distinguish locally acquired infections from potentially imported through migration or travel.

While previous studies in Brazil have examined schistosomiasis in urban or peri-urban areas through cross-sectional or ecological approaches, few have followed individuals over time to evaluate persistence and reinfection within city environments [5,15,16]. By integrating repeated individual-level parasitological data with demographic and environmental information, this study provides a longitudinal perspective over multiple neighborhoods on schistosomiasis persistence in an urban Brazilian setting.

## 2. Methods

### 2.1. Ethic statement

All subjects provided written informed consent or in the case of minors, written consent was obtained from their guardians. The study was approved by the Research Ethics Committee (CEP) of the Oswaldo Cruz Foundation, Bahia, the Brazilian Commission for Research Ethics (CONEP) under registry no. 42424915·9·0000·0040, the Institutional Review Boards of University Hospitals of Cleveland UHCMC IRB number: 01-14-32, and Tulane University administrative review 2019-1799.

### 2.2. Study design

Between 2011 and 2024, we conducted a multi-site prospective study of schistosomiasis in Salvador, Bahia, Brazil. Initial pilot studies were performed in eight neighborhoods with histories of snails shedding *S. mansoni* cercariae or recorded *S. mansoni* infections, reported from the Zoonotic Control Center or the Health Department of Salvador (See S1 Data). The pilots performed 1–2 weeks prior to the start of larger surveys served to select study areas predicted to have 5% infection prevalence. A sample calculation indicated that examination of at least 72 individuals would have sufficient power to detect 5% prevalence in a population of 2000 with 5% error and 95% confidence. Thus, a convenience sample of 100 residents was examined per site (Fig 1). Six neighborhoods were selected for demographic and Kato-Katz stool surveys. Due to infection prevalence less than 5%, three neighborhoods (Dique do Cabrito, Bate Facho, and Lagoa da Paixão) were surveyed once, providing cross-sectional data. Three neighborhoods with baseline prevalence greater than 5% (São Bartolomeu, Saramandaia, and Pirajá) were surveyed multiple times, at intervals of 1–5 years (Fig 2). Longitudinal studies allowed assessment of infection persistence, apparent incidence, and reinfection. After each survey, all individuals found to have S. mansoni eggs in their stool were treated, and treatment was repeated for those with positive stool examinations at subsequent visits. Individuals not previously examined were allowed to enroll during follow-up surveys. For clarity, study sites are abbreviated by two letters followed by the survey year (e.g., SB2011 for São Bartolomeu 2011). Data from São Bartolomeu (2011, 2015) and Pirajá (2019) were published previously [14,17,18]. This study adds longitudinal studies from these neighborhoods as well as baseline and longitudinal studies from four additional neighborhoods.

**Fig 1:**
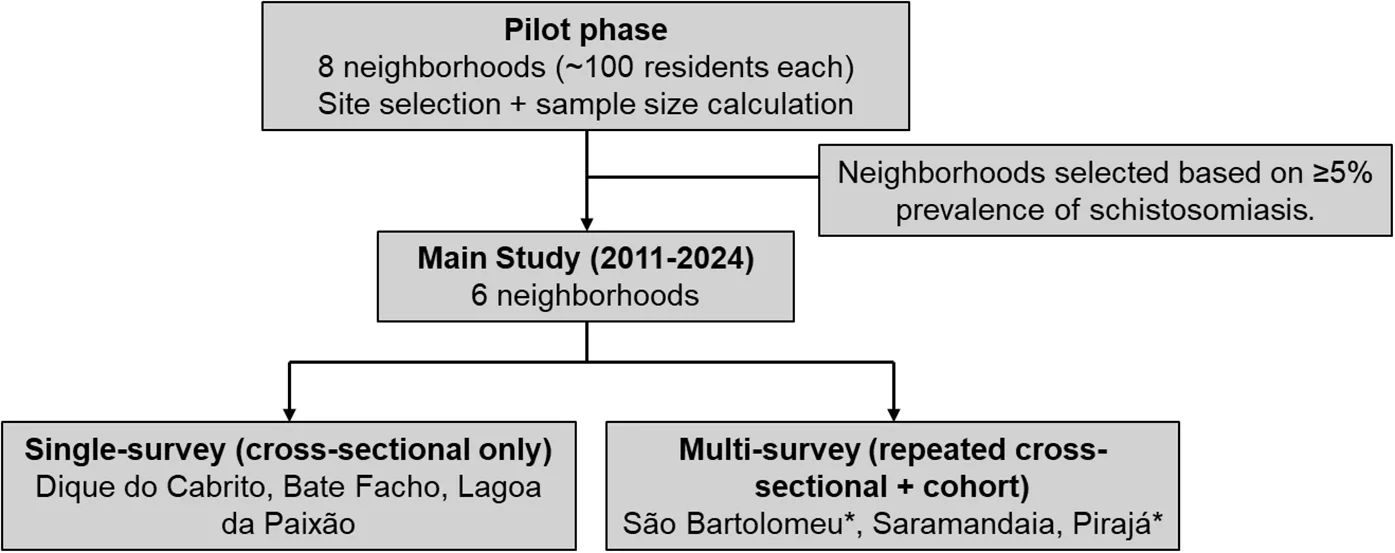
Study design and site selection process for urban schistosomiasis surveys in Salvador, Brazil (2011–2024). Sites with >5% prevalences of S. mansoni were selected for cross-sectional or longitudinal studies. The main study consisted of a demographic survey, stool survey X 3 and treatment and repeat examination and treatment as needed. *São Bartolomeu (2011, 2015) and Pirajá (2019) were published previously.

**Fig 2:**
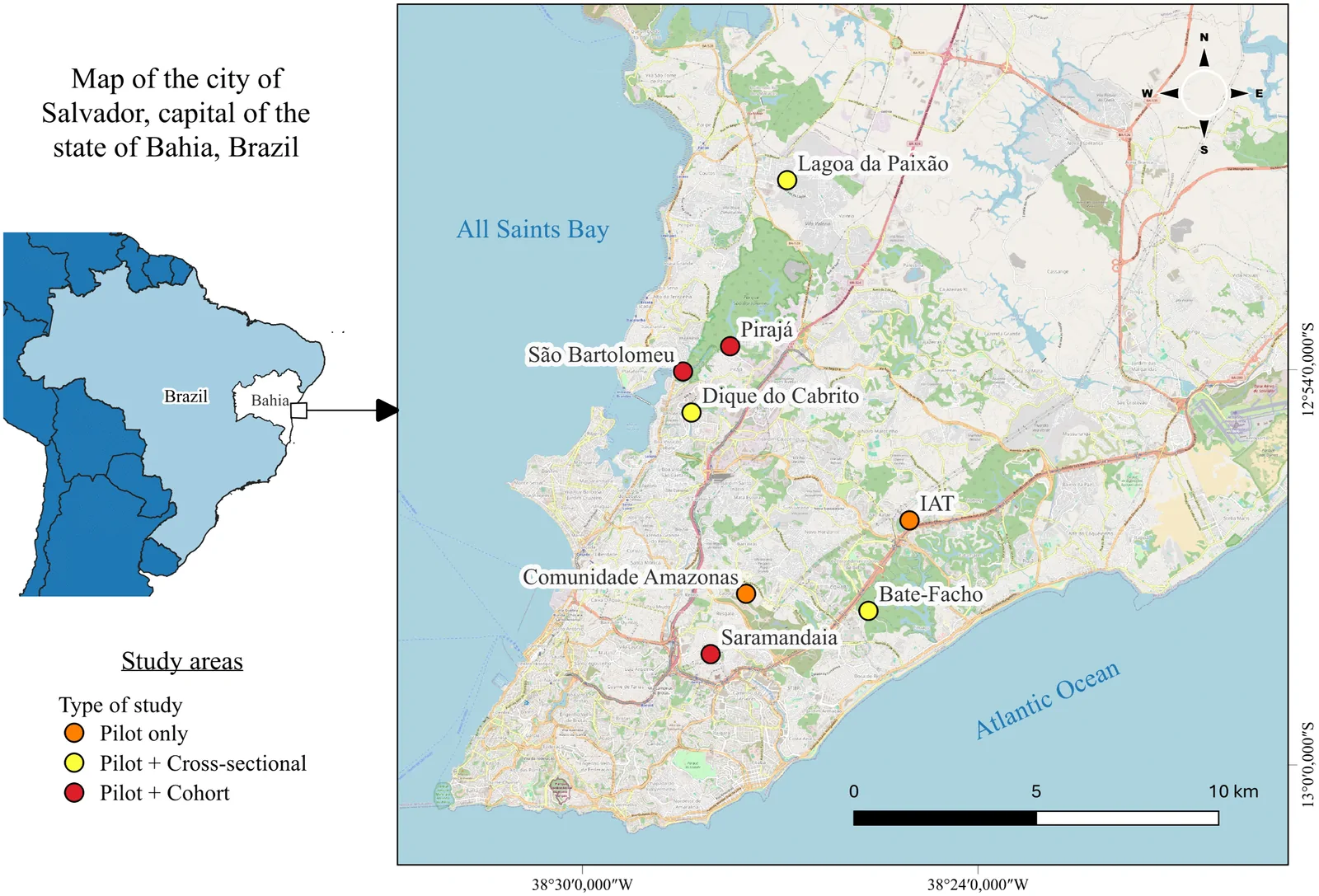
Salvador, Bahia, Brazil map of all study sites. Pilot studies were conducted in all areas. Baseline complete cross-sectional surveys only were conducted in three areas and repeat longitudinal surveys in three neighborhoods (See S1 Table). Study sites were georeferenced over OpenStreetMap database. Map use statement: Map created by the authors using QGIS software. Base map and data from OpenStreetMap and OpenStreetMap Foundation are available under the Open Database License (ODbL). Cartography is licensed under the Creative Commons Attribution-ShareAlike 2.0 license (CC BY-SA 2.0). Geographic outlines and base data are available at: https://www.openstreetmap.org. Attribution: OpenStreetMap contributors.

### 2.3. Study sites

Urban neighborhoods of Salvador are densely populated and often lack discrete boundaries. To focus on areas with potential for active schistosomiasis transmission, neighborhoods were selected based on their proximity to permanent water bodies (lakes, streams, and rivers) where snails of the species *Biomphalaria glabrata* had been observed by the municipal Zoonotic Control Center. The observed usage of these waters by the population for work, fishing and recreation was an additional criterion. Water at all sites had previously been recorded to have heavy human fecal contamination using an assay for human-associated strains of *Bacteroides* sp [19]. or *Lachnospira* sp [20]. Large agricultural plots were adjacent to the communities of São Bartolomeu and Saramandaia. These produced primarily watercress and other greens. Finally, these neighborhoods were also among the most socioeconomically disadvantaged areas of Salvador, characterized by informal settlements, limited sanitation coverage, and high levels of violence.

### 2.4. Recruitment

Prior to initiating the research, we contacted local public health clinics and community leaders at each site to communicate the project’s objectives and seek guidance on community access. Educational and community engagement activities were performed to sensitize each community and describe our research protocol. Whenever possible, we collaborated directly with the local health agents and always trained and hired personnel from the community during the collection of data and samples. This participatory approach strengthened local trust and facilitated sustained follow-up in subsequent survey periods.

### 2.5. Demographic survey

For baseline and follow-up studies, all households closest to surface water bodies within a buffer zone of 300–500 m were georeferenced using printed maps and subsequently recorded in QGIS version 3.40.13·3·4·8 [21]. Each household was then visited to identify the head of household and the total number of household members prior to the start of each survey. All household members were invited to enroll, and all participants answered a questionnaire covering demographic, socioeconomic, and epidemiological characteristics including number of surface water sites contacted. Data from the interviews for all household members were directly entered into Android-based devices (Android version 9·1, Samsung Galaxy Tab A 8·0) using a REDCap tool (version 13·1·26-2024) [22] The REDCap platform was hosted at the Oswaldo Cruz Foundation, Bahia.

Since there was a four-year interval between surveys for São Bartolomeu, socioeconomic conditions were compared between 2011 and 2015 based on the Criteria for Economic Classification Brazil of the Brazilian Association of Research Companies, São Paulo (Criteria for Economic Classification Brazil 2013. http://www.abep.org/Servicos/Download.aspx?id=03).

### 2.6. Stool survey and treatment

For the diagnosis of *S. mansoni*, whole stool samples were collected from each participant on three different days. Each participant was provided with a barcoded container labeled with their name, ID number, and house number. Sample identifiers were verified in the laboratory before processing. A single Kato-Katz smear (41.7 mg) was prepared from each sample and examined microscopically for *S. mansoni* eggs [23]. Participants with at least one *S mansoni* egg-positive stool sample were considered infected and received a single oral dose of praziquantel (60 mg/kg for children 4–15 years old and 50 mg/kg for adults) [24]. Treatment was directly observed in nearly all cases; only a few participants received medication for home administration. Because the Kato-Katz technique also detects other helminths, all participants who tested positive for any soil-transmitted helminths were treated with a single dose of albendazole 400 mg. For participants positive for *S. mansoni*, stool examinations were reexamined three times, 30 days after treatment, and were re-treated if found to be positive.Participants who were negative 30 days post-treatment but later tested positive in subsequent surveys were classified as reinfected. During treatment visits and household follow-ups, participants received education on schistosomiasis prevention and the risk associated with contact with surface water in their communities.

### 2.7. Data analysis

Raw data were exported to EpiInfo 7·2·2·6 [25] and/or R/R Studio [26] for statistical univariate and multivariate analyses. Sample size calculations were performed to detect both differences in infection prevalence and odds ratios, assuming an 80% power, 5% significance level, and 95% confidence interval. Categorical comparisons were performed by a chi-square test using Yates’ correction or a Fisher Exact test when appropriate. Multivariate logistic regression analysis was performed with all variables with significant association from the univariate analysis. A two-sided *p* value <0·05 was considered significant for all analyses. The 95% confidence interval (95%CI) with continuity correction was calculated using the VassarStats website [27]. Measures of association were estimated using prevalence ratios (PR) or odds ratios (ORs).

For participants with longitudinal follow-up, apparent incidence was defined as the proportion of individuals who were negative at baseline and positive at the next survey period, divided by the number of years between examinations. Because continuous person-time data were unavailable, these estimates represent apparent rather than true incidence. Migrants were defined as all participants who were not born in Salvador or had not spent 100% of their lifetime as residents.

Spatial analysis was performed with QGIS version 3·34·8 [21]. Geographical buffers of 500 m were created around principal water contact sites within each neighborhood. Subsequently, buffers with variable widths were generated using the “Multiple ring buffers” or “Variable width buffer” tool in QGIS to facilitate the analysis of infection risk associated with proximity to specific water contact points.

## 3. Results

### 3.1. Participation and population characteristics

The 300-500m buffer zones were designed to include 2,000–2,500 residents. Of these, 11,093 (~89%) individuals were contacted, provided informed consent, and were interviewed. A total of 6,863 participants (62%) from six different neighborhoods in Salvador provided at least one stool sample between 2011 and 2023 (Table 1). Fig 3 summarizes recruitment, participation, and follow-up across study sites.

**Table 1:** Baseline cross-sectional results in Salvador neighborhoods.

Characteristics	SB2011 (n = 1,050)	DC2017 (n = 1,253)	SA2018 (n = 1,786)	PI2019 (n = 1,128)	LP2022 (n = 1,035)	BF2023 (n = 611)	Total (N = 6,863)
	% or mean ± SD	% or mean ± SD	% or mean ± SD	% or mean ± SD	% or mean ± SD	% or mean ± SD	% or mean ± SD
Male sex	45.8%	41.7%	43.1%	41.1%	40.0%	42.7%	42.4%
Age	29.4 ± 17.4	32.8 ± 20.1	31.2 ± 19.0	34.0 ± 20.7	33.1 ± 20.4	38.3 ± 20.7	32.6 ± 19.7
Born in Salvador	78.7%	73.6%	59.1%	82.7%	79.7%	65.4%	72.3%
%Life in SSA	88.1 ± 19.9	94.0 ± 16.0	88.0 ± 23,0	88.0 ± 23.0	30.5 ± 19.6	85.0 ± 23.0	89.7 ± 20.7
Lived outside SSA	34.4%	17.8%	29.7%	8.5%	15.1%	24.7%	22.2%
Any travel outside last year	33.1%	39.9%	37.7%	19.9%	8.1%	43.9%	30.7%
Piped water	99.9%	96.3%	97.1%	99.6%	90.2%	99.1%	96.9%
Indoor Toilet	95.9%	98.4%	99.7%	99.0%	98.0%	100.0%	98.6%
Adequate sanitation^a^	67.8%	95.0%	95.2%	99.0%	100.0%	85.7%	92.0%
*Sm* prevalence	23.5%	2.5%	5.7%	6.0%	2.4%	0.8%	7.0%
*Sm* intensity of infection^b^	187.0 ± 384.3	43.1 ± 51.4	63.6 ± 107.1	84.6 ± 164.9	31.8 ± 23.7	28.8 ± 16.1	127.0 ± 294.3
*Sm* intensity of infection^c^	56.1	27.3	29.3	34.8	23.5	24.6	41.3

Neighborhoods: SB - São Bartolomeu; DC - Dique do Cabrito; SA - Saramandaia; PI - Pirajá; LP - Lagoa da Paixão; BF - Bate-Facho; *Sm* - *S· mansoni*. SSA – Salvador, Bahia, Brazil.

^a^Municipal sewer connection or septic tank, ^b^ Arithmetic mean ± standard deviation (SD) ^c^ Geometric mean (eggs per gram of feces).

**Fig 3:**
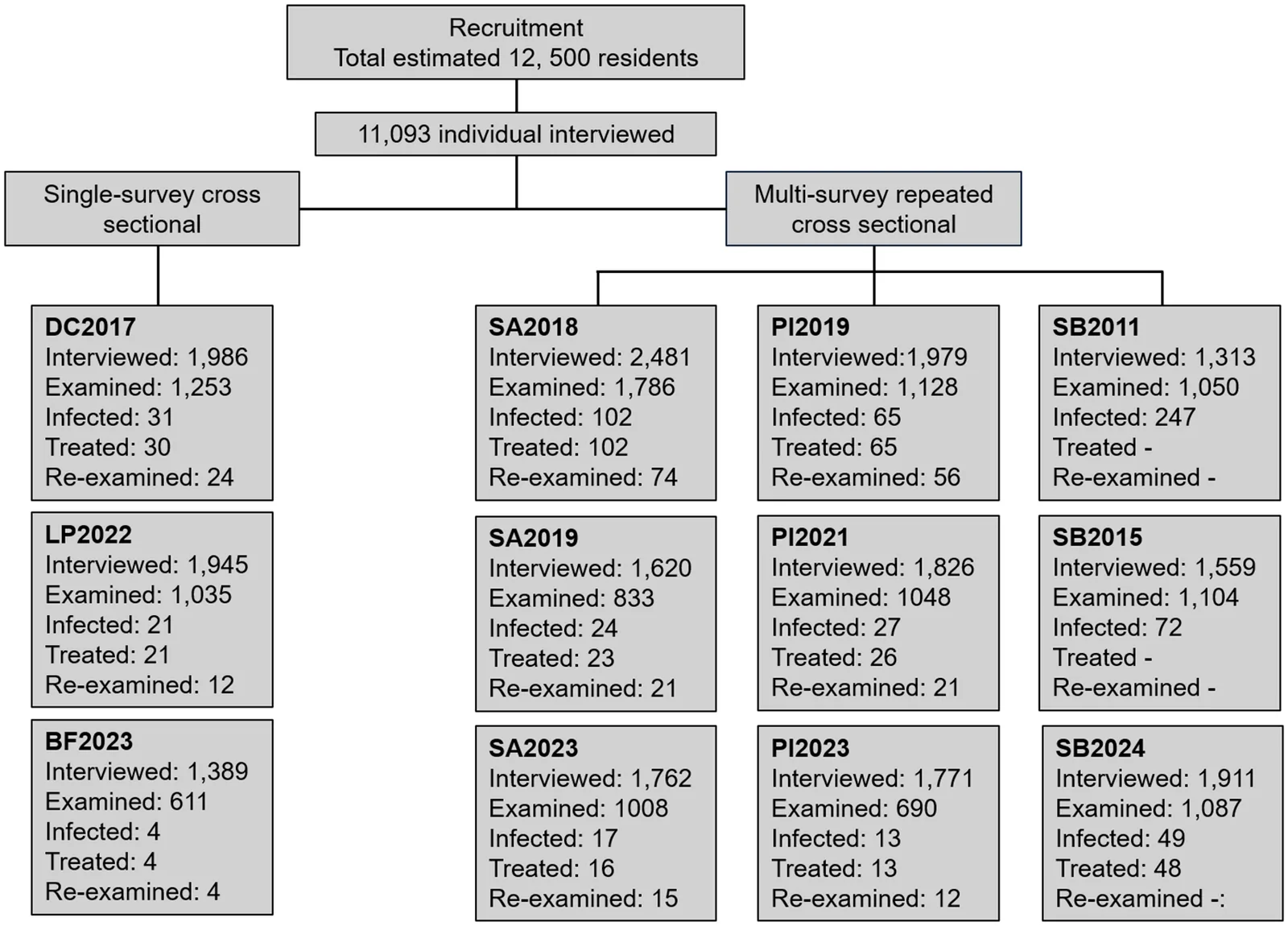
Recruitment, participation, treatment, and follow-up flow diagram for all study sites (2011–2024). Numbers represent participants at each stage (interviewed, examined, infected, treated, and re-examined). Non-participation at follow-up was primarily due to relocation, refusal, illness, or inability to contact individuals during follow-up.

Among all participants, 42.4% were males, average age 32.6, SD ± 19.7. Most (72.3%) were born in Salvador, and non-native residents spent 89.7%, SD ± 20.7% of their lifetime in the city. Over 90% of households reported a monthly income below R$3,276.76 (approximately US$650). Any travel outside of Salvador was reported by 31% of the participants. Piped water was present in 96.9% of households, and 92.0% reported connection to the municipal sewer system or a septic tank, although some residents who reported otherwise were not aware they had no municipal sewer connection. In São Bartolomeu, where four years and an urban renewal project interrupted a follow-up survey, there was no change in personal wealth, although adequate sewer coverage increased from 69% to 92% [17]. Most characteristics were similar among the sites (sex, age, born in Salvador, etc.), with some important differences such as time (Table 1).

### 3.2. Cross-sectional prevalence and factors associated with *S. mansoni* infection

The overall prevalence of *S. mansoni* infection was 7.0% across all neighborhoods (Table 1). The higher prevalence in São Bartolomeu in 2011 was similar to that of some endemic areas from the interior of the State [28]. If São Bartolomeu 2011 (SB2011) were excluded, overall baseline prevalence dropped to 3.9% (95%CI 3.4 – 4.3). Bate Facho 2023 (BF2023) presented the lowest prevalence (0.8%), followed by Lagoa da Paixao 2022 (LP2022) (2.4%), and Dique do Cabrito 2017 (DC2017) (2.5%), while São Bartolomeu 2011 SB2011 (23.5%), Saramandaia 2018 (SA2018) (5.7%), and Piraja 2019 (PI2019) (6.0%) had the highest initial values. SB2011 also presented the lowest adequate sanitation coverage among all sites (67.8%), while other sites reported in excess of 90%.

Schistosomiasis prevalence was higher among young adults between the ages of 21 and 30 years in all neighborhoods (Tables 2 and 3). The highest prevalence in SB2011, SA2018, and PI2019 was between ages 31 and 40 years old, and in LP2022 between ages 51 and 60 years old (Fig 4). Due to the small number of cases in BF2023, it was not possible to identify the most prevalent age group. The distribution of schistosomiasis prevalence by age groups was similar when stratified by sex, but it was always higher among men at all ages (Fig 5).

**Table 2:** Factors associated with the prevalence of *S. mansoni* in Salvador at baseline, 2011 to 2023.

Characteristics	N^a^	n	Prev.	Crude OR (95%CI)	p-value	Adjusted OR (95% CI)	p-value
Total	6,863	478	7.0%	–		–	
Study^b^							
SB2011	1,050	247	23.5%	37.28 (15.28-90.93)	<0.001	44.64 (14.17-140.62)	<0.001
PI2019	1,128	68	6.0%	7.78 (3.12-19.39)	<0.001	10.74 (3.35-34.43)	<0.001
SA2018	1,786	102	5.7%	7.34 (2.98-18.10)	<0.001	10.09 (3.18-32.00)	<0.001
DC2017	1,253	31	2.5%	3.07 (1.19-7.95)	0.02	4.48 (1.36-14.77)	0.01
LP2022	1,035	25	2.4%	3.00 (1.14-7.88)	0.03	4.07 (1.21-13.65)	0.02
BF2023	611	5	0.8%	1		1	
Sex							
Male	2,913	264	9.1%	1.74 (1.44-2.10)	<0.001	1.81 (1.48-2.23)	<0.001
Female	3,950	214	5.4%	1		1	
Age, years							
≥ 21	4,579	340	7.4%	1.25 (1.02-1.53)	0.03	1.51 (1.21-1.89)	<0.001
< 21	2,284	138	6.0%	1		1	
Born in Salvador							
Yes	4,944	347	7.0%	1.06 (0.86-1.31)	ns	–	–
No	1,893	126	6.7%	1			
Lived outside SSA							
Yes	1,364	108	7.9%	1.19 (0.95-1.49)	ns	–	–
No	5,364	362	6.7%	1			
Any travel outside last year							
Yes	2,076	139	6.7%	0.96 (0.78-1.17)	ns	–	–
No	4,681	327	7.0%	1			
Piped water							
No	205	10	4.9%	0.70 (0.37-1.32)	ns	–	–
Yes	6,270	430	6.9%	1			
Indoor Toilet							
No	56	0	0.0%	0.01 (Undef)	ns	–	–
Yes	5,991	440	7.3%	1			
Adequated sanitation							
No	516	102	19.8%	4.13 (3.24-5.27)	<0.001	1.75 (1.32-2.33)	<0.001
Yes	5,939	334	5.6%	1		1	

^a^Total varies according to the availability of data. Percentages are estimates over this total. ^b^ Neighborhoods: SB - São Bartolomeu; DC - Dique do Cabrito; SA - Saramandaia; PI - Pirajá; LP - Lagoa da Paixão; BF - Bate-Facho; *Sm* - *S· mansoni*. SSA – Salvador, Bahia, Brazil.

Undef - undefined, ns - not significant.

**Table 3:** Longitudinal cohort studies in Salvador.

Characteristics	SB2015 (n = 1,104)	SB2024 (n = 1,087)	SA2019 (n = 833)	SA2023 (n = 1,008)	PI2021 (n = 1,048)	PI2023 (n = 690)
	% or mean ± SD	% or mean ± SD	% or mean ± SD	% or mean ± SD	% or mean ± SD	% or mean ± SD
Male sex	41·8%	40·55%	42·7%	41·8%	41·6%	36·5%
Age	33·7 ± 198	38·2 ± 20·9	33·0 ± 19·7	36·1 ± 19·8	36·0 ± 21·1	39·3 ± 22·0
Born in Salvador	74·1%	91·4%	60·1%	61·4%	83·3%	82·9%
%Life in SSA	87·0 ± 21·0	96·0 ± 13·0	89·0 ± 21·0	89·0 ± 22·0	89·0 ± 21·0	91·0 ± 20·0
Lived outside SSA	25%	7·8%	29·7%	27·2%	9·7%	10·3%
Any travel outside last year	39·7	9·2%	36·1%	34·9%	16·8%	15·8%
Piped water	98·9	94·6%	98·9%	96·3%	98·4%	92·0%
Indoor Toilet	100·0	100·0%	99·6%	98·3%	97·9%	99·3%
Adequated sanitation	100·0	99·7%	93·9%	95·7%	99·4%	99·4%
*Sm* mansoni global prevalence	6·2%	4·5%	2·9%	1·7%	2·6%	1·9%
*Sm* intensity of infection^1^	162·7 ± 294·5	77·4 ± 160·5	74·2 ± 189·2	28·7 ± 33·7	90·4 ± 122·1	97·5 ± 107·0
*Sm* intensity of infection^2^	73·4	34·3	24·4	18·0	40·1	54·6
*Sm* incidence/year (%)	8·9%	0·3%	2·2%	0·4%	1·0%	0·4%
*Sm* intensity^a^	190·1 ± 376·5	122·7 ± 258·0	34·9 ± 47·0	21·3 ± 27·1	89·4 ± 117·7	71·3 ± 107·3
*Sm* intensity^b^	77·8	44·4	19·4	14·5	43·0	36·5
*Sm* prevalence new participants	4·5%	5·1%	6·4%	2·5%	1·8%	5·6%
*Sm* intensity^a^	129·1 ± 139·2	57·4 ± 88·8	139·6 ± 302·2	46·4 ± 44·3	96·0 ± 165·5	120·0 ± 109·6
*Sm* intensity^b^	68·5	30·6	35·8	30·1	27·0	77·0

Neighborhoods: SA = Saramandaia; PI = Pirajá; SB = São Bartolomeu; *Sm* = *S· mansoni*.

* Total varies according to the availability of data. Percentages are estimates over this total.

^a^Arithmetic mean ± standard deviation (SD) ^b^ Geometric mean (eggs per gram of feces.

**Fig 4:**
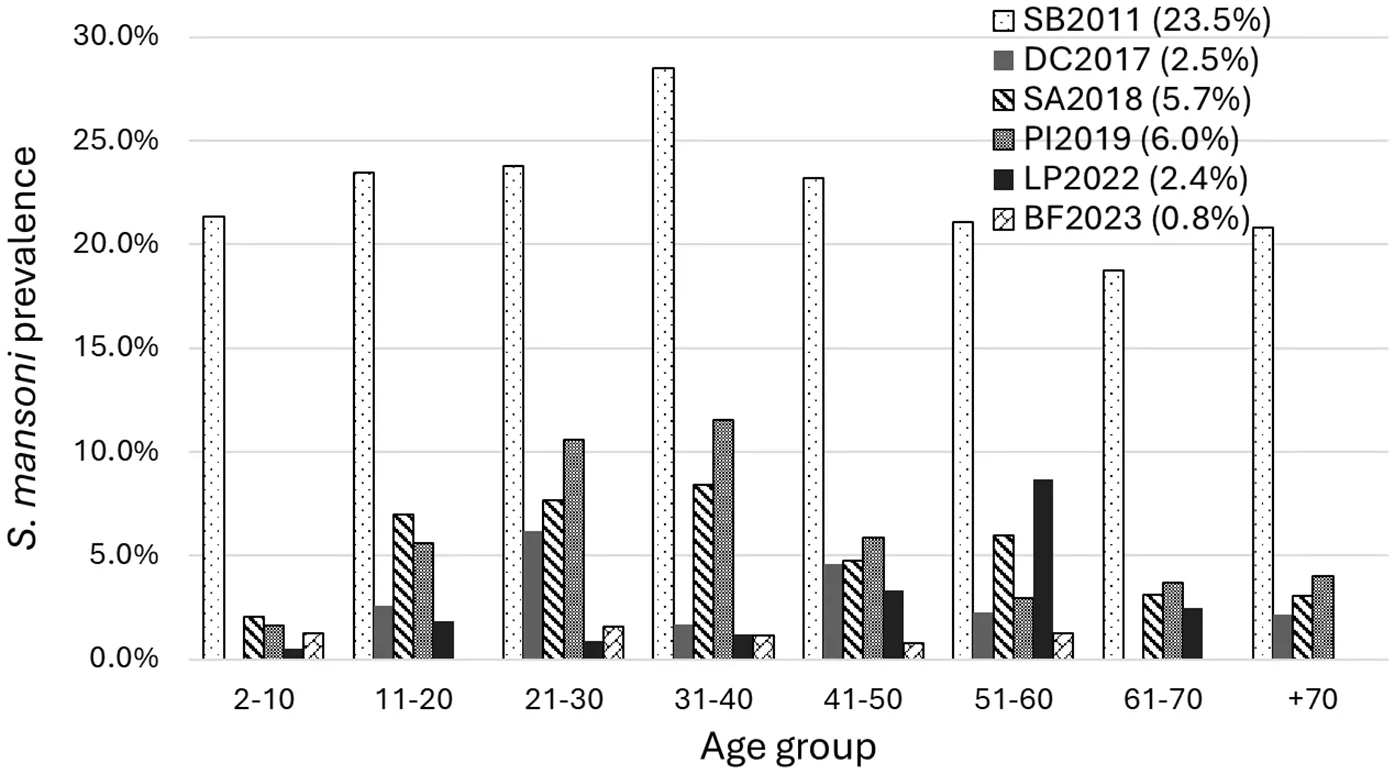
Initial prevalence of *S. mansoni* (SM) infections by age group and by neighborhood. SB2011 (São Bartolomeu 2011), DC2017 (Dique do Cabrito 2017), SA2018 (Saramandaia 2018), PI2019 (Pirajá 2019), LP2022 (Lagoa da Paixão 2022), BF2023 (Bate Facho 2023).

**Fig 5:**
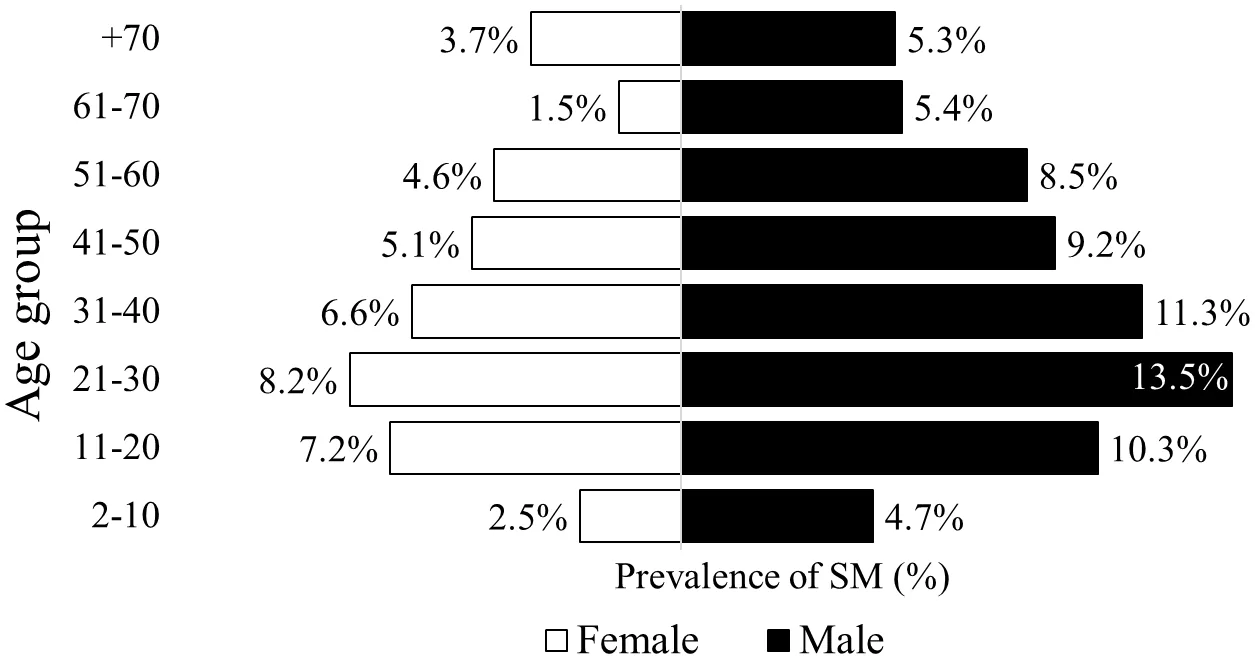
Age and *S. mansoni* (SM) infection by sex. Labels show the percent among all those infected and grouped by age group and sex, e.g., 4.7% of all infections were boys 4-10 years old. SM – *S. mansoni.* Female (n = 3950), Male (n = 2913).

By logistic regression, infection was more frequent among males (9.1%) than females (5.4%), corresponding to an adjusted OR = 1.8 (95% CI: 1.5–2.2, p < 0.001). Mean prevalence was higher among participants aged ≥ 21 years than those younger (aOR = 1.5 [95% CI 1.2-1.9], p < 0.001), and those living without adequate sanitation (aOR = 1.75 [95% CI 1.3-2.3], p < 0.001) (Table 2). Immigrant status, travel outside Salvador, and access to piped water or indoor toilets were not associated with infection. We also observed an association between the prevalence of schistosomiasis and proximity to surface waters in most communities (Fig 6). The only exception was SB2011, where the prevalence of *S. mansoni* was independent of the distance of the household from a water body.

**Fig 6:**
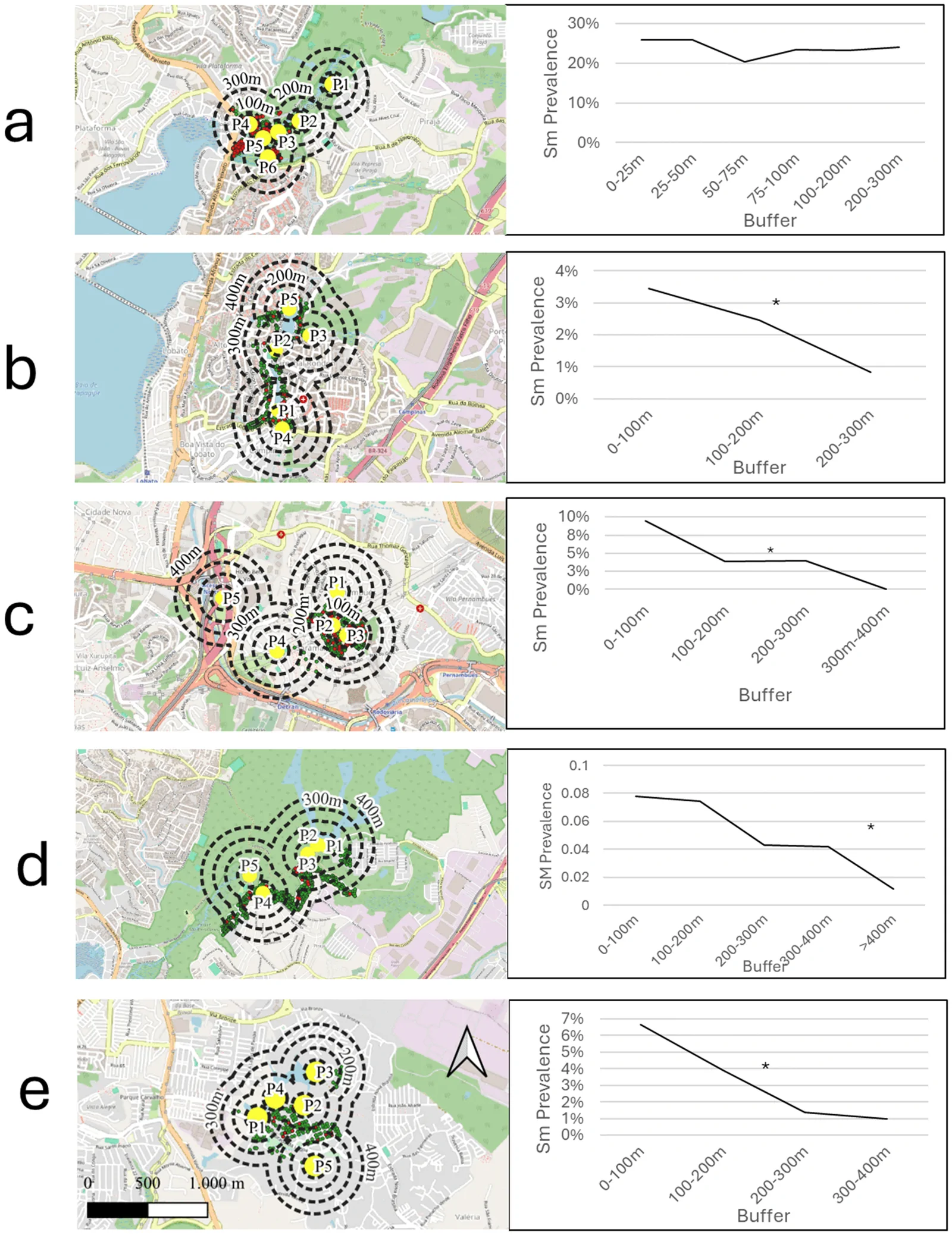
Correlation between *S. mansoni* infection prevalence and distance from residence Salvador, 2011 - 2023. **a.** São Bartolomeu 2011 (SB2011); **b.** Dique do Cabrito 2017 (DC2017); **c.** Saramandaia 2018 (SA1018); **d.** Pirajá 2019 (PI2019); **e.** Lagoa da Paixão 2023 (LP2023). Buffer zones were created at 500 m intervals from water contact points and the prevalence of *S. mansoni* infection calculated within each zone. *SM* mean prev. – *S. mansoni* mean prevalence. Brown points - residences with at least one *Sm*+ case. Green points - residences with no *Sm* case. * Chi-Square for trend (p < 0.05). Note: BF2023 had very few cases and this analysis could not be performed. Map features were georeferenced over OpenStreetMap database. Map use statement: Map created by the authors using QGIS software. Base map and data from OpenStreetMap and OpenStreetMap Foundation are available under the Open Database License (ODbL). Cartography is licensed under the Creative Commons Attribution-ShareAlike 2.0 license (CC BY-SA 2.0). Geographic outlines and base data are available at: https://www.openstreetmap.org. Attribution: OpenStreetMap contributors.

### 3.3. Demographic patterns and migration

While most participants were long-term residents, those in Saramandaia presented the lowest percentage of native born compared with the other communities (Tables 1 and 3). Most immigrants migrated from endemic regions of Bahia, as well as from other endemic states. The older agricultural workers from SB 2011 and SA2018 stated that they were born in Sergipe, a state bordering Bahia, and came to Salvador to grow urban crops as their main source of income (Reis, M.G. personal communication). Those workers, as well as their sons born in Salvador, had the highest prevalence andintensity among all participants. For eight agricultural workers at SB2011 the prevalence was 100% with an average of 1,006 ± 2,093 eggs per gram of feces, while for nine agricultural workers at SA2018 the prevalence was 33% with an average of 57 ± 63 eggs per gram of feces. The initial prevalence and intensity for these workers in 2018 may have been reduced by case finding and treatment by the municipal health department the year before. Meanwhile, despite differences in the type of vegetables grown between the two agricultural sites and environmental changes, all workers at São Bartolomeu were infected at each survey period, whereas at Saramandaia, transmission was apparently interrupted since no infections were observed in this group following their two periods of treatment.

The migrants to LP2022 had the shortest residence in the neighborhood (30·5% of lifetime), although most residents were born in the city. Thus, this area was composed mostly of new residents (70%) who came to Lagoa da Paixão from other neighborhoods of Salvador just after the construction of government-sponsored housing in 2013 and 2015.

### 3.4. Longitudinal follow-up

Three communities were re-surveyed two to three times over intervals of 1–5 years, allowing assessment of infection persistence and apparent incidence (Table 3): São Bartolomeu (2011, 2015, 2024), Saramandaia (2018, 2019, 2023), Pirajá (2019, 2021,2023). Prevalence decreased substantially after the first treatment period in each community with an incidence of 1.3 to 2.9/year at the second treatment period and 0.5% by the third treatment period. In São Bartolomeu, prevalence fell from 6.2% in 2015 to 4.5% in 2024. In Saramandaia, prevalence dropped from 5.7% in 2018 to 1.7% in 2023, and in Pirajá, from 6.0% in 2019 to 1.9% in 2023. Apparent incidence observed in those egg negative on one examination and found to be positive on a subsequent examination ranged from 0.4 to 8.9% per year. The 2–4 individuals persistently positive at 30 days post treatment and who were retreated were considered subsequently egg negative. On average, 25% of all infections in previously examined individuals were reinfections, and 75% were in those previously negative.

Among previously infected individuals who were cured, only a small proportion became re-infected. Most reinfections occurred among households located near surface water bodies. Despite differences in infrastructure and environmental modifications, transmission persisted at low levels in São Bartolomeu and Pirajá but appeared interrupted in Saramandaia, where no new infections were detected after two periods of treatment.

### 3.5. Environmental and structural changes

Comparing each community over time, improvements in economic and infrastructure, and important environmental changes were observed. Three out of the six neighborhoods received structural interventions from the Brazilian federal program for affordable housing Minha Casa Minha Vida (MCMV): São Bartolomeu (SB2013 and SB2018), Pirajá (PI2014), and Lagoa da Paixão (LP2013 and LP2015). These projects included drainage, paved streets, and sewage system expansion. Other neighborhoods experienced incremental changes, such as septic installation and garbage collection improvement. Water contact points in Saramandaia were also paved. Overall, socioeconomic conditions showed gradual improvement modestly all sites over time, although the pace and extent of infrastructural development varied among neighborhoods.

## 4. Discussion

The process of urbanization in Salvador has been marked by a proliferation of communities in areas initially devoid of basic infrastructure, including clean water, sanitation, and waste management. In recent decades, municipal and federal initiatives, however, are taking steps to mitigate these conditions with measurable improvements in sanitation and socio-economic conditions. Consistent with these structural improvements, we observed reductions in the prevalence, apparent incidence, and intensity of *S. mansoni* infection over time, both among residents who participated in prior intervention periods and among newly enrolled individuals [17] These findings suggest that improved sanitation and socioeconomic conditions, together with repeated case detection and treatment, contributed to the decline in infection levels. The higher prevalence among new participants likely reflects untreated infections among residents who were missed in earlier periods of intervention.

Prevalence was higher among male participants in all age groups, a trend consistent with previous studies in Brazil [29,30]. Schistosomiasis is often linked with school-aged children [31]. By contrast, in this study, across all neighborhoods, urban schistosomiasis, was most prevalent in the age group 21–30 years, with certain neighborhoods such as São Bartolomeu in 2011 peaking in an even older age groups (31–40 years). In the urban context, studies that only enroll school age children may underestimate schistosomiasis prevalence. Given the male prevalence and older age group, schistosomiasis in Salvador is most likely related to occupational exposure and behavior, with adult men more frequently exposed to contaminated water. While three stool samples were examined per participant to improve diagnostic sensitivity, low-intensity infection may still have been missed. This limitation may have led to underestimation of the prevalence and apparent incidence, particularly in later surveys when infection intensity was lower.

Although there has been significant migration since the 1980s, our findings indicate that local transmission appears to contribute more to the presence and persistence of infection than migration. Infection prevalence and intensity were similar between immigrants and those born in Salvador. There were similar genetic profiles for *S. msnaoni* from both groups seen in our previous studies [14,18] Local transmission is further supported by the inverse association of infection prevalence and household distance from surface water an unlikely pattern for infection due to migration in these areas [30,32].

Sanitation was significantly associated with the prevalence of schistosomiasis in Salvador. Although having inadequate sanitation is not just a risk for an individual household, here this variable may reflect economic status and overall ability to avoid risk from contamination. Across the city, sewer coverage went from 26% in 1995 to 69% in 2004 [33]. In neighborhoods such as São Bartolomeu in 2011, where only 69% of households had adequate sewage disposal, the prevalence of *S. mansoni* was significantly higher than all other sites. By 2015, reported coverage was near 90% at all sites, including São Bartolomeu, and no prevalence was above 6%. Essentially every home in Salvador, no matter how humble, has a flush toilet, but if the home employs straight pipe discharge to surface water, this will more directly inject fecal material into snail habitats than even open defecation. In contrast, neighborhoods with better rates of sewer connection, such as the Lagoa da Paixão (LP2022) and those benefiting from the federal Minha Casa Minha Vida housing program (e.g., São Bartolomeu in 2015), had lower prevalences. The urban environment itself may promote snail habitat suitability due to reduced numbers of snail competitors and predators as well as small scale agricultural land-use and sewage outflows [34].

Our longitudinal analyses further demonstrate that schistosomiasis can persist at low levels even after repeated periods of treatment and environmental improvement. This persistence highlights the resilience of urban foci where human–environment contact remains frequent and sanitation infrastructure incomplete. Control strategies must therefore extend beyond chemotherapy to address the environmental and behavioral dimensions of exposure.

We have studied schistosomiasis using similar approaches in both rural [28,35,36] and urban areas. One of the main challenges for controlling schistosomiasis in urban areas lies in part with urban dynamics. Urban populations are more internally mobile and have more intense commercial activity. People can be more difficult to reach due to formal or informal work. Also, in rural areas, populations may view a research team as a diversion, while in urban areas it is more likely to be viewed as a distraction. Finally, urban studies must contend with urban violence, at times an additional impediment to participation. Participation in the urban setting is usually half of what we observed in rural areas. Our findings reflect the conditions of economic vulnerability and poor sanitation that characterize rapidly forming urban communities in Brazil, underscoring the potential for schistosomiasis to persist in cities if environmental and socio-economic conditions are not addressed. There are, however, advantages urban areas present over rural areas for implementation of control measures. Given urban density, any control measure will affect many more individuals. Also, since the area is more compact, structural changes to entire neighborhoods can be contemplated. Hopefully, not all changes involve cementing over rivers and streams, but emphasize preventing feces going in rather than restricting access to them.

Despite the overall decline in prevalence, schistosomiasis remains an important public health concern in Salvador. Estimates of prevalence of S. mansoni infection for Salvador are at 2%. Even if only 1% of the city population are infected, this represents 30,000 infected residents. This is a number that exceeds the total populations of 70% of municipalities in the state of Bahia. Continued surveillance, targeted control, and integration of urban sanitation planning with health programs will be critical to eliminate urban transmission and prevent resurgence.

There are several limitations to be considered in this review. The neighborhoods included here were selected for their proximity to significant collections of surface water and only a portion of what administratively were defined as the neighborhood was included; therefore, it may not represent the city as a whole. The surveys were conducted over more than a decade when significant changes were occurring in the conditions of the neighborhoods. The neighborhoods were not studied over the same time intervals. Finally, the participation of zoonotic sources was not taken into account. In some areas of the Americas, certain rodent species may participate in the transmission of *S. mansoni*. In Brazil the principle species is the water rat, *Nectomys squamipes* [37,38] that does not inhabit urban areas. A rodent survey of the city revealed primarily *Rattus novegicus* and rare *Rattus rattus* [39]. The former is not a permissive host.

Despite these limitations, the findings demonstrate that urban schistosomiasis can persist even as sanitation and living conditions improve. Sustained surveillance, integration of environmental and epidemiological data, and continued investment in urban infrastructure are essential to consolidate recent gains and move toward elimination of transmission in Salvador and other rapidly urbanizing areas.

## 5. Conclusions

This study provides a longitudinal perspective on the persistence and dynamics of *S. mansoni* infection in an urban Brazilian setting. Over more than a decade of repeated surveys and treatment cycles, prevalence and apparent incidence declined substantially, consistent with improvements in sanitation and socioeconomic conditions. Infection remained more frequent among adult males and those living in areas with inadequate sewage disposal, underscoring the role of environmental exposure in maintaining transmission. Similar infection rates among immigrants and native-born residents, together with spatial associations between prevalence and proximity to surface waters, indicate that transmission in Salvador is largely sustained locally rather than driven by imported infections.

## Supporting information

S1 Table Site selection and study design for urban schistosomiasis surveys in Salvador, Brazil (2011–2024). (DOCX)

S1 Data Neighborhood Descriptions Salvador, Bahia. (DOCX)
