# Supplementary material for: Schistosomiasis as an urban disease: 13 years of studies in the neighborhoods of Salvador, BA, Brazil
Source: PLoS Negl Trop Dis. 2026 Oct 23;20(10):e0013418. doi: 10.1371/journal.pntd.0013418 (PMC13734582; doi:10.1371/journal.pntd.0013418)
Supplement: Supplementary file 1 [file pntd.0013418.s003.pdf]

PLOS Neglected Tropical Diseases

Schistosomiasis as an urban disease: 13 years of studies in the neighborhoods of Salvador, Bahia, Brazil

--Manuscript Draft--

Manuscript Number:	PNTD-D-25-01291
Full Title:	Schistosomiasis as an urban disease: 13 years of studies in the neighborhoods of Salvador, Bahia, Brazil
Short Title:	Urban schistosomiasis in Brazil
Article Type:	Research Article
Keywords:	Schistosoma mansoni; urbanization; migration; sanitation; water-borne disease; poverty; ecology; agriculture
Abstract:	Background Concern over urbanization of parasitic diseases like schistosomiasis and malaria parallels the growth in urbanization of the human population. Rapid urbanization has meant most Brazilians now live in cities, and while schistosomiasis is generally considered a rural problem, there is transmission and persistence in urban areas.<br>Methods Cross sectional pilot studies were used to identify neighborhoods with highest prevalence in the major metropolitan area of Salvador, Bahia. A total of 6,863 residents provided demographic information and at least one stool sample. Some 3,964 were tested, treated, and re-examined to confirm egg-negativity three times at intervals ranging from 1-5 years.<br>Findings The global prevalence on initial examination was 7%. Regardless of treatment intervals, the community-wide prevalence declined by more than half and never recovered. Incidence decreased to <0.5%/year following a second round of treatment. In all communities except one, most of those infected had been born in Salvador and most did not travel outside.<br>Interpretation: Most infections were locally acquired and little due to recent migration. Recently published population genetic studies of the parasite were consistent with local transmission. Much improvement could be associated with structural changes in the communities that improved sanitation and prevented contact with surface waters. If prevalence rates were applied across the population of Salvador, this city could be considered one of the state's most important endemic areas.
Additional Information:	
**Question**	**Response**
**Government Employee**<br><br>Are you or any of the contributing authors an employee of the United States government?<br><br>Manuscripts authored by one or more US Government employees are not copyrighted, but are licensed under a CC0 Public Domain Dedication, which allows unlimited distribution and reuse of the article for any lawful purpose. This is a legal requirement for US Government employees.	No - No authors are employees of the U.S. government.

Powered by Editorial Manager® and ProduXion Manager® from Aries Systems Corporation

This will be typeset if the manuscript is accepted for publication.	
**Financial Disclosure**<br><br>Enter a financial disclosure statement that describes the sources of funding for the work included in this submission. Review the submission guidelines for detailed requirements. View published research articles from *PLOS Neglected Tropical Diseases* for specific examples.<br><br>This statement is required for submission and **will appear in the published article** if the submission is accepted. Please make sure it is accurate.<br><br>**Funded studies**<br>Enter a statement with the following details:<br>• Initials of the authors who received each award<br>• Grant numbers awarded to each author<br>• The full name of each funder<br>• URL of each funder website<br>• Did the sponsors or funders play any role in the study design, data collection and analysis, decision to publish, or preparation of the manuscript?<br><br>Did you receive funding for this work?	Yes
Please add funding details.<br>as follow-up to "**Financial Disclosure**<br><br>Enter a financial disclosure statement that describes the sources of funding for the work included in this submission. Review the submission guidelines for detailed requirements. View published research articles from *PLOS Neglected Tropical Diseases* for specific examples.<br><br>This statement is required for submission and **will appear in the published article** if the submission is accepted. Please make sure it is accurate.	This work was supported by Grant NIAID R01AI121330 from the National Institutes of Health (NIH-USA); Fundação de Amparo à Pesquisa do Estado da Bahia (FAPESB)/PRONEX PNX0001/2015; and in part the Coordenação de Aperfeiçoamento de Pessoal de Nível Superior - Brasil (CAPES) - Finance Code 001.

**Funded studies**<br>Enter a statement with the following details:<br>• Initials of the authors who received each award<br>• Grant numbers awarded to each author<br>• The full name of each funder<br>• URL of each funder website<br>• Did the sponsors or funders play any role in the study design, data collection and analysis, decision to publish, or preparation of the manuscript?<br><br>Did you receive funding for this work?"	
Please select the country of your main research funder (please select carefully as in some cases this is used in fee calculation).<br>as follow-up to "**Financial Disclosure**<br><br>Enter a financial disclosure statement that describes the sources of funding for the work included in this submission. Review the submission guidelines for detailed requirements. View published research articles from *PLOS Neglected Tropical Diseases* for specific examples.<br><br>This statement is required for submission and **will appear in the published article** if the submission is accepted. Please make sure it is accurate.<br><br>**Funded studies**<br>Enter a statement with the following details:<br>• Initials of the authors who received each award<br>• Grant numbers awarded to each author<br>• The full name of each funder<br>• URL of each funder website<br>• Did the sponsors or funders play any role in the study design, data collection and analysis, decision to publish, or preparation of the manuscript?<br><br>Did you receive funding for this work?"	UNITED STATES - US
**Competing Interests**	The authors have no competing interests.

On behalf of all authors, disclose any competing interests that could be perceived to bias this work.<br><br>This statement will be typeset if the manuscript is accepted for publication.<br><br>Review the instructions link below and PLOS NTDs' competing interests policy to determine what information must be disclosed at submission.	
**Data Availability**<br><br>Provide a **Data Availability Statement** in the box below. This statement should detail where the data used in this submission can be accessed. This statement will be typeset if the manuscript is accepted for publication.<br><br>Before publication, authors are required to make all data underlying their findings fully available, without restriction. Review our PLOS Data Policy page for detailed information on this policy. Instructions for writing your Data Availability statement can be accessed via the Instructions link below.	Data sharing statement<br>De-identified individual participant data collected during the study is freely available through the Digital Object Identifier (DOI 10.25833/1acj-zj58).

Powered by Editorial Manager® and ProduXion Manager® from Aries Systems Corporation

Manuscript
Click here to access/download;Manuscript;Urban Schisto PLoS NTD.docx

Schistosomiasis as an urban disease: 13 years of studies in the neighborhoods of Salvador, Bahia, Brazil

Pedro Santos-Muccillo, MS[a,*], Fernanda Mac-Allister Cedraz, MS[a,*] Luciano K. Silva, PhD[a], Lúcio M. Barbosa PhD[b], Vanessa T. Moretto, PhD[a], Vanessa S. Zanardi, MS[a], Camila F. Chaves, MS[a], João R. P. Cruz-Filho, MS[a], Lee S. A. Andrade, M.S.[a], Cláudio R. A. Santos[a], Eliana A. G. Reis, PhD[a], Cristiane W. Cardoso, PhD[c], Rogerio S. Teixeira[c], Fabiano M. Simões[c,†], Roberto J. M. Nascimento[e], Jackson M. L. Costa, MD[a], Mitermayer G. Reis, MD[a,d,e], Ronald E. Blanton, MD[f,§].

Author affiliations:
[a] Gonçalo Moniz Institute, Oswaldo Cruz Foundation, Salvador, Bahia, Brazil;
[b] Bahiana School of Medicine and Public Health, Salvador, Bahia, Brazil;
[c] Municipal Health Department, Salvador, Bahia, Brazil;
[d] Yale University, New Haven, Connecticut, USA;
[e] Federal University of Bahia, Salvador, Bahia, Brazil;
[f] Tulane Celia Scott Weatherhead School of Public Health and Tropical Medicine, New Orleans, Louisiana, USA.

* These authors contributed equally to this work. † *In memorium*. § Corresponding Author: Ronald Blanton, Department of Tropical Medicine, Tulane Celia Scott Weathrhead School of Public Health and Tropical Medicine, 1440 Canal Street, New Orleans, LA 70112, 504-988-7067, rblanton1@tulane.edu

ABSTRACT

Background Concern over urbanization of parasitic diseases like schistosomiasis and malaria parallels the growth in urbanization of the human population. Rapid urbanization has meant most Brazilians now live in cities, and while schistosomiasis is generally considered a rural problem, there is transmission and persistence in urban areas.

Methods Cross sectional pilot studies were used to identify neighborhoods with highest prevalence in the major metropolitan area of Salvador, Bahia. A total of 6,863 residents provided demographic information and at least one stool sample. Some 3,964 were tested, treated, and re-examined to confirm egg-negativity three times at intervals ranging from 1-5 years.

Findings The global prevalence on initial examination was 7%. Regardless of treatment intervals, the community-wide prevalence declined by more than half and never recovered. Incidence decreased to <0.5%/year following a second round of treatment. In all communities except one, most of those infected had been born in Salvador and most did not travel outside.

Interpretation: Most infections were locally acquired and little due to recent migration. Recently published population genetic studies of the parasite were consistent with local transmission. Much improvement could be associated with structural changes in the communities that improved sanitation and prevented contact with surface waters. If prevalence rates were applied across the population of Salvador, this city could be considered one of the state's most important endemic areas.

Key words: *Schistosoma mansoni*, urbanization, migration, sanitation, water-borne disease; poverty; ecology; agriculture

1. **Introduction**

Along with population growth and aging, humanity's great demographic story of the last 120 years has been one of urbanization. According to the WHO, the world human population became predominantly urban in 2007, and the process continues. While the process has been slower in Africa and parts of Asia, these regions are also the most rapidly urbanizing [1]. This means that future studies of human disease will focus even more on urban areas since this is where most humans will reside. The increase now primarily results from rural to urban migration rather than increased fecundity in urban dwellers. In some cases, this will mean diseases that had been thought of as predominantly rural will be carried along and possibly established in cities [2].

In Brazil, rapid urbanization began after World War II, and the country became predominantly urban in the 1960's. Today, Brazil is more urbanized than the United States, with over 88% of its population residing in cities [3]. Salvador, Bahia, was the first capital of Brazil and has long been an important regional commercial center. Since 1980, the city's growth has been explosive, with its population tripling in 20 years, so that today the Salvador metropolitan area has more than 4 million residents [4]. This rapid urban expansion has led to the formation of informal settlements and slums, where residents often face precarious living conditions, inadequate sanitation, and limited access to healthcare.

Schistosomiasis is a parasitic infection considered to be the most widespread and serious after malaria. It is also considered to be a mostly rural concern, even though in 1908 Pirajá da Silva first differentiated the species *Schistosoma mansoni* from *S. haematobium* based on autopsies performed in the city of Salvador [5]. The identification of a high prevalence of *S. mansoni* among school-aged children in the Salvador neighborhood of São Bartholomeu [6] prompted our investigation of urban disease in this [7] and other neighborhoods of the city. This article reviews our experience with urban schistosomiasis based on pilot studies in eight neighborhoods and longitudinal epidemiologic and parasitologic surveys with repeated treatment in three neighborhoods over the course of 13 years.

2. Methods

2.1. Study design

Between 2011 and 2024, we conducted pilot studies in eight neighborhoods of the city of Salvador, Bahia, Brazil by stool examinations of a convenience sample of 100 residents for *S. mansoni* infection (Figure 1). Between 2017 and 2024, six of those neighborhoods including São Bartolomeu were selected for in-depth demographic and stool surveys (Figure 1). Three communities were surveyed only once and three were re-surveyed three times separated by intervals of 1 to 5 years with repeated treatment of positive cases for schistosomiasis. For convenience the study sites may be abbreviated with two letters followed by the year surveyed (São Bartolomeu – SB2011, SB2015, and SB 2024; Dique do Cabrito – DC2017; Saramandaia – SA 2018, SA2019, and SA2023; Pirajá – PI2019, PI 2021, and PI2023; Bate Facho – BF2023; Lagoa da Paixão – LP2023). Data from São Bartolomeu 2011 and 2015 were published previously [8] as well as from Pirajá 2019 [9].

2.2. Study sites

Urban neighborhoods of Salvador are densely populated and often without discrete boundaries. To focus on active schistosomiasis transmission, neighborhoods were selected based on their proximity to permanent water bodies (lakes, streams, and rivers) where snails of the species *Biomphalaria glabrata* had been observed by the municipal Zoonotic Control Center. The degree of contact of the population with these waters was an additional criterion. Water at all sites showed evidence of heavy human fecal contamination using an assay for human-associated strains of *Bacteroides* sp. [10] or *Lachnospira* sp. [11]. Finally, these areas are among the poorest and most violent of the city.

2.3. Recruitment

Prior to initiating the research, we contacted local public health clinics and community leaders at each site to communicate the project's objectives and seek guidance on community access. We performed educational and community engagement activities to sensitize each community and describe our research protocol. Whenever

possible, we collaborated directly with the local health agents and always trained and hired personnel from the community during the collection of data and samples.

2.4. Demographic Survey

For demographic surveys, households closest to surface water bodies within a buffer of 300 to 500 m were georeferenced. These households were then visited to identify the head of household and total number of household members prior to the start of each survey. Participants answered a questionnaire on demographic, household, and other epidemiological characteristics. All interviews were directly entered into Android-based devices (Android version 9·1, Samsung Galaxy Tab A 8·0) using a REDCap tool (version 13·1·26-2024) [12] The REDCap platform was hosted at the Oswaldo Cruz Foundation, Bahia.

2.5. Stool Survey and treatment

For diagnosis of *S. mansoni* and geohelminths, whole stool samples were collected on three different days. Each participant was provided with a barcoded container labeled with their name, ID number, and house number. Stool sample IDs were checked in the lab prior to preparation of a single slide and examination by the Kato-Katz method. Participants with at least one *S mansoni* egg-positive stool sample were given a one-time dose of praziquantel (60 mg/kg for children 4–15 years old and 50 mg/kg for adults). Almost all infected participants underwent observed treatment, and few received medication for home treatment. For soil-transmitted helminthiases, a single dose of albendazole 400 mg was provided. For all *S. mansoni* infected participants, the stool examinations were repeated X3 after 30 days. When presenting for treatment and at home visits, participants were educated about the risks of contact with surface water in their neighborhoods.

2.6. Data analysis

Raw data were exported to EpiInfo 7·2·2·6 [13] and/or R/R Studio [14] for statistical univariate and multivariate analyses. Categorical comparisons were performed by a chi-square test using Yates' correction or a Fisher Exact test when appropriate. Continuous

variables were evaluated with the Kruskal-Wallis test. Multivariate logistic regression analysis was performed with all variables with significant association from the univariate analysis. A two-sided *p* value <0·05 was considered significant for all analyses. The 95% confidence interval (95%CI) with continuity correction was calculated using the VassarStats website. [15] Measures of association were estimated using prevalence ratios (PR) or odds ratios (ORs).

Spatial analysis was performed with QGIS version 3·34·8. [16] Geographical buffers of 500 m were created around principal water contact sites within each neighborhood. Subsequently, buffers with variable widths were generated using the "Multi-ring buffers" or "Variable width buffer" tool in QGIS to facilitate the analysis of infection risk associated with proximity to specific water contact points.

3. Results

The total estimated number of residents in the buffer areas of all neighborhoods was estimated to be 13,040. Of this total, 10,383 individuals were contacted, provided informed consent, and were interviewed. Ultimately, a total of 6,863 participants from six different neighborhoods in Salvador participated in initial studies from 2011 to 2023 by providing at least one stool sample (Table 1). Of all participants 42·4% were male, average age 32·6, SD ± 19·7, and 72·3% were born in Salvador. On average non-native residents spent 89·7%, SD ± 20·7% of their lifetime in Salvador. More than 90% of families had an estimated monthly household income less than R$3,276·76 (approximately US$650). Some travel outside of Salvador was reported by 31%. Piped water was present in 96·9% of households, and 92·0% reported connection to the municipal sewer system or a septic tank, although some residents who reported otherwise were unaware that they did not have a municipal sewer connection.

Most characteristics were similar among the sites with some important differences. The global prevalence of schistosome infection was 7·0%. The initial survey of São Bartolomeu in 2011 occurred at an earlier date than other areas. Its higher prevalence is like that of some traditional endemic areas from the interior of the State.[17] São Bartolomeu

in 2011 also presented the lowest adequate sanitation coverage among all sites (67·8%). When SB2011 was excluded, overall prevalence was 3·9% (95%CI 3·4 – 4·3). BF2023 presented the lowest prevalence (0·8%), followed by LP2022 (2·4%), and DC2017 (2·5%), while SB2011 (23·5%), SA2018 (5·7%), and PI2019 (6·0%) had the highest initial values.

More than 70% of residents in all but one neighborhood were born in Salvador. SA2018, however, presented the lowest percentage of native born (59·1%) compared with the other communities. Most immigrants migrated from endemic regions within Bahia, particularly from inland areas, as well as from other endemic states. The older agricultural workers from SB 2011 and SA2018 stated that they were born in Sergipe, a state bordering Bahia, and came to Salvador to grow urban crops as their main source of income (Reis, M.G. unpublished). Those workers, as well as their sons born in Salvador, had the highest prevalence and parasitic loads among all participants. For garden works at SB2011 the prevalence was 100% and 1,006 ± 2,093 eggs per gram of feces for the eight workers, while for garden workers at SA2018 the prevalence was 33% with an average of 57 ± 63 eggs per gram of feces). The initial prevalence and intensity for SA2018 workers may have been influenced by case finding and treatment by the municipal health department the year before. Meanwhile, despite differences in the type of vegetables grown and environmental changes, all workers at São Bartolomeu were infected at each survey period, whereas at Saramandaia transmission was apparently interrupted since no infections were observed following their two rounds of treatment.

The migrants to LP2022 had the shortest residence in the neighborhood (30·5% of lifetime), although most residents were born in the city. Most residents arrived from other neighborhoods of Salvador. Thus, this area was composed mostly of new residents (70%) who came to Lagoa da Paixão just after the construction of government sponsored housing in 2013 and 2015.

Schistosomiasis prevalence was higher among young adults between age 21 and 30 years in all neighborhoods (Tables 2 and 3). The highest prevalence in SB2011, SA2018, and PI2019 was between ages 31 and 40 years old and in LP2022 between age 51 and 60 years old (Figure 2). Due to the small number of cases in BF2023, it was not

possible to identify the most prevalent age group. The distribution of schistosomiasis prevalence by age groups was similar when stratified by sex, but it was always higher among men in all ages (Figure 3).

In regression analysis immigrant status was not associated with infection, and the prevalence and intensity of infection were no different between immigrants and the native-born. We did observe an association between the prevalence of schistosomiasis and proximity to surface waters in most communities (Figure 4). The only exception was SB2011 where the prevalence of *S. mansoni* was independent of the distance of the household from water.

Three communities were re-surveyed longitudinally (Table 3): São Bartolomeu (2011, 2015, 2024), Saramandaia (2018, 2019, 2023), Pirajá (2019, 2021,2023). Comparing each community over time, economic characteristics improved in some areas, however, there were also important structural and environmental changes. Three out of the six neighborhoods received structural interventions from the Brazilian federal program for affordable housing program Minha Casa Minha Vida (MCMV). These were São Bartolomeu (SB2013 and SB2018), Pirajá (PI2014), and Lagoa da Paixão (LP2013 and LP2015).

4. Discussion

The process of urbanization in Salvador has been marked by a proliferation of communities in areas initially devoid of basic infrastructure, including clean water, sanitation, and waste management. The municipal and Federal governments, however, are taking steps to mitigate these conditions with promising results. With measurable improvements in sanitation and socio-economic conditions, the prevalence, incidence, and intensity of infection decreased whether residents were in the treated group or not. [18] There was also an independent contribution from case finding and treatment, since "new" participants in the study tended to have higher prevalence than those who participated previously. Most of these new participants were residents that skipped the initial round of intervention.

Prevalence was higher among male participants in all age groups, a trend consistent with previous studies in Brazil. [19–21] Schistosomiasis is often linked with school age children. [22] By contrast, in this study, across all neighborhoods, urban schistosomiasis, was most prevalent in the age group 21–30 years, with certain neighborhoods such as São Bartolomeu in 2011 peaking in an even older age groups (31–40 years). In the urban context, studies that only enroll school age children may underestimate schistosomiasis prevalence. Given the male prevalence and older age group, schistosomiasis in Salvador is most likely related to occupational exposure and behavior, with adult men more frequently exposed to contaminated water.

Although there has been significant migration since the 1980s, local transmission appears to contribute more to the presence and persistence of infection than migration. Infection prevalence and intensity were similar between immigrants and those born in Salvador, and there were similar genetic profiles for parasites from both groups seen in our previous studies [7,9] Local transmission is further supported by the inverse association of infection prevalence and household distance from surface water an unlikely pattern for infection due to migration in these areas. [21,23] Infection prevalence was related to the proximity of homes near surface water bodies, except in the case of São Bartolomeu in 2011. We believe that transmission there was sufficiently high at this time that no correlation could be observed at the narrow geographic scale studied. The relationship of distance to a water body and risk may be dependent on degree of sanitation as well as scale since sanitation was also poorest in São Bartolomeu in 2011.

Sanitation played an important role in determining the prevalence of schistosomiasis in Salvador through local transmission. In neighborhoods such as São Bartolomeu in 2011, where only 67·8% of households had inadequate sewage disposal, the prevalence of *S. mansoni* was significantly higher. Essentially every home In Salvador, no matter how humble, has a flush toilet, and if the home employs straight pipe discharge to surface water, this will more directly inject fecal material into snail habitats than even open defecation. In contrast, neighborhoods with better rates of sewer connection, such as the Lagoa da Paixão (LP2022) and those benefiting from the federal

Minha Casa Minha Vida housing program (e.g., São Bartolomeu in 2015), had lower prevalences. The urban environment itself may promote snail habitat suitability [24]

We have studied schistosomiasis with similar approaches in both rural and urban areas. A major challenge for schistosomiasis control in urban areas is in part the areas' very vitality. Urban populations are more mobile internally. People can be more difficult to reach due to formal or informal work. Also, in rural areas, populations may view a research team as a diversion, while in urban areas it is more likely to be viewed as a distraction. Finally, urban studies must contend with urban violence, at times an additional impediment to participation. Participation in the urban setting is usually half of what we observed in rural areas. Our findings reflect the conditions of economic vulnerability and poor sanitation that characterize rapidly forming urban communities in Brazil, underscoring the potential for schistosomiasis to persist in cities if environmental and socio-economic conditions are not addressed. There are, however, advantages urban areas present over rural areas for implementation of control measures. Given urban density, any control measure will affect a much larger number of individuals. Also, since the area is more compact, structural changes to entire neighborhoods can be contemplated. Hopefully, not all changes involve cementing over rivers and streams, but emphasize preventing feces going in rather than people staying out. Estimates of prevalence of schistosome infection for Salvador are at 2%. If even 1% of the population were infected, the infected population of Salvador would be greater than the total population of 70% of the counties in the state of Bahia.

5. Limitations

The neighborhoods included here were selected for their proximity to significant collections of surface water and only a portion of what administratively were defined as the neighborhood was included. The surveys were collected over more than a decade when significant changes were occurring in the conditions of the neighborhoods. The neighborhoods were not studied over the same time intervals.

CRediT authorship contribution statement

Pedro Santos-Muccillo: Investigation, Formal analysis, Methodology Writing – original draft, Writing – review & editing; Fernanda M. Cedraz: Investigation, Formal analysis, Writing – review & editing; Luciano K. Silva: Methodology, Data Curation, Formal analysis, Writing – review & editing, Visualization; Lúcio M. Barbosa: Investigation, Methodology, Writing – review & editing; Vanessa T. Moretto: Investigation; Vanessa S. Zanardi: Investigation; Camila F. Chaves: Investigation; João R. P. Cruz-Filho: Investigation; Lee S. A. Andrade: Investigation, Cláudio R. A., Santos: Investigation; Eliana A. G. Reis: Investigation; Cristiane W. Cardoso: Resources, Project administration; Rogerio S. Teixeira: Project administration; Fabiano M. Simões: Investigation[,†], Roberto J. M. Nascimento: Investigation; Jackson M. L. Costa: Investigation, Supervision; Mitermayer G. Reis: Conceptualization, Project administration, Resources, Supervision; Ronald E. Blanton: Conceptualization, Funding acquisition, Methodology, Writing – review & editing.

[,†]deceased

Acknowledgments

We thank the health posts and health agents of each neighborhood that helped us to accomplish this work. To our field and laboratory staff, working hard to include as many participants as possible and helping us obtain research data.

Data sharing statement

De-identified individual participant data collected during the study is freely available through the Digital Object Identifier (DOI 10.25833/1acj-zj58).

Author Statements

The study was approved by the Research Ethics Committee (CEP) of the Oswaldo Cruz Foundation, Bahia, the Brazilian Commission for Research Ethics (CONEP) under registry no. 42424915·9·0000·0040, the Institutional Review Boards of University Hospitals of Cleveland UHCMC IRB number: 01-14-32, and Tulane University administrative review 2019-1799.

The authors have no competing interests. This work was supported by Grant NIAID R01AI121330 from the National Institutes of Health (NIH-USA); Fundação de Amparo à Pesquisa do Estado da Bahia (FAPESB)/PRONEX PNX0001/2015; and in part the Coordenação de Aperfeiçoamento de Pessoal de Nível Superior - Brasil (CAPES) - Finance Code 001.

References

1. United Nations. The World's Cities in 2018—Data Booklet [Internet]. United Nations, Department of Economic and Social Affairs; 2018 [cited 2022 May 10] p. 34. Available from: https://www.un.org/development/desa/pd/sites/www.un.org.development.desa.pd/files/files/documents/2020/Jan/un_2018_worldcities_databooklet.pdf
2. Gomes EC de S, Silva IEP da, Nascimento WRC do, Loyo RM, Domingues ALC, Barbosa CS. Urban schistosomiasis: An ecological study describing a new challenge to the control of this neglected tropical disease. The Lancet Regional Health – Americas [Internet]. 2022 Apr 1 [cited 2025 Jan 29];8. Available from: https://www.thelancet.com/journals/lanam/article/PIIS2667-193X(21)00140-X/fulltext
3. World Bank. World Bank Open Data [Internet]. World Bank Open Data. [cited 2025 Jan 16]. Available from: https://data.worldbank.org
4. Salvador, Brazil Metro Area Population 1950-2025 [Internet]. [cited 2025 Jan 16]. Available from: https://www.macrotrends.net/cities/20275/salvador/population
5. Katz N. The discovery of Schistosomiasis mansoni in Brazil. Acta Trop. 2008 Dec;108(2–3):69–71.
6. Guimaraes IC, Tavares-Neto J. [Urban transmission of schistosomiasis in children from a neighborhood of Salvador, Bahia]. Rev Soc Bras Med Trop. 2006;39(5):451–5.
7. Blanton RE, Barbosa LM, Reis EA, Carmo TM, dos Santos CRA, Costa JM, et al. The Relative Contribution of Immigration or Local Increase for Persistence of Urban Schistosomiasis in Salvador, Bahia, Brazil. PLoS Neglected Tropical Diseases. 2015;9(3):e0003521.
8. Silva LK, Barbosa LM, Kovach JD, Dos Santos Teixeira R, Soares ÊS, Cardoso CW, et al. The changing profile of schistosomiasis in a changing urban landscape. Int J Parasitol. 2020 Jan;50(1):27–34.

9. Chaves CF, Sabino-Santos G, Cedraz FMA, Santos-Muccillo P, Filho JR, Zanardi VS, et al. Evidence for local transmission and maintenance of schistosomiasis in an urban neighbourhood in Northeast Brazil. Transbound Emerg Dis. 2022 Nov;69(6):3153–9.

10. Bernhard AE, Field KG. A PCR assay To discriminate human and ruminant feces on the basis of host differences in Bacteroides-Prevotella genes encoding 16S rRNA. Applied and environmental microbiology. 2000;

11. Feng S, Bootsma M, McLellan SL. Human-associated *Lachnospiraceae* genetic markers improve detection of fecal pollution sources in urban waters. Applied and Environmental Microbiology. 2018;

12. Harris PA, Taylor R, Minor BL, Elliott V, Fernandez M, O'Neal L, et al. The REDCap consortium: Building an international community of software platform partners. Journal of Biomedical Informatics. 2019 Jul 1;95:103208.

13. Epi Info^{TM} | CDC [Internet]. 2021 [cited 2022 May 18]. Available from: https://www.cdc.gov/epiinfo/index.html

14. Posit team. RStudio: Integrated Development Environment for R. Posit Software, PBC, Boston, MA [Internet]. RStudio: Integrated Development Environment for R. Posit Software, PBC, Boston, MA. [cited 2024 Oct 3]. Available from: https://www.posit.co/

15. VassarStats: Statistical Computation Web Site [Internet]. [cited 2025 Jan 16]. Available from: http://vassarstats.net/

16. QGIS Development Team 2024. Spatial without Compromise · QGIS Web Site [Internet]. QGIS Geographic Information System. Open Source Geospatial Foundation Project. [cited 2025 Jan 16]. Available from: https://qgis.org/

17. Barbosa LM, Silva LK, Reis EA, Azevedo TM, Costa JM, Blank WA, et al. Characteristics of the human host have little influence on which local *Schistosoma mansoni* populations are acquired. PLoS Negl Trop Dis. 2013;7(12):e2572.

18. Silva LK, Barbosa LM, Kovach JD, dos Santos Teixeira R, Soares ÊS, Cardoso CW, et al. The changing profile of schistosomiasis in a changing urban landscape. Int J Parasitol. 2020 Jan;50(1):27–34.

19. Calasans TAS, Souza GTR, Melo CM, Madi RR, Jeraldo V de LS. Socioenvironmental factors associated with *Schistosoma mansoni* infection and intermediate hosts in an urban area of northeastern Brazil. PLoS One. 2018;13(5):e0195519.

20. Zeleke AJ, Addisu A, Bayih AG, Tweya H, Timire C, Techilo W, et al. Does mass drug administration affect *Schistosoma mansoni* infection trends in West Dembia district, Northwest Ethiopia? J Infect Dev Ctries. 2020 Jun 29;14(6.1):72S-77S.

21. Bezerra DVF, Queiroz JW, Câmara VAV, Maciel BLL, Nascimento ELT, Jerônimo SMB. Factors Associated with *Schistosoma mansoni* Infestation in Northeast Brazil: A Need to Revisit Individual and Community Risk Factors. Am J Trop Med Hyg. 2021 Feb 16;104(4):1404–11.

22. Gryseels B, Polman K, Clerinx J, Kestens L. Human schistosomiasis. Lancet. 2006 Sep 23;368(9541):1106–18.

23. Nigo MM, Odermatt P, Salieb-Beugelaar GB, Morozov O, Battegay M, Hunziker PR. Epidemiology of *Schistosoma mansoni* infection in Ituri Province, north-eastern Democratic Republic of the Congo. PLoS Negl Trop Dis. 2021 Dec;15(12):e0009486.

24. Glidden CK, Singleton AL, Chamberlin A, Tuan R, Palasio RGS, Caldeira RL, et al. Climate and urbanization drive changes in the habitat suitability of *Schistosoma mansoni* competent snails in Brazil. Nat Commun. 2024 Jun 19;15(1):4838.

Figure Captions

Figure 1. Salvador, Bahia, Brazil map of all study sites. Pilot studies were conducted in all areas. Baseline complete cross-sectional surveys alone were conducted in three areas, and three neighborhoods had repeat longitudinal surveys.

Neighborhood	Abbreviations	Years	Initial Enrollment	Study Type
São Bartolomeu	SB2011, SB2015, SB2024	2011, 2015, 2024	1050	Pilot, longitudinal
Dique Do Cabrito	DC2017	2017	1253	Pilot, cross-sectional
Instituto Anísio Teixeira	IAT	2018	74	Pilot
Amazonas	AMZ	2018	67	Pilot
Saramandaia	SA2018, SA2020	2018, 2020	1786	Pilot, longitudinal
Pirajá	PI2019, PI2022	2019, 2022	1128	Pilot, longitudinal
Lagoa da Paixão	LP2022	2022	1035	Pilot, cross-sectional
Bate Facho	BF2023	2023	611	Pilot, cross-sectional

Figure 2. Initial prevalence of *S. mansoni* (SM) infections by age group and by neighborhood. SB2011 (São Bartolomeu 2011), DC2017 (Dique do Cabrito 2017), SA2018 (Saramandaia 2018), PI2019 (Pirajá 2019), LP2022 (Lagoa da Paixão 2022), BF2023 (Bate Facho 2023).

Figure 3. Age and *S. mansoni* (SM) infection by sex. Labels show the percent among those all those infected and grouped by age group and sex, e.g. 4.7% of all infections were boys 4-10 years old. SM – *S. mansoni*

Figure 4. Correlation between *S. mansoni* infection prevalence and distance from residence Salvador, 2011 - 2023. A. São Bartolomeu 2011 (SB2011); B. Dique do Cabrito 2017 (DC2017); C. Saramandaia 2018 (SA1018); D. Pirajá 2019 (PI2019); E. Lagoa da Paixão 2023 (LP2023). Buffer zones were created at 25 m and 100 m intervals from water contact points and the prevalence of *S. mansoni* infection calculated within each zone. *SM* mean prev. – *S. mansoni* mean prevalence. Brown points - residences with at least one *Sm*+ case. Green points - residences with no *Sm* case. * Chi-Square for trend (p <

0.05). Note: BF2023 had very few cases and this analysis could not be performed. Source: Open Street Map (https://www.openstreetmap.org/) modified in QGIS[16]

Figure 1

Click here to access/download;Figure;Figure 1.tif

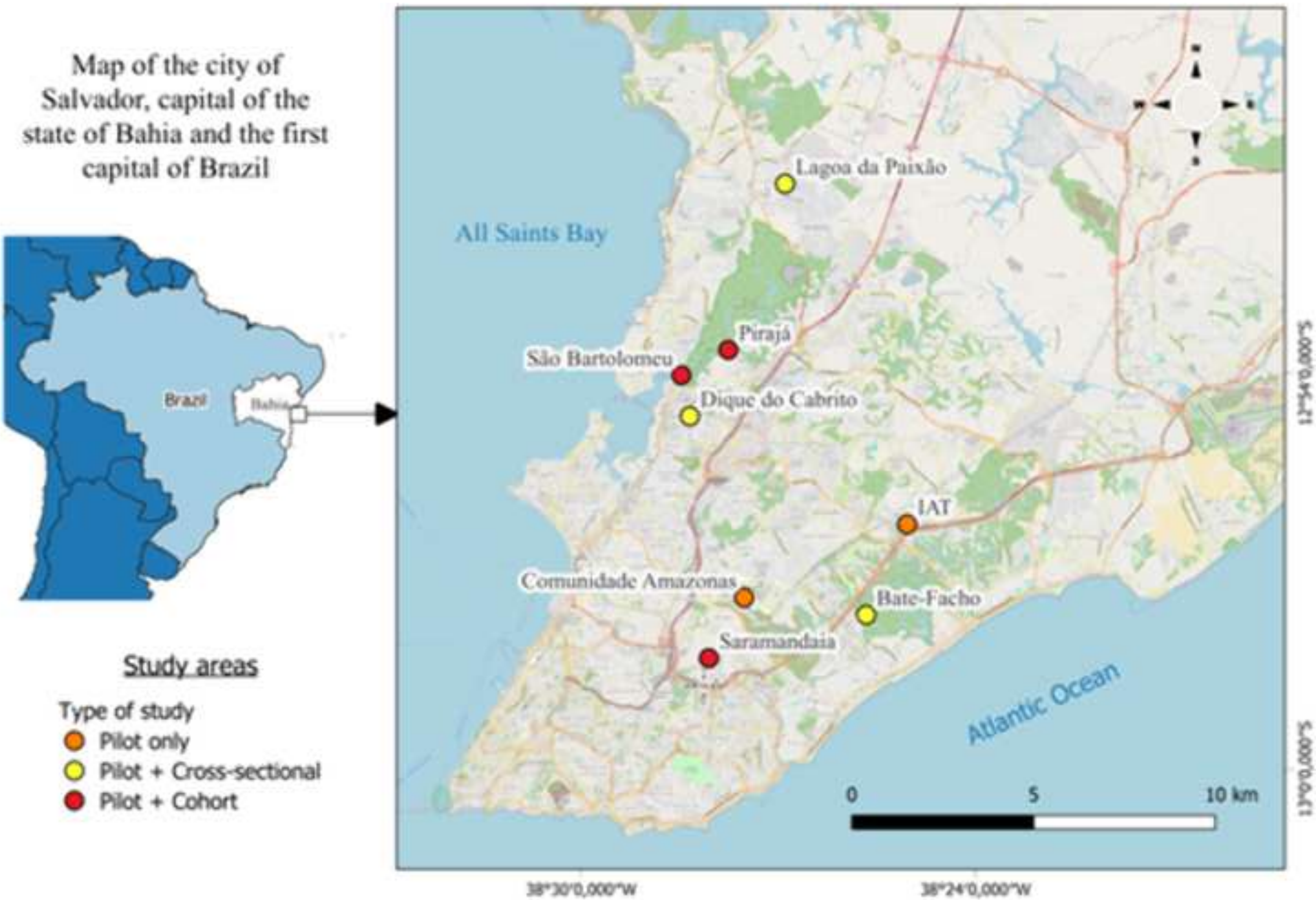

Figure 1 BW

Click here to access/download;Figure;Figure 1 BW.tif

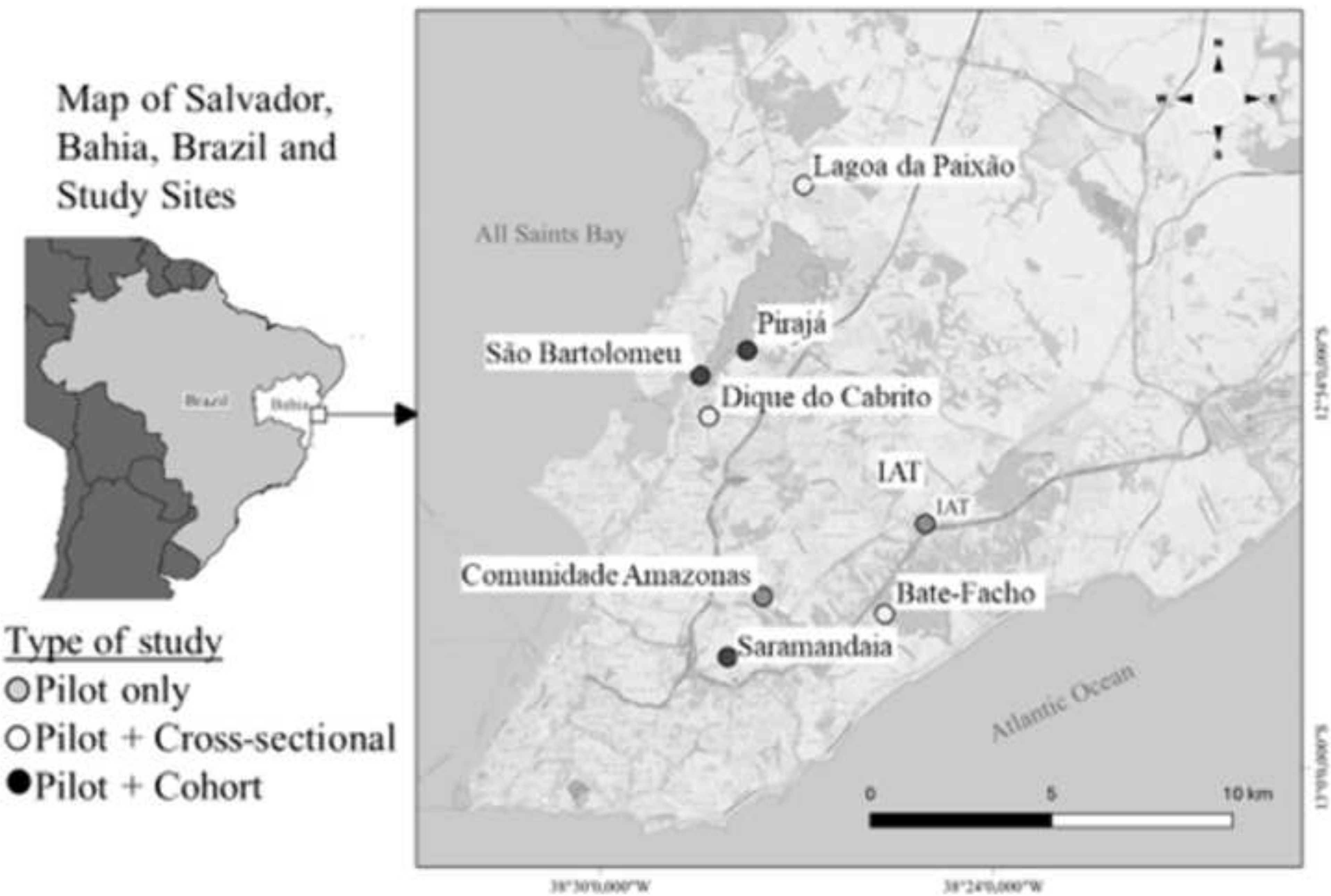

Figure 2

Click here to access/download;Figure;Figure 2.tif

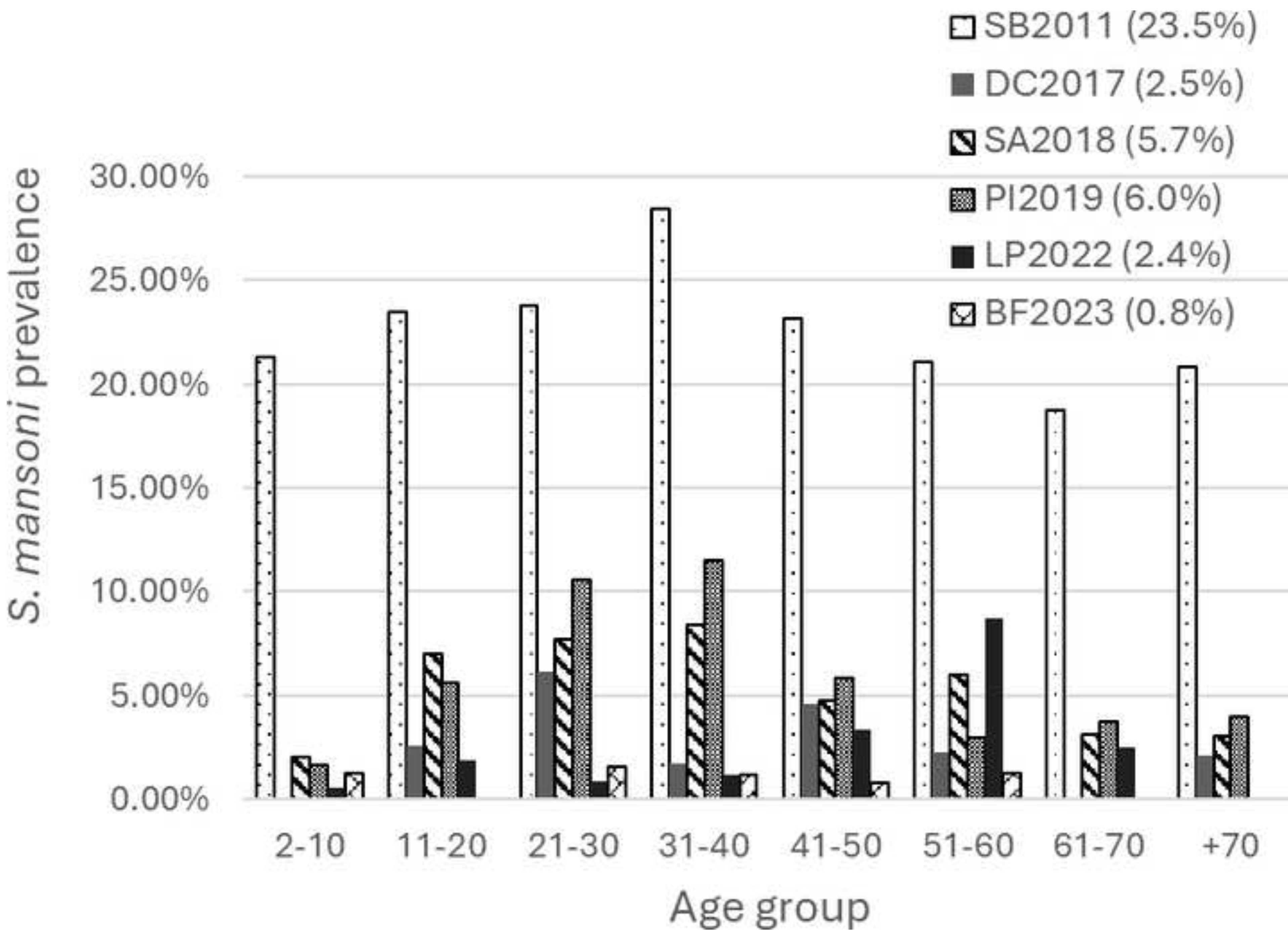

Figure 3

Click here to access/download;Figure;Figure 3.tiff

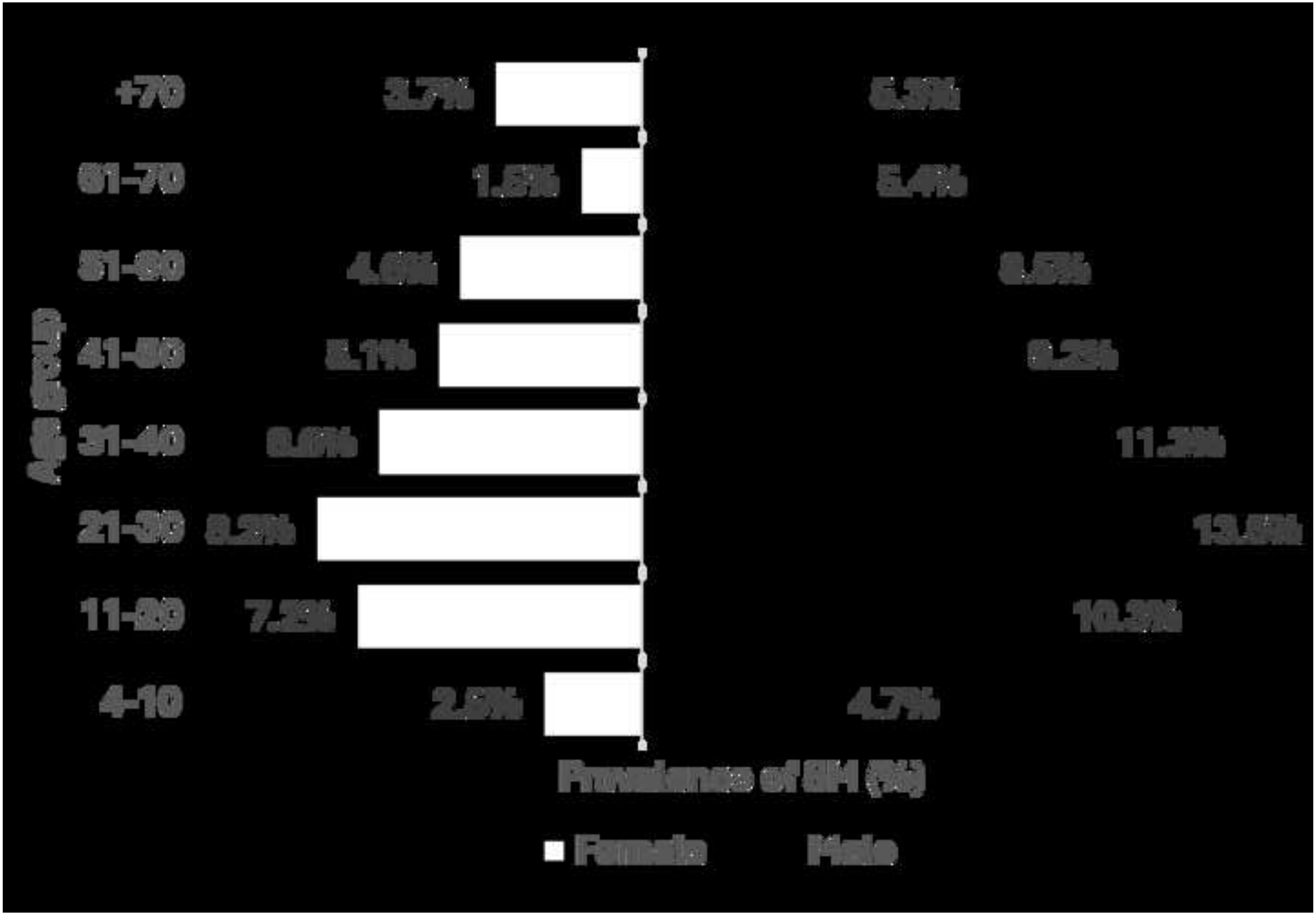

Figure 4

Click here to access/download;Figure;Figure 4.tif

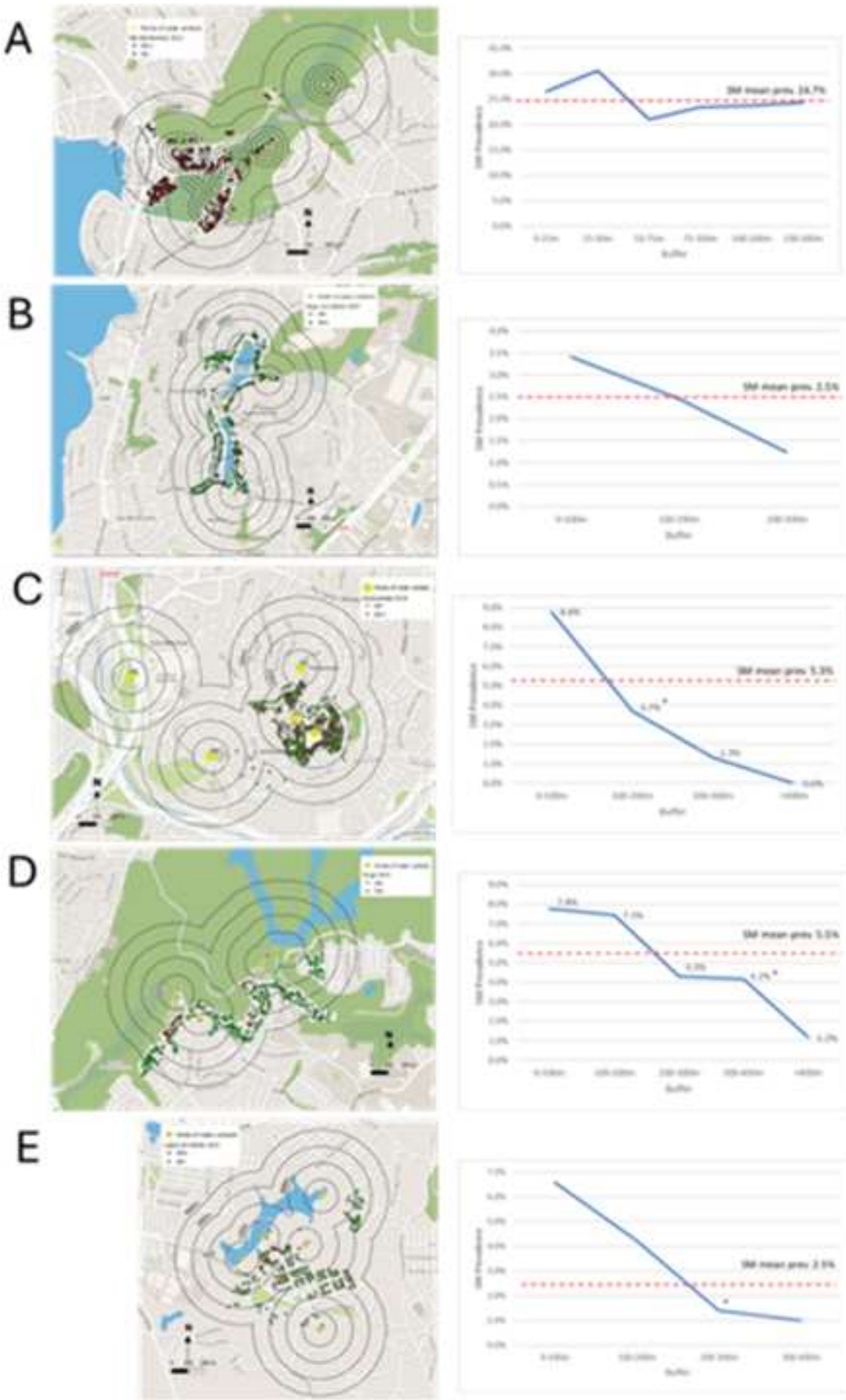

Figure 4 BW

Click here to access/download;Figure;Figure 4 BW.tif

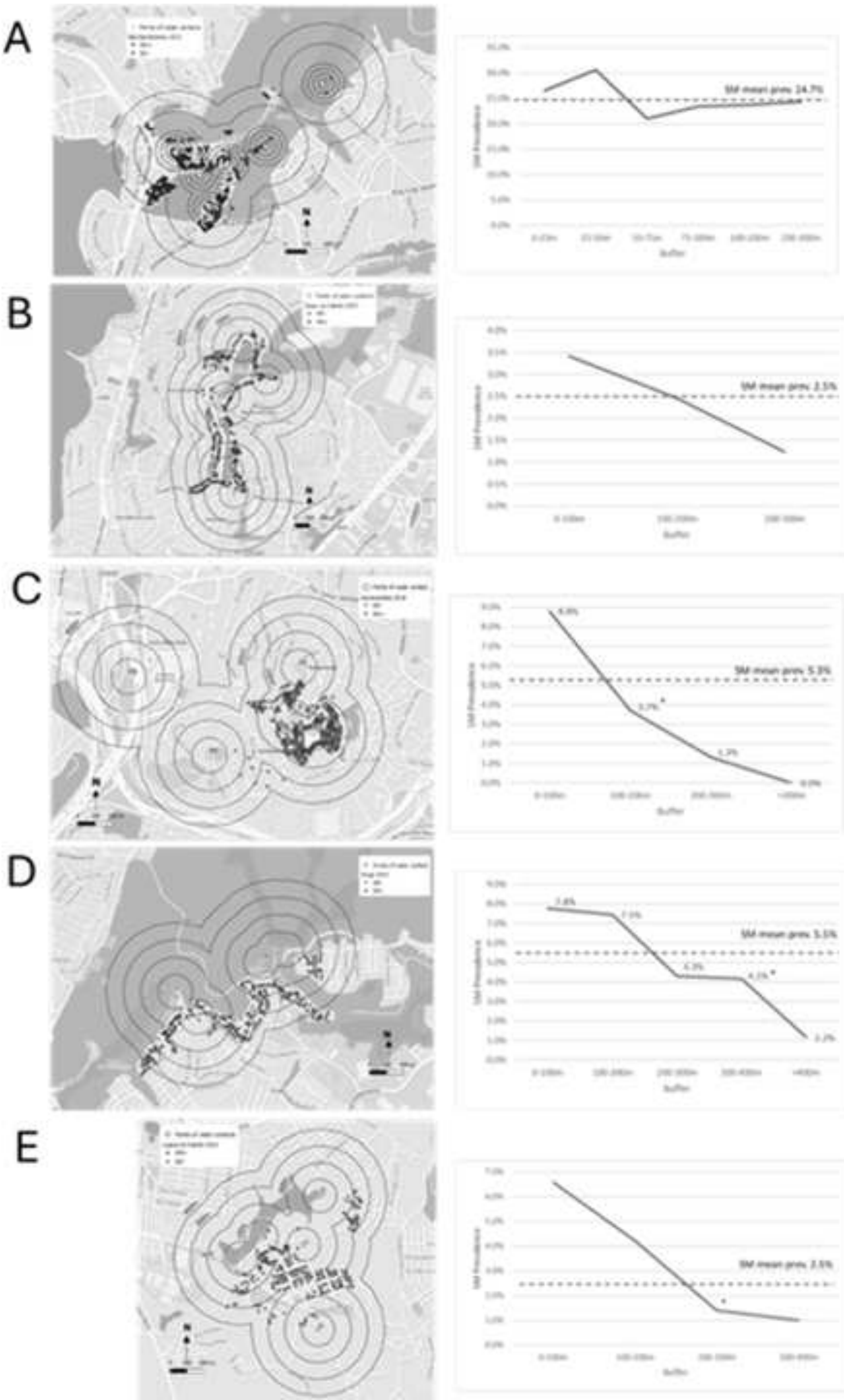

Tables

Table 1. Initial cross-sectional results in Salvador neighborhoods.

Characteristics	SB2011 (n = 1,050) % or mean ± SD	DC2017 (n = 1,253) % or mean ± SD	SA2018 (n = 1,786) % or mean ± SD	PI2019 (n = 1,128) % or mean ± SD	LP2022 (n = 1,035) % or mean ± SD	BF2023 (n = 611) % or mean ± SD	Total (N = 6,863) % or mean ± SD
Male sex	45·8%	41·7%	43·1%	41·1%	40·0%	42·7%	42·4%
Age	29·4 ± 17·4	32·8 ± 20·1	31·2 ± 19·0	34·0 ± 20·7	33·1 ± 20·4	38·3 ± 20·7	32·6 ± 19·7
Born in Salvador	78·7%	73·6%	59·1%	82·7%	79·7%	65·4%	72·3%
%Life in SSA	88·1 ± 19·9	94·0 ± 16·0	88·0 ± 23,0	88·0 ± 23·0	30·5 ± 19·6	85·0 ± 23·0	89·7 ± 20·7
Lived outside SSA	34·4%	17·8%	29·7%	8·5%	15·1%	24·7%	22·2%
Any travel outside last year	33·1%	39·9%	37·7%	19·9%	8·1%	43·9%	30·7%
Piped water	99·9%	96·3%	97·1%	99·6%	90·2%	99·1%	96·9%
Indoor Toilet	95·9%	98·4%	99·7%	99·0%	98·0%	100·0%	98·6%
Adequate sanitation[a]	67·8%	95·0%	95·2%	99·0%	100·0%	85·7%	92·0%
Sm prevalence	23·5%	2·5%	5·7%	6·0%	2·4%	0·8%	7·0%
Sm intensity of infection[b]	187·0 ± 384·3	43·1 ± 51·4	63·6 ± 107·1	84·6 ± 164·9	31·8 ± 23·7	28·8 ± 16·1	127·0 ± 294·3
Sm intensity of infection[c]	56·1	27·3	29·3	34·8	23·5	24·6	41·3

Neighborhoods: SB - São Bartolomeu; DC - Dique do Cabrito; SA - Saramandaia; PI - Pirajá; LP - Lagoa da Paixão; BF - Bate-Facho; *Sm* - *S· mansoni*.

[a] Municipal sewer connection or septic tank, [b] Arithmetic mean ± standard deviation (SD) [c] Geometric mean (eggs per gram of feces).

Table 2. Factors associated with the prevalence of *S. mansoni* in Salvador, 2011 to 2023.

Characteristics	N[a]	n	Prev.	Adjusted OR (95% CI)	p-value
Total	6,863	478	7·0%	-	
Study					
SB2011	1,050	247	23·5%	44·6 (14·2-140·6)	<0·0001
PI2019	1,128	68	6·0%	10·7 (3·4-34·4)	<0·0001
SA2018	1,786	102	5·7%	10·1 (3·2-32·0)	<0·0001
DC2017	1,253	31	2·5%	4·5 (1·4-14·8)	0·01
LP2022	1,035	25	2·4%	4·1 (1·2-13·7)	0·02
BF2023	611	5	0·8%	1	
Sex					
Male	2,913	264	9·1%	1·8 (1·5-2·2)	<0·0001
Female	3,950	214	5·4%	1	
Age, years					
≥ 21	4,579	340	7·4%	1·5 (1·2-1·9)	<0·0001
< 21	2,284	138	6·0%	1	
Born in Salvador					
Yes	4,944	347	7·0%	ns	-
No	1,893	126	6·7%		
Lived outside SSA					
Yes	1,364	108	7·9%	ns	-
No	5,364	362	6·7%		
Any travel outside last year					
Yes	2,076	139	6·7%	ns	-
No	4,681	327	7·0%		
Piped water					
No	205	10	4·9%	ns	-
Yes	6,270	430	6·9%		
Indoor Toilet					
No	56	0	0·0%	ns	-
Yes	5,991	440	7·3%		
Adequate sanitation					
No	516	102	19·8%	1·75 (1·3-2·3)	<0·0001
Yes	5,939	334	5·6%	1	

[a] Total varies according to the availability of data. Percentages are estimates over this total.

Undef - undefined, ns - not significant.

Table 3. Longitudinal cohort studies in Salvador.

Characteristics	SB2015 (n=1,104)	SB2024 (n=1,087)	SA2019 (n=833)	SA2023 (n=1,008)	PI2021 (n=1,048)	PI2023 (n=690)
	**% or mean ± SD**	**% or mean ± SD**	**% or mean ± SD**	**% or mean ± SD**	**% or mean ± SD**	**% or mean ± SD**
Male sex	41·8%	40·55%	42·7%	41·8%	41·6%	36·5%
Age	33·7 ± 198	38·2 ± 20·9	33·0 ± 19·7	36·1 ± 19·8	36·0 ± 21·1	39·3 ± 22·0
Born in Salvador	74·1%	91·4%	60·1%	61·4%	83·3%	82·9%
%Life in SSA	87·0 ± 21·0	96·0 ± 13·0	89·0 ± 21·0	89·0 ± 22·0	89·0 ± 21·0	91·0 ± 20·0
Lived outside SSA	25%	7·8%	29·7%	27·2%	9·7%	10·3%
Any travel outside last year	39·7	9·2%	36·1%	34·9%	16·8%	15·8%
Piped water	98·9	94·6%	98·9%	96·3%	98·4%	92·0%
Indoor Toilet	100·0	100·0%	99·6%	98·3%	97·9%	99·3%
Adequated sanitation	100·0	99·7%	93·9%	95·7%	99·4%	99·4%
Sm mansoni global prevalence	6·2%	4·5%	2·9%	1·7%	2·6%	1·9%
Sm intensity of infection[1]	162·7 ± 294·5	77·4 ± 160·5	74·2 ± 189·2	28·7 ± 33·7	90·4 ± 122·1	97·5 ± 107·0
Sm intensity of infection[2]	73·4	34·3	24·4	18·0	40·1	54·6
Sm incidence/year (%)	8·9%	0·3%	2·2%	0·4%	1·0%	0·4%
Sm intensity[a]	190·1 ± 376·5	122·7 ± 258·0	34·9 ± 47·0	21·3 ± 27·1	89·4 ± 117·7	71·3 ± 107·3
Sm intensity[b]	77·8	44·4	19·4	14·5	43·0	36·5
Sm prevalence new participants	4·5%	5·1%	6·4%	2·5%	1·8%	5·6%
Sm intensity[a]	129·1 ± 139·2	57·4 ± 88·8	139·6 ± 302·2	46·4 ± 44·3	96·0 ± 165·5	120·0 ± 109·6
Sm intensity[b]	68·5	30·6	35·8	30·1	27·0	77·0

Neighborhoods: SA = Saramandaia; PI = Pirajá; SB = São Bartolomeu; *Sm* = *S· mansoni*.
* Total varies according to the availability of data. Percentages are estimates over this total.
[a] Arithmetic mean ± standard deviation (SD) [b] Geometric mean (eggs per gram of feces).

Sao Bartolmeu 2011 & 2015

International Journal for Parasitology 50 (2020) 27–34

Contents lists available at ScienceDirect

International Journal for Parasitology

journal homepage: www.elsevier.com/locate/ijpara

The changing profile of schistosomiasis in a changing urban landscape

Luciano K. Silva [a], Lúcio M. Barbosa [b], Jeffrey D. Kovach [c], Rogério dos Santos Teixeira [d], Ênio Silva Soares [d], Cristiane Wanderley Cardoso [d], Rosângela Vasconcelos Martins Marques [e], Tatiane Pereira dos Santos [e], Mitermayer G. Reis [a,f,g], Ronald E. Blanton [c,*]

[a] Gonçalo Moniz Institute, Oswaldo Cruz Foundation, Salvador, Bahia, Brazil
[b] Bahiana School of Medicine and Public Health, Salvador, Bahia, Brazil
[c] Case Western Reserve University, Department of Pathology, Cleveland, OH, USA
[d] Municipal Secretariate of Health, Salvador, Bahia, Brazil
[e] Regional University of Bahia, (UNIRB), Salvador, Bahia, Brazil
[f] School of Medicine, Federal University of Bahia, Salvador, Bahia, Brazil
[g] Yale School of Public Health, Yale University, New Haven, CT, USA

ARTICLE INFO

Article history:
Received 25 June 2019
Received in revised form 24 September 2019
Accepted 1 October 2019
Available online 27 November 2019

Keywords:
Urbanisation
Population genetics
Elimination
Community-wide treatment
Sanitation
Differentiation
Effective population size

ABSTRACT

Since 2007, most of humanity resides in urban areas, a trend which continues worldwide. Diseases usually associated with rural contexts are now emerging or newly recognised in cities. In the neighbourhood of São Bartolomeu in Salvador, Brazil, the prevalence of *Schistosoma mansoni* infection in 2011 was >20%. Following enrollment and treatment of a portion of the community, ~25% of the area underwent urban renewal. In 2015, we returned to enrol individuals who had previously participated and a cohort that had not taken part in 2011. Thus, infected individuals in one group experienced specific drug treatment plus improved living conditions and the second group only improved living conditions. Between 2011 and 2015 there were no organised treatment programs, but adequate sanitation increased from 69% to 92% coverage, household flooding decreased, and the presence of indoor toilets increased to 99% of households. Ownership of household appliances also increased significantly. The overall prevalence of schistosome infections was 6.2%. In 2015, the cohort first seen in 2011 had a higher prevalence (8.7%) than those first seen in 2015 (4.8%) and showed a few demographic differences. The 2011 cohort was older, more likely born in Salvador, less likely to have lived outside of Salvador, spent a greater percentage of their lifetime in Salvador, but more likely to have travelled. The population structure of the parasites from both cohorts underwent a marked change with similar increased component and infrapopulation differentiation and >10 fold decrease in effective population size. There was a 4–5 year shift in age-specific prevalence in 2015 for all compared with 2011. While praziquantel may have helped reduce prevalence, our evidence suggests that the structural changes and improvements in living conditions had the biggest impact on schistosomiasis in this community.
© 2019 Australian Society for Parasitology. Published by Elsevier Ltd. All rights reserved.

1. Introduction

The increasing urbanisation of humanity is naturally producing the urbanisation of human infections. It is predicted that by 2050, 70% of the world will live in cities (United Nations, 2015), and currently 30% of urbanites in low or middle income countries live in informal settlements or slums. In some scenarios, the number is expected to triple by 2050 (United Nations, 2013). Schistosomiasis is often considered a disease of rural populations, but there have always been infections with schistosomes in cities. Now the cities themselves are changing and, with them, the profile of the disease. Urbanisation of schistosomiasis is recognised as a growing problem in Africa (Sarda et al., 1985; Ernould et al., 2000; Matthys et al., 2010; Mwakitalu et al., 2014; Dabo et al., 2015), and it has been well documented in Brazil (Barreto, 1960; Guimaraes et al., 1993; Barbosa et al., 2011, 1998; Guimaraes and Tavares-Neto, 2006; Coura-Filho, 2005; Oliveira et al., 2013; Pinto et al., 2013; Blanton et al., 2015; Calasans et al., 2018;). Studies in Brazil (Brooker et al., 2006) and China (Shen et al., 1997) indicate there are significant differences between rural and urban schistosomiasis. Urbanisation and urban landscapes offer obstacles, and opportunities sometimes differ from rural areas. While population

* Corresponding author at: Tulane School of Public Health and Tropical Medicine, 1440 Canal Street, New Orleans, LA 70112 USA.
E-mail address: rblanton1@tulane.edu (R.E. Blanton).

https://doi.org/10.1016/j.ijpara.2019.10.003
0020-7519/© 2019 Australian Society for Parasitology. Published by Elsevier Ltd. All rights reserved.

density and violence can be obstacles to control infection in the urban environment, population density may facilitate access to those infected, environmental modifications of these artificial sites are more easily and comprehensively accomplished, urban populations are often better off economically and have access to more resources (World Bank. Global Monitoring Report, 2013).

We have previously identified the neighbourhood of São Bartolomeu as an urban focus of schistosomiasis in the city of Salvador, Brazil (Guimaraes and Tavares-Neto, 2006; Blanton et al., 2015). In this neighbourhood, the prevalence in 2011 was 25% with 13% of these heavy infections defined as >400 eggs per gram (epg) of stool. In 2011, the project enrolled 1500 residents from areas with the highest prevalence based on a previous survey of school aged children (Guimaraes et al., 1993). Egg-positive individuals were treated with praziquantel and albendazole, for schistosomiasis and other helminths, respectively, and all those treated for *S. mansoni* were egg negative when examined 4–6 weeks later. Subsequently, the area underwent a period of urban renewal. Approximately 20% of the homes and inhabitants were removed and replaced with two-storey public housing with connections to the municipal sewer system. In 2015, the Municipal Health Department in collaboration with the Oswaldo Cruz Foundation Bahia (Brazil) initiated a program for control or elimination of schistosomiasis in São Bartolomeu employing methodology similar to that of our previous program from 2011. We evaluated the relative impacts of demographic changes, changes in sanitation and a previous mass treatment on the prevalence of *S. mansoni* infection and its population structure in the urban setting of São Bartolomeu, Salvador-BA, Brazil.

2. Materials and methods

2.1. Study site and population

The neighbourhood of São Bartolomeu (12° 54.141′ S, 38° 28.514′ W) borders a city park (São Bartolomeu Park) located in the northwestern part of Brazil's fourth largest city, Salvador. In addition to an important historical site, the park forms a natural buffer between the densely populated portions of the city and a reservoir that until recently supplied drinking water for the city. In the 1970s the region was occupied by people coming from rural areas of the state, looking for work and better living conditions. In 2011, there were ~3600 residents within the 0.23 km^2 geographic area of São Bartolomeu. One-third of this area is occupied by mangrove swamp where the Cobre River flows through the community on its way to the sea. In 2004 and again in 2011, an urban focus of schistosomiasis was identified in this neighbourhood (Guimaraes and Tavares-Neto, 2006; Blanton et al., 2015). In 2012, the state of Bahia implemented a project to improve housing and revitalise the park. People who built their residences inside the park and residents of some neighbourhood houses were relocated to other areas in the city of Salvador. Some returned later to newly constructed houses with satisfactory sanitation infrastructure. Of those who participated in the 2011 study, 549 were again identified and participated in 2015 (Fig. 1). In addition, 1105 residents were examined who did not participate in 2011.

2.2. Study design, data and stool collection

In order to understand the effect of environmental and demographic changes on parasite populations, the study design included both a cohort and a cross-sectional study design. A focused study of the community was conducted in 2011 in which 1224 residents within a defined section of the neighbourhood answered a questionnaire and had stool examined on three different days for parasitic infections. This group has been previously described (Blanton et al., 2015). The total group c renewal in 2011 will be designated 2011T, and the total group examined post-urban renewal in 2015 will be 2015T. Further, the cohort examined in 2015 and previously seen in 2011 will be designated 2011C, and those examined only in 2015 will be 2015C (Fig. 1). In 2015, all households were approached by trained interviewers from the Department of Social Service of the Regional University of Bahia, Salvador. Study data was entered in a Research Electronic Data Capture (REDCap) database (Harris et al., 2009) hosted at the Oswaldo Cruz Foundation. Each household was also georeferenced for spatial analysis.

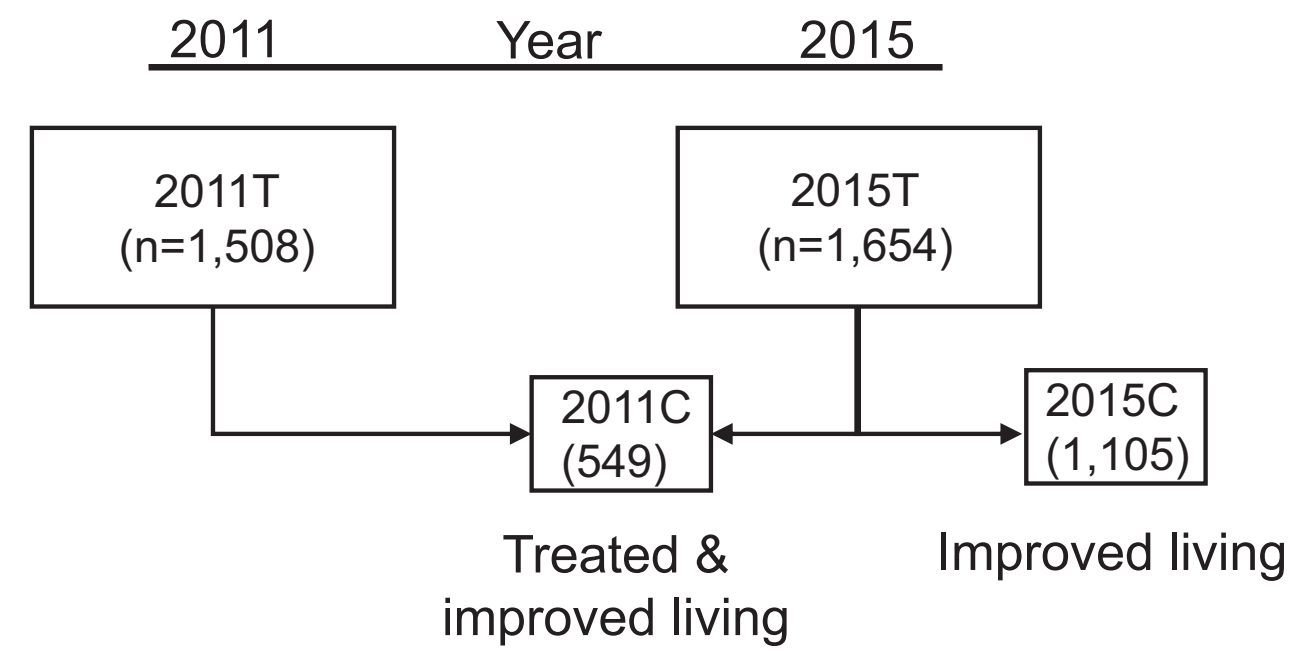

Fig. 1. Study Flow Chart. The shaded area represents samples collected in 2011; the unshaded area 2015. 2011T – all of the individuals enrolled, examined and if positive for *Schistosoma mansoni* in at least one of three stool tests, treated in 2011. 2015T – All of the individuals enrolled, examined and if positive for *S. mansoni* in at least one of three stool tests, treated in 2015. The 2015T group was composed of two cohorts. 2011C – the cohort that had been enrolled previously in 2011 and 2015C – the cohort first enrolled in 2015.

The Committee on Ethics in Research of the Oswaldo Cruz Foundation of Salvador, Bahia, the Brazilian National Committee on Ethics in Research and the Institutional Review Board for Human Investigation of University Hospitals Case Medical Center, Cleveland, Ohio, USA, approved the study design. All subjects provided written informed consent or in the case of minors, consent was obtained from their guardians. All aspects of the study have been conducted according to the principles expressed in the Declaration of Helsinki.

2.3. Infection detection and egg isolation

After collecting demographic data, each individual was asked to provide stool samples on three different days. All samples were examined for *S. mansoni* or geohelminth ova by qualified laboratory technicians using the Kato-Katz technique. Eggs were concentrated from a whole stool as described previously (Blanton et al., 2011). Briefly, stool was liquefied in 2% saline and eggs concentrated by selective sieving followed by a saline sedimentation. Concentrated sediment was kept at −20 °C until further analysis.

2.4. DNA extraction and genotyping

DNA was extracted from the sediment by a standard phenol: chloroform protocol and treated with hexadecyltrimethylammonium bromide bromide (CTAB) to remove PCR inhibitors (Ausubel, 1987). For genotyping, 2 µl of a 200 µl final volume of DNA were used for PCR with each set of nine primer pairs to amplify their corresponding microsatellite locus. Amplicons from this reaction were then multiplexed with 2–3 other amplified loci for capillary electrophoresis and genotyping on an Applied Biosystems 3730xl DNA Analyzer (Applied Biosystems, Carlsbad, CA, USA). Peaks were analysed with PeakScanner software version 2.0 (Applied Biosystems, Carlsbad, CA, USA). Non-matching duplicates, peaks not conforming to the step-wise mutation model

and peaks <100 pixels in height were eliminated. Jost's D (Jost, 2008) between replicates was calculated, and where this was >0.01, they were re-examined or eliminated. Allele frequencies were calculated based on the ratio between individual peak heights and the sum of peak heights for each marker (Blanton et al., 2011).

2.5. Data analysis

Descriptive, univariate analysis and multivariate logistic regression statistics were performed in Epi Info 7.2.2.6 (https://www.cdc.gov/epiinfo/index.html). Continuous variables were evaluated with the Kruskal-Wallis test. Categorical comparisons were performed by a chi-square test using Yates' correction or a Fisher-Exact test when appropriate. A two-sided P value <0.05 was considered significant for all analyses. Effect sizes were expressed as odds ratios (ORs) and Hedges' g for chi-square and Student's T-test, respectively. Socioeconomic variables were used to determine economic classification based on the Criteria for Economic Classification Brazil of the Brazilian Association of Research Companies, São Paulo (Criteria for Economic Classification Brazil] 2013. http://www.abep.org/Servicos/Download.aspx?id=03).

For genetic analyses, Jost's D was calculated with the software Spade (Chao and Shen, 2010. https://chao.shinyapps.io/SpadeR/) and effective population size (Ne) with MLNe (Arntzen and Wallis, 2006). Sensitivity analysis was performed for all population genetic indices by iteratively removing one marker and re-calculating these values to determine if one marker skewed the result.

3. Results

3.1. Total and cohort population characteristics

The population in 2011 and 2015 showed few demographic differences (Table 1). The 2011 population was slightly younger on average, had lived a greater percentage of their life in Salvador and travelled less outside of the city. The effect sizes of these differences were small, however. Households without refrigerators went from 6% to 3% (P < 0.05, OR, 2.1), and those with computers rose from 25% to 35% (P < 0.01, OR, 1.7), which suggests an improvement in disposable income. The major differences between the populations in 2011 and 2015 were for living conditions and *S. mansoni* infection. In 2011, fewer residents had piped water or adequate sewage disposal, and more homes were flooded.

The 2011C cohort consisted of 549 of the 1239 (44%) individuals that participated in 2011, while there were 1105 in the 2015C group. In 2015, the 2011C cohort differed from the 2015C cohort by being on average slightly older and living a greater percentage of their lifetime in Salvador (Table 2). A smaller percentage had lived outside Salvador, but a larger percentage had travelled outside of the city in the preceding year. None of these variables had an OR >2 or <0.5 or Cohen's d >0.2. The differences with the largest effect size between the 2011C and 2015C cohorts were the prevalence and intensity of infection.

3.2. Infection and risk factors

Of the 302 *S. mansoni* infections identified, 29 were not treated either because they moved or had a contraindication. Of the 273 treated, 187 were negative on follow-up, four were persistently positive and 82 failed to have repeat examinations. The community-wide prevalence decreased four-fold (24% to 6%) between 2011 and 2015. The decrease in prevalence was most marked in the youngest children, 0–10 years old. In addition, while the peak prevalence in 2011 was in 11–15 year olds, in 2015 the peak prevalence shifted 5 years to 16–20 year olds (Fig. 2). The intensity was lower for most age groups in 2015, although the average was not different.

The incidence or reinfection rate over this 4 year period was 1.7% per year. When examined in 2015, the prevalence and intensity of the 2011C group were higher than the 2015C cohort. We found 116 individuals who were not present in the neighbourhood in 2011, but only 20 had immigrated in the last 5 years, and the rest had been born since 2011. The prevalence of *S. mansoni* infection was no different in these recent immigrants than that of long-term residents.

While male sex, younger age, birth in Salvador, never living or travelling outside Salvador and poor sanitation were associated with infection in 2011, only male sex and living and travelling outside of Salvador were risk factors in 2015 (Supplementary Table S1). Infected individuals in 2011 were less likely to have lived outside Salvador, less likely to have travelled outside the city or to have adequate sanitation compared with those infected in 2015

Table 1
Characteristics of all São Bartolomeu, Salvador, Brazil, study participants in 2011 and 2015.

Characteristics	2011			2015			P[b]	Effect size[c]	95% CI[e]
	Total[a]	*n*	% or Mean ± S.D.	Total[a]	*n*	% or Mean ± S.D.			
Male Sex	1508	721	48%	1654	739	45%	ns	0.88	0.77–1.01
Age	1507		29.1 ± 17.4	1561		31.9 ± 19.3	<0.05	0.15[d]	–
Born in Salvador	1508	1179	78%	1494	1146	77%	ns	0.92	0.77–1.09
% life in Salvador	1508		90.8 ± 20.1	1479		88.4 ± 20.6	<0.05	0.12[d]	
Lived outside Salvador	1508	336	22%	1470	369	25%	ns	1.17	0.99–1.38
Any travel outside last year	1499	496	33%	1467	560	37%	<0.05	1.25	1.07–1.45
Piped water	1329	1322	99%	1509	1494	99%	ns	–	–
Indoor toilet	1315	1262	96%	1502	1480	99%	<0.01	2.83	1.71–4.67
Adequate sanitation[f]	1297	890	69%	1496	1383	92%	<0.01	5.60	4.47–7.01
Flooding	1505	742	49%	1480	460	31%	<0.01	0.46	0.40–0.54
S. mansoni prevalence	1224	302	24.7%	1158	72	6.2%	<0.01	0.20	0.15–0.27
S. mansoni intensity[h]	302		206.3 ± 508.6	71		159.2 ± 286.9	ns	0.11	–

[a] Total varies according to availability of data. Percentages are estimates over this total.
[b] Two-tailed P value. ns, not significant. For categorical variables, Yates; chi-square test or Fisher-Exact test were used when appropriate. For continuous variables, a Kruskal-Wallis test for two groups for used.
[c] Odds ratio for categorical variables.
[d] Hedges' g, standardised means for continuous variables where <0.2 is conventionally considered a small effect.
[e] Confidence interval
[f] Connection to septic tank or sewer, otherwise toilet flushes to rivers or streams.
[h] Mean *Schistosoma mansoni* eggs per gram of feces.

Table 2
Characteristics of study cohorts in 2015.

Characteristics	2011C[a]			2015C			Effect size		95% CI[f]
	Total[b]	*n*	% or Mean ± S.D.[c]	Total[b]	*n*	% or Mean ± S.D.	*P*[c]	OR[d] or Hedge's g[e]	
Male Sex	549	244	44	1105	495	45	ns	0.99	0.80–1.21
Age	543		33.5 ± 17.7	1018		31.4 ± 20.0	ns	0.11[e]	
Born in Salvador	538	428	80	956	718	75	ns	1.2897	0.99–1.67
% life in Salvador	535		90.9 ± 17.8	944		86.9 ± 21.9	ns	0.20[e]	
Lived outside Salvador	535	111	21	935	258	28	<0.01	1.46	1.13–1.88
Any travel outside last year	527	358	32	940	549	42	<0.05	0.66	0.53–0.83
SES (Estimated mean family income)									
Status B/C (R$4,418–1024/month)[g]	524	313	60	985	579	59	ns	0.96	0.77–1.19
Status D/E (R$714–447/month)[g]		211	40		406	41		–	–
Piped water	524	523	100	980	966	99	ns	–	–
Indoor toilet	523	511	98	974	964	99	ns	–	–
Adequate sanitation[h]	522	490	94	969	888	92	ns	0.72	0.47–1.09
Flooding	537	181	34	943	279	30	ns	0.83	0.66–1.04
Schistosoma mansoni prevalence	424	37	8.7	734	35	4.8	<0.05	0.52	0.32–0.85
S. mansoni intensity (epg)[i]	424		190.1 ± 376.5	734		125.5 ± 133.1	ns	0.26[e]	

[a]2011C – the cohort first examined in 2011 with conditions of 2015; 2015C – the cohort first examined in 2015; all cohorts in São Bartolomeu, Salvador, Bahia, Brazil.
[b]Total varies according to availability of data. Percentages were calculated over this total.
[c]SES, social economic status (Brazilian Association of Research Companies, 2013).
[c]Two-tailed *P* value; ns, not significant. For categorical variables; Yates' chi-square test or a Fisher-Exact test were used when appropriate. For continuous variables, a Kruskal/Wallis test for two groups was used.
[d]Odds ratio
[e]Standardised means (Hedges' g) for continuous variables where <0.2 is conventionally considered a small effect.
[f]Confidence interval
[g]Status – Brazilian Association of Research Companies economic status classification
[h]Connection to septic tank or sewer, otherwise toilets flush to rivers or streams.
[i]Epg log-transformed mean of parasite eggs per gram

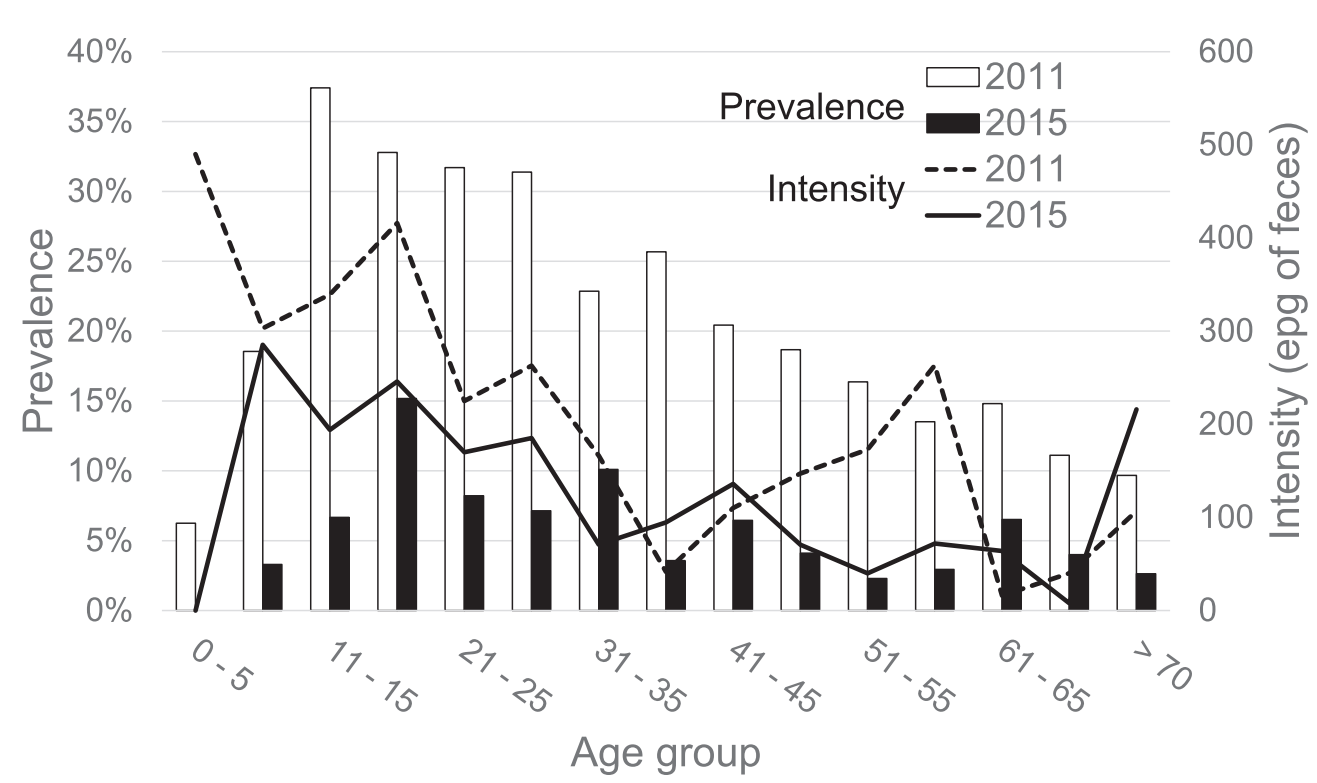

Fig. 2. Studies in 2011 and 2015; age-specific *Schistosoma mansoni* infection prevalence and intensity. The prevalence and intensity (arithmetic average –eggs per gram of feces (epg)) of *S. mansoni* infection among participants found to be infected in respective years.

(Table 3). Except for sanitation, the effect sizes for these variables were relatively small. Travel outside Salvador and the percentage of lifetime lived in Salvador were not associated. Those infected in the two cohorts differed in that the 2011C cohort was younger, more likely born in Salvador, spent a longer percentage of their lifetime in Salvador, were less likely to have ever lived outside of Salvador and more likely to have travelled outside of the city. The effect sizes were modest, except for the age difference (Table 4). Infection risk factors in 2015 by cohort are provided in Supplementary Table S1.

3.3. Parasite population structure

Parasite population structure can be partitioned as those parasites within an individual host (infrapopulation) or those within the whole community of hosts (component population). Component populations can represent geographic or temporal groupings.

Table 3
Comparison of *Schistosoma mansoni*-infected residents of São Bartolomeu, Salvador, Bahia, Brazil in 2011 and 2015.

Study	2011T[a]			2015T[a]			Comparisons		
Characteristics	Total[b]	*n*	%	Total	*n*	%	*P*[c]	OR[d]	95% CI[e]
Male sex	302	202	66.9	72	53	73.6	ns	0.72	0.40–1.29
Age, years	302		26.6 ± 16.1	62		30.3 ± 14.6	ns[f]		
Born in Salvador	302	253	83.8	63	52	82.5	ns	1.69	0.87–3.29
% life in Salvador	302		94.6 ± 14.7	62		90.6 ± 20.4	ns[f]		
Lived outside Salvador	302	46	15.2	62	10	16.1	<0.05	0.48	0.24–0.96
Any travel last year	302	77	25.6	60	16	26.7	<0.05	0.53	0.30–0.96
Adequate sanitation[g]	302	152	60.6	61	54	88.5	<0.01	0.13	0.06–0.30
Flooding	302	146	48.5	62	18	29.0	ns	0.83	0.47–1.47
Occupational water contact	298	82	27.5	58	5	8.6	ns	2.04	0.78–5.38

[a] All individuals infected with *S. mansoni* in 2011 (2011T) and all infected in 2015 regardless of prior treatments (2015T).
[b] Total varies according to availability of data. Percentage is an estimate over this total.
[c] Pearson's or Yates' corrected chi-square test; two-tailed *P* value. ns, not significant.
[d] Odds ratio.
[e] Confidence interval.
[f] Hedges' g, standardised means for continuous variables where <0.2 is conventionally considered a small effect.
[g] Connection to septic tank or sewer, otherwise toilets flush to rivers or streams.

Table 4
Comparison between praziquantel treated cohorts and non-examined, non-treated cohorts of *Schistosoma mansoni*-infected residents from São Bartolomeu, Salvador, Bahia, Brazil in 2015.

Study	2011C[a]			2015C			Comparisons		
Characteristics	Total[b]	*n*	%	Total	*n*	%	*P*[c]	Effect size[d]	95% CI[f]
Male sex	37	27	73.0	35	26	74.3	ns	1.07	0.37–3.06
Age	37		25.4 ± 14.4		26	36.1 ± 16.4	<0.01	0.70[e]	
Born in Salvador	36	33	91.7	27	19	70.4	<0.05	0.22	0.05–0.91
% life in Salvador	36		96.3 ± 6.9	26		82.8 ± 28.9	<0.05	0.70[e]	
Lived outside Salvador	36	2	5.6	26	8	30.8	<0.05	7.56	1.45–39.40
Any travel last year	34	10	29.4	26	6	23.1	<0.05	0.36	0.14–0.91
Adequate sanitation[g]	35	32	91.4	26	22	84.6	ns	0.52	0.10–2.53
Flooding	36	13	36.1	26	5	19.2	ns	0.42	0.13–1.38
Occupational water contact	35	3	8.6	23	2	8.7	ns	1.02	0.16 – 6.60

[a] Only those infected with *S. mansoni* for the cohort first examined in 2011 with conditions of 2015 (2011C); the cohort first examined in 2015 (2015C).
[b] Total varies according to availability of data. Percentage is an estimate over this total.
[c] Pearson's or Yates' corrected chi-square test or a Fisher's exact were used where indicated; two-tailed *P* value. ns, not significant.
[d] Odds ratio
[e] Hedges' g, standardised means for continuous variables where <0.2 is conventionally considered a small effect.
[f] Confidence interval
[g] Connection to septic tank or sewer, otherwise toilets flush to rivers or streams.

There was a high degree of genetic differentiation between the component populations in 2011 and 2015 (Table 5). A similar high degree of component population differentiation (Dc) exists between the cohorts 2011C and 2015C, and the total component population 2011T. The two cohorts had similarly low differentiation from the total 2015 component population. Between the two cohorts examined in 2015, however, there was a small degree of differentiation above that expected (Dc = 0.023), suggesting they were not completely drawn from the same genetic pool. (Fig. 3). Mean pairwise comparisons between all 2011 infrapopulations (Di) was 0.071 ± 0.063 (Table 6). In 2015, the mean Di increased to 0.183 ± 0.178. The mean Dis within each cohort in 2015 were 0.164 ± 0.161 for 2011C and 0.197 ± 0.194 for 2015C. Ne decreased more than 10 fold (Table 7). Sensitivity analysis resulted in less than 1% difference between values averaged after removing one microsatellite versus using all 10 markers. Microsatellite marker characteristics and Di by marker and cohort are provided in Supplementary Tables S2 and S3. Infrapopulation allele numbers for 2011 and 2015 are included in Supplementary Data S1 and S2, respectively.

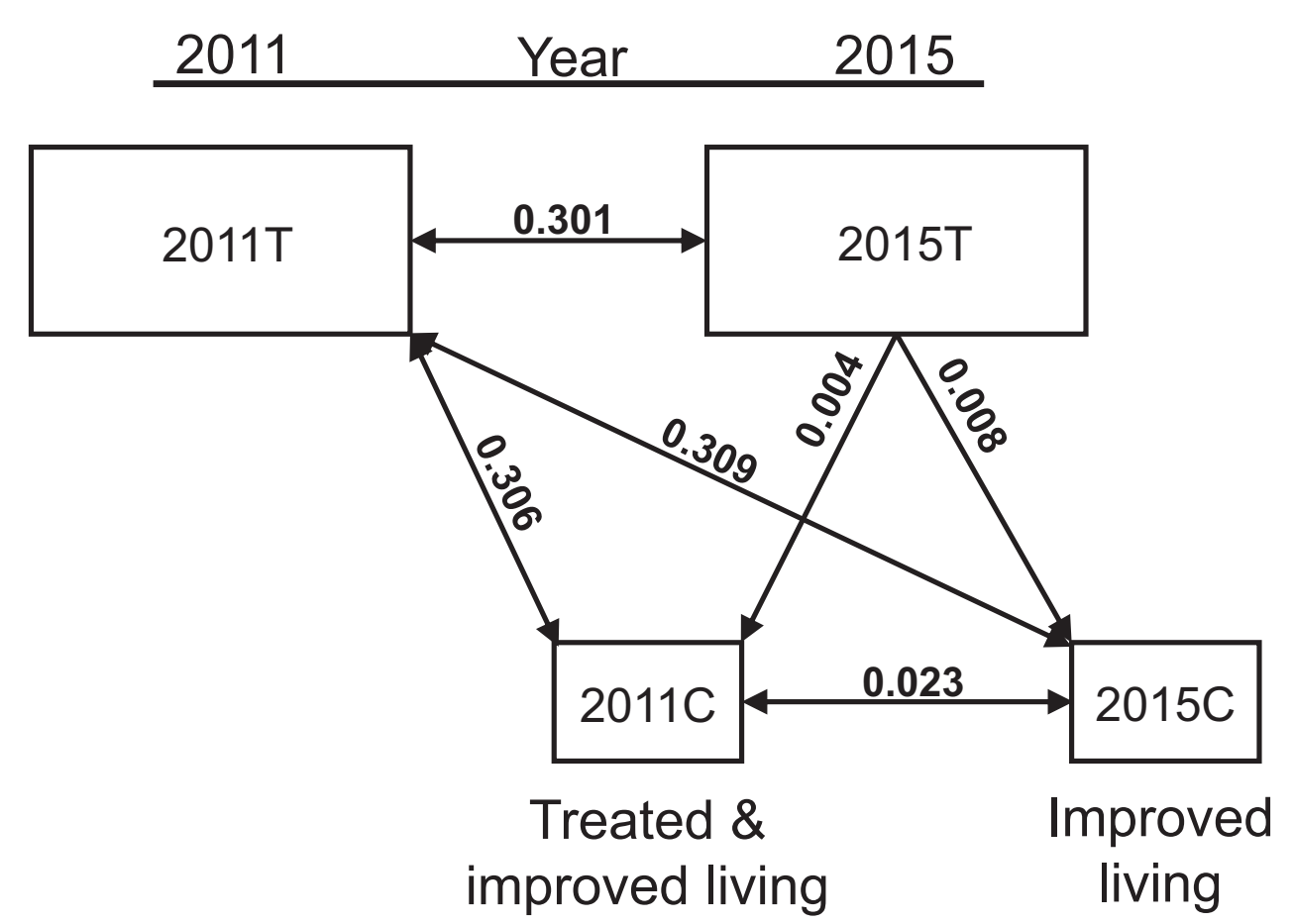

Fig. 3. Differentiation (Dc) between component populations and sub-populations surveyed in 2015. The shaded area represents samples collected in 2011; the unshaded area 2015. 2011T – parasites from all *Schistosoma mansoni*-infected individuals examined in 2011. 2015T – parasites from all *S. mansoni*-infected individuals examined in 2015. 2011C – parasites collected in 2015 from individuals enrolled in 2011. 2015C – parasites collected in 2015 from individuals not previously enrolled.

3.4. Geographic distribution

Households with at least one infected occupant were dispersed throughout the sampled area among houses with no one infected (Fig. 4). In 2011, there was a slightly higher prevalence in the areas inside the park. Houses were removed from within the park after 2011, and between 2011 and 2015, new standardised housing replaced informal structures north of the mangrove. For the 2015 survey, a new area was included south of the mangrove swamp that occupies the center of the community. In 2015, households with infected occupants were again dispersed throughout the community with no clear focal hotspots.

Table 5
Schistosoma mansoni component population differentiation.

Dc[a] between year and cohorts			
	2015T	2011C	2015C
2011T	0.301	0.306	0.303
2015T		0.004	0.008
2011C			0.023

2011T – all subjects examined in 2011 prior to urban renewal.
2015T – all subjects examined in 2015 post urban renewal.
2011C – the cohort first examined in 2011 and reexamined in 2015 post urban renewal.
2015C – the cohort first examined in 2015 post urban renewal.
[a] The mean Jost D between total allele frequencies for populations composed of all subjects examined in 2011 or 2015 or cohorts within the population examined in 2015, all from São Bartolomeu, Salvador, Bahia, Brazil.

4. Discussion

By definition, population density is higher in urban than rural areas, and in Brazil this also implies physical and social differences. In urban areas, the population density, education level and base income are higher, as is violence. There is less contact with surface waters for most urbanites (Ernould et al., 2000). Urban areas have greater health resources, and environmental interventions that directly affect a large number of people are more easily accomplished. For schistosomiasis, some clinical features of urban disease set it apart from rural disease. In urban areas, infections have lower intensity and an older age distribution. In common with infections in rural areas, there is significant spatial clustering, although the focal nature of the disease is even more evident over short distances in the urban environment (Brooker et al., 2006).

Table 6
Schistosoma mansoni population structure compared.

	Di[a]	S.D.
2011T	0.071	0.063
2015T	0.183	0.178
2011C	0.164	0.161
2015C	0.197	0.194

2011T – all subjects examined in 2011 prior to urban renewal.
2015T – all subjects examined in 2015 post urban renewal.
2011C – the cohort first examined in 2011 and reexamined in 2015 post urban renewal.
2015C – the cohort first examined in 2015 post urban renewal.
[a] The mean pairwise Jost D between infrapopulations for all subjects examined in 2011 or 2015 or cohorts within the population examined in 2015, all from São Bartolomeu, Salvador, Bahia, Brazil.

Table 7
Schistosoma mansoni population structure compared.

	Ne[a]	(95%CI[b])
2011T	19,342	(29,497–3850)
2015T	1437	(1410–1453)
2011C	1079	(1062–1093)
2015C	1185	(1161–1206)

2011T – all subjects examined in 2011 with conditions of 2011.
2015T – all subject examined in 2015 with conditions of 2015.
2011C – the cohort first examined in 2011 with conditions of 2015.
2015C – the cohort first examined in 2015.
[a] Effective population size.
[b] Confidence interval.

Urbanisation has been a key global process in the last century and promises to change the human relationship with the physical environment and our exposure to pathogens well into this one. Parasitic infections are also influenced by the new context in which hosts and vectors encounter pathogens. An urban renewal program in Brazil has provided a unique opportunity to compare the effect of antiparasitic treatment versus structural changes in the landscape of an urban community. Following treatment for *S. mansoni* infection in one section of the neighbourhood of São Bartolomeu, there was an increase in adequate sewer coverage from 68% to 92%, a decrease in flooded households from 49% to 31% and an apparent increase in disposable income across the neighbourhood. Within the same neighbourhood, a cohort of residents and parasites experienced the effects of both interventions (2011C), while another was only exposed to improvements in living conditions (2015C). While both groups had a higher prevalence of *S. mansoni* infection than the state average (4.1%, Brazilian Ministry of Health, 2018. http://www.saude.gov.br/boletins-epidemiologicos) the 4 years without community-wide treatment has not seen a return to 2011 levels. The treated and untreated cohorts do not appear very different demographically, socioeconomically (Table 2) or geographically (Fig. 4). The overall shift in age-specific prevalence by 4–5 years for the whole community further supports the importance of the improvements in living conditions. Thus, changes in the overall parasite population likely owe more to the changes in living conditions than a single round of praziquantel treatment for a section of the community.

During a 7 year period from 2004 to 2011 we reported little differentiation (Jost's d = 0.007) between parasite populations in São Bartolomeu (Blanton et al., 2015). The marked differentiation (Jost's d = 0.301) between 2011 and 2015 begs explanation. This large differentiation is not likely the result of immigration. While this has been a big factor in the rapid urbanisation of Salvador, immigration rates appear to be reaching a plateau as rural populations decline (Perz, 2000). Brazil in general is more urbanised than the US. In our study, we were able to identify only 23 immigrants arriving in the last 5 years, and only one of these was infected. This was also reflected in the overall risk being lower in those with a smaller percentage of their lifetime spent in the city.

It is possible that within the small area of a neighbourhood there are multiple well differentiated subpopulations of schistosomes, and that one or a few key sources were eliminated between 2011 and 2015, leaving the other well-differentiated subpopulations. This might explain the differentiation between 2011C and 2015C parasites, but such a scenario seems unlikely and is not consistent with our experience in other communities. A more likely source of this differentiation is genetic drift due to a bottleneck. One interpretation of Ne is as a measure of the susceptibility of the population to genetic drift. The São Bartolomeu population of *S. mansoni* experienced a steep decline in Ne over the four years between sampling. This reduced the likelihood of individuals acquiring the same parasite genotypes and resulted in increased differentiation between infrapopulations. The population was, therefore, more susceptible to drift, and this most likely accounts for the change in overall genetic population structure.

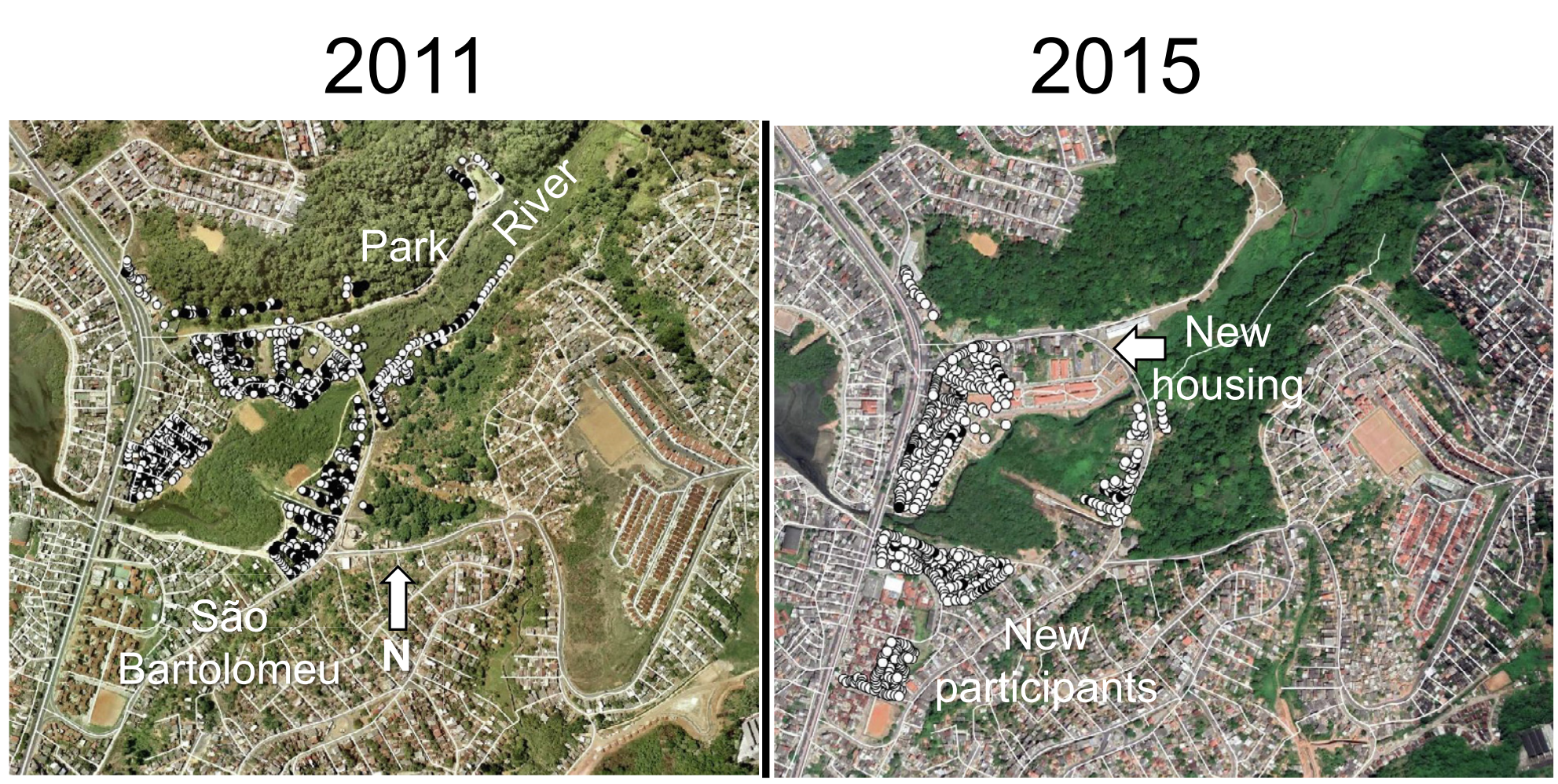

Fig. 4. Distribution of households in São Bartolomeu, Salvador, Brazil, with and without a *Schistosoma mansoni*-infected member. Houses were georeferenced using a handheld geo positioning device or Google Earth. Maps were produced using qGIS v. 2.18.15 QGIS Development Team, 2015. QGIS Geographic Information System (https://qgis.org/en/site/). Black circles mark households with at least one infected member. White circles mark households with no infected members.

Parasites from both cohorts demonstrate the same degree of marked differentiation from the 2011 population and are similarly close to the 2015 component population. Parasite populations, however, do demonstrate moderate differentiation between the cohorts in 2015 despite overlapping areas of residence within a few city blocks. For multiple historical reasons, the two cohorts are not entirely comparable. Those first seen in 2011 (2011C) were originally selected for residing in a part of the community that appeared to have a higher prevalence of disease, and while reducing their prevalence by more than two-fold, they continued to have a higher prevalence even post-treatment and changes in living conditions. By all other measures, however, the cohorts were equivalent (Table 2), until only those infected were compared (Table 4). Those who were part of the original cohort and became reinfected were younger, were more likely born in Salvador, less likely to have lived outside Salvador, but more likely to have travelled in the last year. The age and nature of exposure to Salvador itself were the major differences between the cohorts for those who were or became infected, and might be responsible for a differential acquisition of parasite genotypes. In addition, the 2011C cohort acquired their 2015 parasites over a period of 4 years, while the 2015C cohort may have acquired theirs over decades. While drift may explain the differentiation seen over time, there also appear to be differences in the behaviour of those infected that resulted in differentiation in these subpopulations.

In terms of other limitations, we further note that differences in the magnitude of statistical effect size might not directly translate to differences in functional effect. They indicated the strength of the association, but are not necessarily causative. Some samples could not be genotyped for some markers, however, the sensitivity analysis indicated that no single marker was skewing the results. In an unpublished study (unpublished data), we found that 10–15 markers produced indices similar to the use of 25 markers, so the absence of a few markers should not bias the results.

In a previous study (Blanton et al., 2015), we found no component population differentiation over a 7 year period in São Bartolomeu when there was no intervention. In rural Bahia, where all infected residents in the community were treated, little differentiation was observed 3 years after the first round of treatment, while Ne fell by more than 10 fold (Blanton et al., 2011). The marked differentiation observed, coupled with the 5 year shift in age-specific prevalence, strongly suggest that this was a community-wide effect, most consistent with the improvements made in living conditions. Urban living clearly has certain advantages that today increasingly pull rural populations into cities. While the ability to effect environmental changes for the health of a large number of individuals is probably not a major consideration for most immigrants to the cities, it is one of the potential benefits that public health agencies can take advantage of when working in conjunction with city planners and government.

Acknowledgements

This work was supported by National Institutes of Health (NIH, USA) grants R01 AI069195 and R01 AI121330. The Salvador Municipal Secretary of Health (Brazil) and the Fiocruz-Bahia (Brazil) contributed materials, transportation and personnel for the completion of this work.

Appendix A. Supplementary data

Supplementary data to this article can be found online at https://doi.org/10.1016/j.ijpara.2019.10.003.

References

Arntzen, J.W., Wallis, G.P., 2006. Restricted gene flow in a moving hybrid zone of the newts *Triturus cristatus* and *T. marmoratus* in Western France. Evolution (NY) 45, 805. https://doi.org/10.2307/2409691.

Ausubel, F.M., 1987. Current Protocols in Molecular Biology. Greene Pub. Associates, Hoboken, New Jersey.

Barbosa, C.S., Gonçalves, J.F., Albuquerque, Y., Barbosa, F.S., 1998. Urban schistosomiasis in Itamaracá Island, Pernambuco, Brazil: epidemiological factors involved in the recent endemic process. Mem. Inst. Oswaldo Cruz 93 (Suppl 1), 265–266. https://doi.org/10.1590/S0074-02761998000700049.

Barbosa, C.S., Leal-Neto, O.B., Gomes, E.C.S., de Araújo, K.C.G.M., Domingues, A.L.C., 2011. The endemisation of schistosomiasis in Porto de Galinhas, Pernambuco, Brazil, 10 years after the first epidemic outbreak. Mem. Inst. Oswaldo Cruz 106, 878–883. https://doi.org/10.1590/S0074-02762011000700014.

Barreto, A., 1960. [Schistosomiasis mansoni in the City of Salvador, Bahia. Studies on the Vector, Host/Parasite Relationships and Epidemiological Aspects] Esquistossomose mansônica na Cidade do Salvador. Estudo do vetor, relações parasito-hospedeiro e aspectos epi. Bol. Fund G Moniz 16, 1–80.

Blanton, R.E., Barbosa, L.M., Reis, E.A., Carmo, T.M., dos Santos, C.R.A., Costa, J.M., Aminu, P.T., Blank, W.A., Reis, R.B., Guimarães, I.C., Silva, L.K., Reis, M.G., 2015. The relative contribution of immigration or local increase for persistence of urban schistosomiasis in Salvador, Bahia, Brazil. PLoS Negl. Trop. Dis. 9. https://doi.org/10.1371/journal.pntd.0003521.

Blanton, R.E., Blank, W.A., Costa, J.M., Carmo, T.M., Reis, E.A., Silva, L.K., Barbosa, L.M., Test, M.R., Reis, M.G., 2011. *Schistosoma mansoni* population structure and persistence after praziquantel treatment in two villages of Bahia, Brazil. Int. J. Parasitol. 41, 1093–1099. https://doi.org/10.1016/j.ijpara.2011.06.002.

Brooker, S., Alexander, N., Geiger, S., Moyeed, R.A., Stander, J., Fleming, F., Hotez, P.J., Correa-Oliveira, R., Bethony, J., 2006. Contrasting patterns in the small-scale heterogeneity of human helminth infections in urban and rural environments in Brazil. Int J Parasitol 36, 1143–1151 [pii]10.1016/j.ijpara.2006.05.009.

Calasans, T.A.S., Souza, G.T.R., Melo, C.M., Madi, R.R., de Lourdes Sierpe Jeraldo, V., 2018. Socioenvironmental factors associated with *Schistosoma mansoni* infection and intermediate hosts in an urban area of northeastern Brazil. PLoS One. https://doi.org/10.1371/journal.pone.0195519.

Coura-Filho, P., 2005. Distribuição da esquistossomose no espaço urbano. 1. O caso da região metropolitana de Belo Horizonte, Minas Gerais, Brasil. Cad. Saude Publica 13, 245–255. https://doi.org/10.1590/s0102-311x1997000200014.

Dabo, A., Diarra, A.Z., Machault, V., Touré, O., Niambélé, D.S., Kanté, A., Ongoiba, A., Doumbo, O., 2015. Urban schistosomiasis and associated determinant factors among school children in Bamako, Mali, West Africa. Infect. Dis. Poverty 4. https://doi.org/10.1186/2049-9957-4-4.

Ernould, J.C., Kaman, A., Labbo, R., Couret, D., Chippaux, J.P., 2000. Recent urban growth and urinary schistosomiasis in Niamey, Niger. Trop Med. Int. Heal. (5), 431–437 https://doi.org/tmi577 [pii].

Guimaraes, C.T., de Souza, C.P., Carvalho Odos, S., Katz, N., 1993. Urban focus of schistosomiasis in a metropolitan area in a southeastern region of Brazil. Rev. Saude Publica 27, 210–213.

Guimaraes, I.C., Tavares-Neto, J., 2006. Urban transmission of schistosomiasis in children from a neighborhood of Salvador, Bahia. Rev Soc Bras Med Trop 39, 451–455. https://doi.org/S0037-86822006000500006 [pii].

Harris, P.A., Taylor, R., Thielke, R., Payne, J., Gonzalez, N., Conde, J.G., 2009. Research Electronic Data Capture (REDCap)-A metadata-driven methodology and workflow process for providing translational research informatics support. J. Biomed. Inf. 42, 377–381. https://doi.org/10.1016/j.jbi.2008.08.010.

Jost, L., 2008. GST and its relatives do not measure differentiation. Mol. Ecol. 17, 4015–4026. https://doi.org/10.1111/j.1365-294X.2008.03887.x.

Matthys, B., Koudou, B.G., N'Goran, E.K., Vounatsou, P., Gosoniu, L., Koné, M., Gissé, G., Utzinger, J., 2010. Spatial dispersion and characterisation of mosquito breeding habitats in urban vegetable-production areas of Abidjan, Côte d'Ivoire. Ann. Trop. Med. Parasitol. 104, 649–666. https://doi.org/10.1179/136485910x12851868780108.

Mwakitalu, M.E., Malecela, M.N., Mosha, F.W., Simonsen, P.E., 2014. Urban schistosomiasis and soil transmitted helminthiases in young school children in Dar es Salaam and Tanga, Tanzania, after a decade of anthelminthic intervention. Acta Trop. 133, 35–41. https://doi.org/10.1016/j.actatropica.2014.01.012.

Oliveira, D.S., Santos, V.B., Melo, A.G.S., Lima, Á.S., Carvalho, C.D., Allegretti, S.M., de Melo, C.M., Madi, R.R., Jeraldo, R.R., de Lourdes Sierpe Jeraldo, V., 2013. Schistosomiasis mansoni in urban Northeast Brazil: Influence of rainfall regime on the population dynamics of *Biomphalaria* sp. Rev. Soc. Bras. Med. Trop. 46, 654–657. https://doi.org/10.1590/0037-8682-1486-2013.

Perz, S.G., 2000. The rural exodus in the context of economic crisis, globalization and reform in Brazil. Int. Migr. Rev. 34, 842–881. https://doi.org/10.2307/2675947.

Pinto, H.A., Mati, V.L.T., de Melo, A.L., 2013. The Pampulha reservoir remains a potential urban focus of schistosomiasis mansoni in Brazil: Changes in the occurrence patterns of *Biomphalaria* species and a new record of the parasite. Rev. Soc. Bras. Med. Trop. 46, 478–483. https://doi.org/10.1590/0037-8682-0099-2013.

Sarda, R.K., Simonsen, P.E., Mahikwano, L.F., 1985. Urban transmission of urinary schistosomiasis in Dar es Salaam, Tanzania. Acta Trop. 42, 71–78.

Shen, G., Li, Q., La, D., Zhang, S., 1997. Special features of acute infection due to *Schistosoma japonicum* in urban and rural areas in China. Southeast Asian J. Trop. Med. Public Heal. 28, 280–284.

United Nations, 2015. World Urbanization Prospects: The 2014 Revision. United Nations, New York.

United Nations, 2013. World Economic & Social Survey: Sustainable Development Challenges. United Nations, New York.

World Bank. Global Monitoring Report 2013, 2013. Rural-Urban Dynamics and the Millennium Development Goals, Washington, DC: World Bank. The World Bank and the International Monetary Fund, Washington, DC.

Check for updates

Received: 3 March 2022 | Revised: 14 August 2022 | Accepted: 29 August 2022

DOI: 10.1111/tbed.14692

Transboundary and Emerging Diseases WILEY

RAPID COMMUNICATION

Evidence for local transmission and maintenance of schistosomiasis in an urban neighbourhood in Northeast Brazil

Camila F. Chaves[1] | Gilberto Sabino-Santos[2,3] | Fernanda Mac-Allister Cedraz[1] | Pedro Santos-Muccillo[1] | João Ricardo Filho[1] | Vanessa S. Zanardi[1] | Vanessa T. Moretto[1] | Adriano P. C. Santos[1] | Fabiano Simões[1,4,*] | Lucio M. Barbosa[1,5] | Luciano K. Silva[1] | Mitermayer G. Reis[1,6,7] | Ronald E. Blanton[2]

[1]Gonçalo Moniz Research Centre, Oswaldo Cruz Foundation, Salvador, Brazil

[2]Department of Tropical Medicine, Tulane University School of Public Health and Tropical Medicine, New Orleans, Louisiana, USA

[3]Centre for Virology Research, Ribeirao Preto Medical School, University of Sao Paulo, Ribeirao Preto, Brazil

[4]Centre for Zoonoses Control, Salvador Municipal Department of Health, R. Mocambo, S/N, Salvador, Bahia, Brazil

[5]Department of Biomedicine, Bahiana School of Medicine and Public Health, Salvador, Brazil

[6]School of Medicine, Federal University of Bahia, Salvador, Brazil

[7]Yale School of Public Health, Yale University, New Haven, Connecticut, USA

Correspondence
Ronald Blanton, Department of Tropical Medicine, Tulane University School of Public Health and Tropical Medicine, Tidewater Building, 1440 Canal Street, New Orleans, LA 70112, USA.
Email: rblanton1@tulane.edu

Funding information
NIH, Grant/Award Numbers: R01 AI121330, FAPESB-PNX0001/2015

Abstract

Schistosomiasis is a tropical neglected disease commonly associated with rural areas; however, urban schistosomiasis has been reported worldwide, and increasing urbanization is one of the most important demographic shifts of the 20th and now 21st centuries. The pattern of urbanization is not uniform so that within the same city the rates and sources of population increase vary. Here, we report on the parasite composition in one neighbourhood in the metropolitan area of Salvador, Bahia, Brazil. Using epidemiological data and population genetics, we find evidence for local transmission and maintenance of *Schistosoma mansoni* infection within an urban population and little contribution from rural–urban migration. Our findings provide direction for local mitigation strategies and to assist the public living in this neighbourhood to interrupt the local transmission cycle.

KEYWORDS
migration, population genetics, *Schistosoma mansoni*, urbanization

1 | INTRODUCTION

Since the 2000s, Brazil has seen a decrease in the rate of rural–urban migration; however, the urban population continues to grow and the rural population is decreasing (Alves & Marra, 2009; United Nations, 2018). The city of Salvador went through an urbanization process even more intense than the country as a whole (Souza et al., 2012). This rapid process produces areas with precarious housing and limited public utilities that put some communities at risk for transmission of parasites such as *Schistosoma mansoni*, the cause of schistosomiasis (Santana & Batista, 2012).

Schistosoma mansoni is the second most important parasitic infection after malaria for its prevalence and morbidity. It infects hundreds of millions in the Americas, Africa, Middle East and East Asia. Infection is commonly considered a disease of rural populations related to

Camila F. Chaves and Gilberto Sabino-Santos contributed equally to this work.

*In memoriam: to whom this work is dedicated.

 © 2022 Wiley-VCH GmbH.

agriculture, fishing, recreation and other activities associated with contact with freshwater (McManus et al., 2018). While urban disease is considered unusual, it has been well documented historically in Africa, China and Brazil (Blanton et al., 2015; Klohe et al., 2021). In Brazil, cities like Salvador, Bahia, in the Northeast have historically seen transmission of *S. mansoni*. Pirajá da Silva first distinguished *S. mansoni* from the species *S. haematobium* based on human cases in the city in 1911 (Katz, 2008). The main conditions for active transmission of *S. mansoni* are prevalent in some of the poorer sections of cities, that is infected humans, contact with surface waters (agricultural work, recreation), presence of susceptible snails and poor sanitation (Klohe et al., 2021; Zanardi et al., 2019). Measures to eliminate schistosomiasis as a public health threat are aided by understanding the presence and persistence of the infection in each location. Rural to urban immigration is likely to contribute, but it is important for public health planning to understand how much of a factor this is. The pattern of distribution is also key to the management of the infection. Transmission in urban centres is thought to be highly focal (McManus et al., 2018; Montgomery, 2020), but without knowledge of demography and parasite populations themselves this is difficult to verify.

Here using population genetics, we addressed for one urban community whether the presence of a population of *S. mansoni* resulted primarily from migration or local acquisition. We assessed place of birth and percentage of time living in a local neighbourhood designated Pirajá, in the city Salvador, Bahia in Northeast Brazil. We also evaluated genetic differences by gender, age and water contact points. Using epidemiologic and population genetic evidence, we show infection is present primarily due to local transmission in this section of a major Brazilian metropolis.

2 | MATERIALS AND METHODS

2.1 | Ethical approval

The study was approved by the Ethical Committee (CEP) of the Oswaldo Cruz Foundation, Bahia, the Brazilian Commission for Research Ethics (CONEP) under no. 33779414.7.0000.0040, and the Institutional Review Board (IRB) of Tulane University administrative review 2019-1799. All participants signed or marked informed consent forms. Participants under 18 years of age filled an assent form and their parents or guardians provided informed consent on their behalf.

2.2 | Study area and population survey

The Pirajá neighbourhood (12°53′45.00″ S 38°27′53.27″ W) (Figure 1) is within a densely populated area of the major city of Salvador, Bahia, Brazil whose population is nearly 3 million (CONDER, 2016). The community borders a large city park, São Bartolomeu, that formerly protected a river and reservoir that served as a source of drinking water for the city. Population pressure surrounding the park has meant the reservoir no longer serves this purpose but is an area for recreation and some small-scale agriculture (CONDER, 2016).

According to the 2010 census, Pirajá had a population of 33,341 inhabitants of which >50% are aged 20–49 years. Piped water is available to more than 95% of households and sewage is accessible to 91% (CONDER, 2016); however, many households are not connected to the system. Thirty per cent earn less than one minimum monthly salary (US$213) and 40% between one and three minimum salaries (CONDER, 2016). Proximity to bodies of water and especially water development activities have been identified as a risk factor for infection with schistosomes (Clennon et al., 2004; Kabuyaya et al., 2017; Mogeni et al., 2020). Therefore, for this study, a band of homes approximately two city blocks wide that borders the park, river and reservoir was selected for sampling.

The study area comprised 650 households where 2011 residents were interviewed and 1134 provided at least one stool sample for Kato–Katz assay. Interviews and stool sampling were performed as previously described with stools collected on three different days (Blanton et al., 2015) (Figure S1). Interview data were directly entered into a REDCap database (version 9.3.1-2021 Vanderbilt University) using Android-based tablets (Android version 9.1, Samsung Galaxy Tab A 8.0).

2.3 | Stool survey and egg isolation

All stool samples collected from participants were weighed, and single slides were prepared as previously described (Katz & Pellegrino, 1972). On the following day, the slides were read, and the number of schistosome eggs per gram and the presence of other helminths were recorded. Stools positive for *S. mansoni* were homogenized in a blender with 200 ml of 2% saline solution and then processed through a series of metal sieves and nylon filter bags (55–300 μm mesh pore size; FSI, Michigan City, Indiana, USA) and gravity sedimentation to concentrate the eggs (Dresden & Payne, 1981). The bottom 5 ml of sediment was collected and then kept frozen at −20°C for DNA extraction. In accordance with Brazilian Ministry of Health guidelines (Ministério da Saúde do Brasil, 2014), participants with one or more egg-positive stool samples were given a one-time dose of praziquantel (60 mg/kg for children 4–15 years old and 50 mg/kg for adults), and 4–6 weeks later three follow-up stool examinations were performed on those treated. For other helminthiases, a single dose of albendazole was provided (Figure S1).

2.4 | DNA extraction and genotyping

DNA was isolated from the sediment by a standard phenol/chloroform protocol, and then treated with cetyltrimethylammonium bromide to remove PCR inhibitors as previously described (Ausubel, 1987;

18651682, 2022, 6, Downloaded from https://onlinelibrary.wiley.com/doi/10.1111/tbed.14692 by Tulane University Hml, Wiley Online Library on [25/07/2025]. See the Terms and Conditions (https://onlinelibrary.wiley.com/terms-and-conditions) on Wiley Online Library for rules of use; OA articles are governed by the applicable Creative Commons License

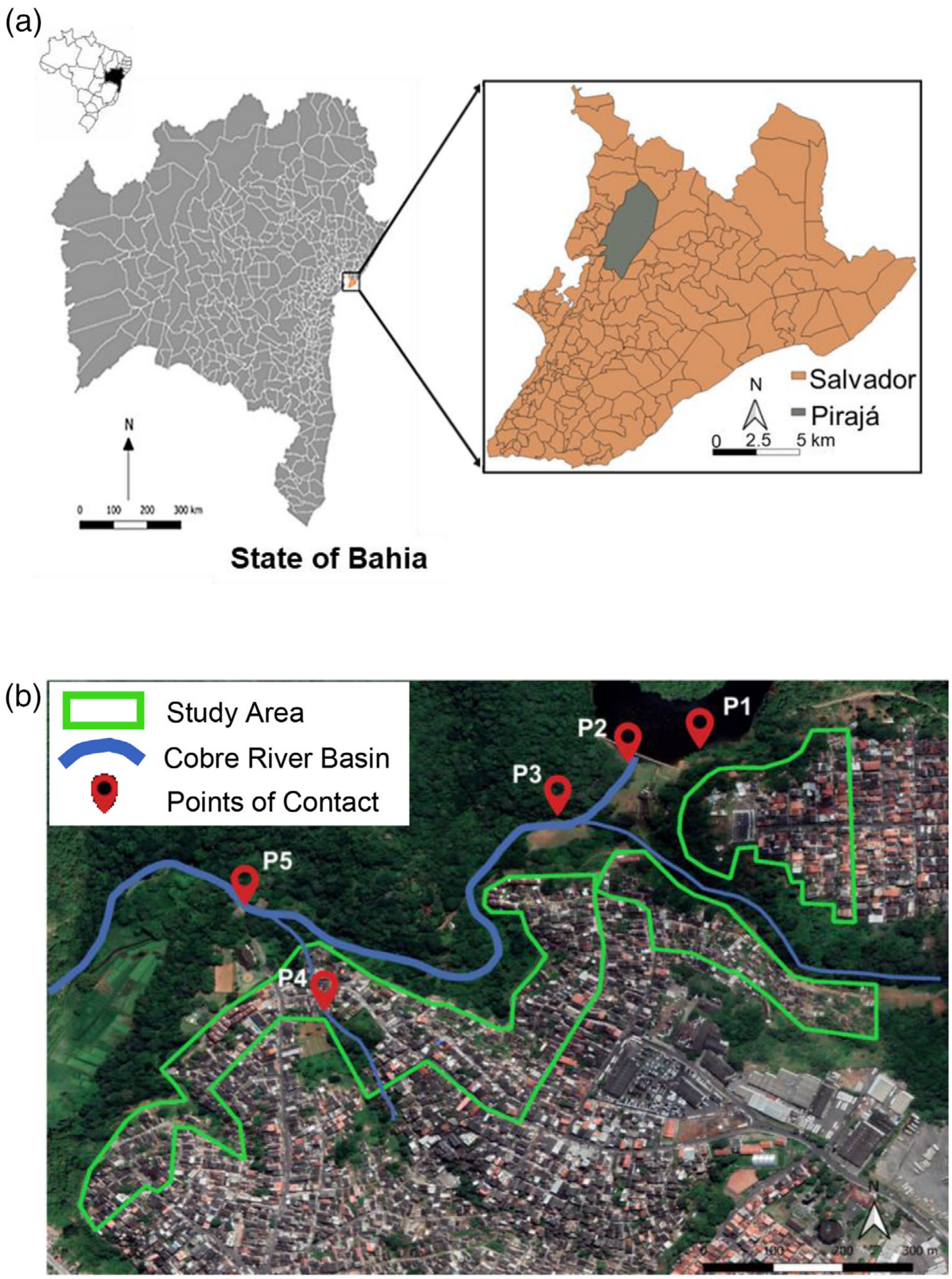

FIGURE 1 Study area: Pirajá neighbourhood in Salvador city, State of Bahia, north-eastern Brazil. (a) Pirajá (black) is shown within the municipal area of Salvador, Bahia, Brazil. (b) Study area and distribution of points along the Cobre River basin where the community in Pirajá often gets in contact with water: (P1) Represa do Cobre, (P2) Barragem Sete Quedas, (P3) Córrego do Campo, (P4) Vala da Baixa da Fonte, and (P5) Cachoeira de Nanã. This map was generated using QGIS version 3.18.3 (Zürich-CH)

Blanton et al., 2011). For genotyping, duplicate 2 μl of DNA samples per primer pair was used to PCR amplify 15 polymorphic microsatellite loci (Table S1) with a SeqStudio-3200 Genetic Analyzer (Thermo Fisher, Carlsbad, CA, USA). Allele peaks were identified and measured using Peak Scanner software version 2.0 online workstation (Thermo Fisher, Carlsbad, CA, USA), and the data were transferred to an Excel template for processing. Data trimming and organization were automated by custom-designed macros. Allele peaks not conforming to the stepwise mutation model and peaks <100 pixels in height were excluded from analysis. Allele frequencies were calculated based on the ratio between each allele peak's height and the sum of allele peak heights for each microsatellite locus (Barbosa et al., 2016). Microsatellite loci and samples with less than 50% genotyping success were excluded from analyses (Table S1). Final analyses were performed for 51 infrapopulations (parasites aggregated within one host) and 15 loci (smms2, smms13, smms16, smms3, smms17, smms18, smms21, smda23, sm13-478, 1f8a, 29e6a, smu31768, lg3_sc36b, sc23b, and smd28) (Kovach et al., 2021).

18651682, 2022, 6, Downloaded from https://onlinelibrary.wiley.com/doi/10.1111/tbed.14692 by Tulane University Hml, Wiley Online Library on [25/07/2025]. See the Terms and Conditions (https://onlinelibrary.wiley.com/terms-and-conditions) on Wiley Online Library for rules of use; OA articles are governed by the applicable Creative Commons License

TABLE 1 *Schistosoma mansoni* infection associations for the participating population in the Pirajá neighbourhood in Salvador, Bahia, Brazil ($n = 1134$)

Traits	Crude OR[a]	95% CI	Adjusted OR[b]	95% CI
**Male sex**	**3.0**	**1.7–5.1**	**3.0**	**1.7–5.4**
**>20 years old**	**2.4**	**1.2–4.7**	**3.5**	**1.7–7.3**
Immigrants	0.7	0.3–2.1	–	–
**Immigrant percent lifetime in Salvador**	–	–	**1.6**	**1.2–2.0**
**History of travelling**	**0.4**	**0.1–0.9**	**0.3**	**0.1–0.7**
Water contact—Traveling	1.9	0.3–11.4	–	–
SES D/E vs. B/C	1.4	0.9–2.4	–	–
Adequate Sewage	0.6	0.1–4.9	–	–
**Water contact—Pirajá**	**3.4**	**1.9–6.0**	**2.7**	**1.0–6.8**
**P1: Represa do Cobre**	**2.8**	**1.7–4.8**	**2.2**	**1.2–3.9**
**P2: Barragem Sete Quedas**	**3.5**	**2.0–6.0**	**2.2**	**1.2–4.0**
P3: Córrego do Campo	2.2	1.2–4.2	1.3	0.6–2.7
P4: Vala da Baixa da Fonte	1.7	0.4–7.3	–	–
**P5: Cachoeira de Nanã**	**8.6**	**3.6–20.8**	**6.9**	**2.7–17.8**
Types of contact				
Leisure	2.6	1.5–4.3	0.8	0.4–2.0
While walking	3.2	1.8–5.5	1.3	0.6–2.8
Doing laundry	4.5	1.2–16.4	2.9	0.7–12.6
Fishing	3.2	1.8–6.0	0.9	0.4–1.9
Work	2.1	0.5–9.2	–	–
Frequency >7 times a week	3.3	1.9–5.8	1.3	0.2–2.6
Duration >1 h	3.3	1.9–5.9	1.5	0.7–3.1

Note: En dash '–' indicates value was undefined or there were cells equal to zero. The effect is in logistic scale and OR was calculated as $e^{\text{coefficient}}$ for each variable in the model. Statistically significant results based on the adjusted OR are presented in bold print. The crude OR is based on univariable analysis of bivariable categories against the dependent variables: infection or no infection with *Schistosoma mansoni*. Percent lifetime spent in Salvador, however, is a continuous independent variable assessed for those not born in Salvador. History of traveling was positive if the respondent had left the city of Salvador in the last year. Water contact while traveling was also asked. P = Water contact point. SES D/E vs. B/C: socio-economic status in Brazil is categorized by letters. The most disadvantaged (D and E) grouped here in one category and those with better conditions (B and C) forming another group. There were no participants in the A category. Adequate sewage was a connection to the municipal system or septic tank.

[a]OR calculated by cross product.

[b]Logistic regression including all variables significant in univariate analysis.

2.5 | Genetic differentiation

The differentiation index, Jost's *D* (Jost, 2008), was calculated from the allele frequencies using SpadeR (Chao & Jost, 2015). Where Jost's *D* between replicates was >0.01, the replicates were re-examined or eliminated (Silva et al., 2020). Differentiation of <0.05 was considered little differentiation, 0.05–0.25 moderate or great differentiation and >0.25 as very great differentiation (Cormack et al., 1990).

The average differentiation among pairwise infra-populations (parasite aggregated within one host) is designated Di, between component populations Dc (parasites aggregated within a group of hosts) and between an infra-population and a component population is Dic. Genetic differences were analysed among component populations of *S. mansoni* based on the following traits: age, sex, place of birth, percentage of time living in Piraja, history of traveling outside of Piraja and contact with freshwater during traveling and contact with freshwater at five points within Piraja (P1: Represa do Cobre; P2: Barrage Sete Quedas; P3: córrego do campo; P4: vala da baixa da fonte; P5: Cachoeira de Nanã). Principal component analysis of Dic was used to identify clustering based on the selected traits (Freeman & Jackson, 1992). To assess genetic relationship based on Dic, we also conducted a network analysis (Kivelä et al., 2015). Principal component and network analyses were performed with an open-source statistical package PAST version 4.03 (Hammer et al., 2001).

2.6 | Data analysis

Univariate comparisons between *S. mansoni* infection and categorical variables were performed by a chi-square test with Yates' correction or Fisher's exact test when appropriate, and continuous variables were examined using Kruskal–Wallis test. The crude odds ratio (OR)

18651682, 2022, 6, Downloaded from https://onlinelibrary.wiley.com/doi/10.1111/tbed.14692 by Tulane University Hml, Wiley Online Library on [25/07/2025]. See the Terms and Conditions (https://onlinelibrary.wiley.com/terms-and-conditions) on Wiley Online Library for rules of use; OA articles are governed by the applicable Creative Commons License

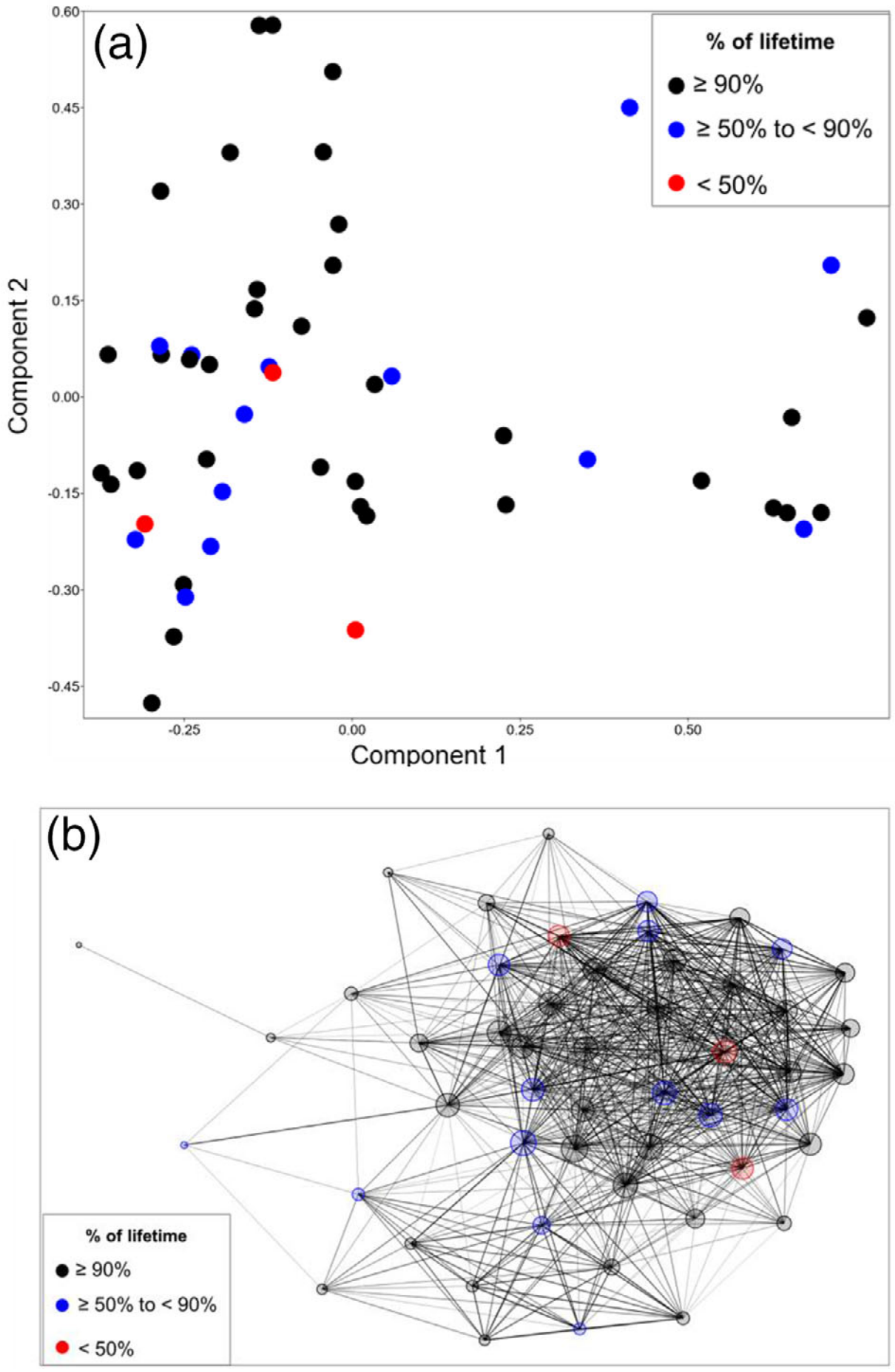

FIGURE 2 Genetic differentiation for *Schistosoma mansoni*: percentage (%) of lifetime between 51 infra-populations and the component population of parasites of the native-borne (Dic) in Pirajá neighbourhood, Salvador-BA, Brazil, 2019, using 15 microsatellite loci (smms2, smms13, smms16, smms3, smms17, smms18, smms21, smda23, sm13-478, 1f8a, 29e6a, smu31768, lg3_sc36b, sc23b, and smd28). (a) Principal component analysis comparing genetic distances for the percentage of lifetime in Salvador-BA, Brazil. Support values were calculated by bootstraps with 1000 iterations. The first two principal components were graphed. Component 1: eigenvalue = 0.112, 39% of variance; Component 2: eigenvalue = 0.059, 21% of variance. (b) Network array comparing genetic distances for percentage of lifetime in Salvador-BA, Brazil. Network nodes represent individuals from the infra-population. Links represent genetic distances. Larger nodes mean more interaction and thicker lines more similarities. For all analyses, genetic distances using Dic were applied as variance–covariance matrix

and 95% confidence interval (CI) were obtained as a measure of the strength of association with the corresponding predictor and schistosomiasis. Adjusted OR was obtained from multivariate logistic regression analysis with the corresponding predictor and *S. mansoni* infection. All descriptive, univariate, and multivariate analyses were performed in Epi Info 7.2.2.6 (https://www.cdc.gov/epiinfo/index.html). We also performed Student's *t* test to evaluate differences between Dics from immigrants and natives and Mann–Whitney to assess differences between Dics for percent of lifetime spent in Salvador.

3 | RESULTS AND DISCUSSION

Of 2011 residents interviewed, 1134 (56%) provided at least one stool sample of which 62 were positive for *S. mansoni* (5.5%, 95% CI: 4.2%–7.0%; Table S2) and 75 for geohelminths (6.6%). Thus, schistosomiasis in the evaluated group living in Pirajá is five times higher than the national average of 1% and more than twice as high as the Bahia state average of 2.1% (Katz, 2018; Ministério da Saúde do Brasil, 2014). This is typical of many rural areas of the state. There was an association between *S. mansoni* infection and male sex (adjusted OR: 3.0, 95% CI: 1.7–5.4) and age >20 years old (adjusted OR: 3.5, 95% CI: 1.7–7.3; Table 1; Figure S2). This is consistent with what we have observed in other urban areas of Salvador (Barbosa et al., 2016; Blanton et al., 2015; Silva et al., 2020) and in urban infection in Pernambuco State (Gomes et al., 2022). However, it contrasts with the younger age-specific risk of infection in rural areas (Blanton, 2019; Klohe et al., 2021) and with an urban area of Sergipe (Calasans et al., 2018). For those infected, the mean intensity was 89 ± SD 172 eggs per gram of faeces (epg), low by WHO criteria (WHO Expert Committee, 2002; World Health Organization, 2019) (Table S2). This was similar to two recent studies of urban schistosomiasis in Brazil (Calasans et al., 2018; Gomes et al., 2022).

Specific local risk was assessed by reported contact with surface water and association with *S. mansoni* infection. We observed an overall risk with water contact (adjusted OR: 2.7, 95% CI: 1.0–6.8), but the risk was specifically associated with points P1 (adjusted OR: 2.2, 95% CI: 1.2–3.9), P2 (adjusted OR: 2.2, 95% CI: 1.2–4.0) and P5 (adjusted OR: 6.9, 95% CI: 2.7–17.8) (Table 1). At all of the associated sites, residents tended to have full body contact or emersion. The adjusted OR was not significant for P3 (Corrego do Campo) and P4 (Vala da Baixa da Fonte). The Córrego is a small stream that courses along a soccer field, and residents only have occasional contact. The Vala is the site where an underground spring surfaces and has little faecal contamination or frequent contact by the population.

Migration has been indicated as a risk factor for urban schistosomiasis (Blanton, 2019; Klohe et al., 2021), but being born in Salvador was neither protective nor a risk for infection. Immigrants (those not born in Salvador) were 17% of the sample (Table S2) and were no more likely to be infected than the native born (OR: 1.98, 95% CI: 0.84–4.67) (Table 1). Spending a greater percent of lifetime in Salvador for immigrants was associated with infection (adjusted OR: 1.6, 95% CI: 1.2–2.0). Distribution of schistosomiasis by age revealed higher risk among male young adults and higher parasitic load among those above 60 years old (Figure S2). Mean percentage of lifetime in Salvador was associated with *S. mansoni* or infection intensity (Table 1). While intensity of infection was numerically higher in immigrants (141 vs. 83 eggs per gram, respectively), this was not statistically significant (p = .65). The higher intensity of infection in the 61–70 and 70+ age groups (represented by two and one individual, respectively) is likely due to the small numbers. A history of traveling away from Salvador was protective (adjusted OR: 0.3, 95% CI: 0.1–0.7) (Table 1). These data suggest that immigration was not associated with risk for schistosomiasis, and local acquisition was more

18651682, 2022, 6, Downloaded from https://onlinelibrary.wiley.com/doi/10.1111/tbed.14692 by Tulane University Hml, Wiley Online Library on [25/07/2025]. See the Terms and Conditions (https://onlinelibrary.wiley.com/terms-and-conditions) on Wiley Online Library for rules of use; OA articles are governed by the applicable Creative Commons License

important. Indeed, the more the time spent in the city, the greater the risk of infection.

Analysis of genetic differentiation of parasite populations further supports primarily a local transmission pattern. Of the 62 stool samples collected, 51 were successfully genotyped (80%). The average differentiation between infra-populations (Di) was 0.22 (Table S1) and the differentiation between infra-populations from immigrants compared to the native-born (Dc) was 0.06. While the average Di indicates that individuals were acquiring genetically different groups of parasites, this is typical of areas with low infection intensities. Dc between parasites of immigrants and parasites of native-born was low relative to Di, indicating that the two groups were drawn from the same source.

The genetic distance of individuals from the group is another approach to evaluating whether parasites of immigrants largely originated outside of the Pirajá community. We have shown that communities separated by 6 km on the same river can be distinguished by Dc (Blanton et al., 2011) and principal coordinate with k-means analysis of Dic or Nei's genetic distance (Long et al., 2022). Using parasites of those born in Salvador as the component population, no clustering was observed by principal component or network analyses (Figure S3). We also stratified by percentage of lifetime spent in Salvador and no clustering was observed as well (Figure 2). No clustering was observed by principal component analyses when comparing differences by age, sex and history of contact with freshwater bodies when traveling and at the distinct points in Pirajá neighbourhood (Figure S4).

It is often unclear if the problem of schistosomiasis is rising or falling with increasing urbanization or if rural–urban migration is fuelling the presence and persistence of the infection in cities (Klohe et al., 2021). In Pirajá, immigrants may be more sinned against than sinning (King Lear, 3.2 49–60, Shakespeare) as far as schistosomiasis is concerned. Although some importation of S. *mansoni* is possible due to immigration, even necessary to establish local transmission, the evidence in this urban neighbourhood suggests that immigrants primarily become infected in the city. The conditions of crowding and sanitation perpetuate transmission and focusing on these conditions will powerfully resolve the issue of urban schistosomiasis.

AUTHOR CONTRIBUTIONS

Camila F. Chaves, Lucio M. Barbosa, Luciano K. Silva, Mitermayer G. Reis and Ronald E. Blanton conceived and designed the study. Camila F. Chaves, Gilberto Sabino-Santos, Fernanda Mac-Allister Cedraz, Pedro Santos-Muccillo, Vanessa S. Zanardi, Vanessa T. Moretto, Adriano P. C. Santos, Fabiano Simões and Lucio M. Barbosa were involved in acquisition of data. Camila F. Chaves, Gilberto Sabino-Santos, Fernanda Mac-Allister Cedraz, Lucio M. Barbosa, Luciano K. Silva, Mitermayer G. Reis, and Ronald E. Blanton analysed and interpreted of data. Camila F. Chaves, Gilberto Sabino-Santos and Ronald E. Blanton drafted the original version of the manuscript. Camila F. Chaves, Gilberto Sabino-Santos, Fernanda Mac-Allister Cedraz, Lucio M. Barbosa, Luciano K. Silva and Ronald E. Blanton were involved in reviewing and editing. Mitermayer G. Reis and REB contributed with reagents/material and analysed tools. All authors contributed for the final version of the manuscript.

ACKNOWLEDGEMENTS

We thank Claúdio Andrade for all the support in fieldwork and we have enormous gratitude to the Pirajá community. This work was supported by NIH R01 AI121330 and FAPESB-PNX0001/2015. We also thank the Salvador Municipal Secretary of Health, the Bahiana School of Medicine and Public Health and the Fiocruz-Bahia who contributed space, materials, transportation and personnel for the completion of this work.

CONFLICT OF INTEREST

The authors declare no conflict of interest.

DATA AVAILABILITY STATEMENT

The data that support the findings of this study are available from the corresponding author upon reasonable request

ORCID

Ronald E. Blanton https://orcid.org/0000-0001-6655-7336

REFERENCES

Alves, E., & Marra, R. (2009). A persistente migração rural-urbana. *Revista de Política Agrícola*, *18*, 5–17.

Ausubel, F. (1987). *Current protocols in molecular biology*. Greene Pub. Associates.

Barbosa, L. M., Reis, E. A., dos Santos, C. R. A., Costa, J. M., Carmo, T. M., Aminu, P. T., Pitanga, T. N., Ponce-Terashima, R., Blank, W. A., Silva, L. K., Reis, M. G., & Blanton, R. E. (2016). Repeated praziquantel treatments remodel the genetic and spatial landscape of schistosomiasis risk and transmission. *International Journal for Parasitology*, *46*, 343–350. https://doi.org/10.1016/j.ijpara.2016.01.007

Blanton, R. E. (2019). Population structure and dynamics of helminthic infection: Schistosomiasis. *Microbiology Spectrum*, *7*(4). https://doi.org/10.1128/microbiolspec.ame-0009-2019

Blanton, R. E., Barbosa, L. M, Reis, E. A., Carmo, T. M., dos Santos, C. R. A., Costa, J. M., Aminu, P. T., Blank, W. A., Reis, R. B., Guimarães, I. C., Silva, L. K., & Reis, M. G. (2015). The relative contribution of immigration or local increase for persistence of urban schistosomiasis in Salvador, Bahia, Brazil. *PLoS Neglected Tropical Diseases*, *9*, 1–14. https://doi.org/10.1371/journal.pntd.0003521

Blanton, R. E., Blank, W. A., Costa, J. M., Carmo, T. M., Reis, E. A., Silva, L. K., Barbosa, L. M., Test, M. R., & Reis, M. G. (2011). *Schistosoma mansoni* population structure and persistence after praziquantel treatment in two villages of Bahia, Brazil. *International Journal for Parasitology*, *41*, 1093–1099. https://doi.org/10.1016/j.ijpara.2011.06.002

Calasans, T. A. S., Souza, G. T. R., Melo, C. M., Madi, R. R., & de Lourdes Sierpe Jeraldo, V. (2018). Socioenvironmental factors associated with *Schistosoma mansoni* infection and intermediate hosts in an urban area of northeastern Brazil. *Plos One*, *13*, 1–14. https://doi.org/10.1371/journal.pone.0195519

Chao, A., & Jost, L. (2015). Estimating diversity and entropy profiles via discovery rates of new species. *Methods in Ecology and Evolution*, *6*, 873–882. https://doi.org/10.1111/2041-210X.12349

Clennon, J. A., King, C. H., Muchiri, E. M., Kariuki, H. C., Ouma, J. H., Mungai, P., & Kitron, U. (2004). Spatial patterns of urinary schistosomiasis infection in a highly endemic area of coastal Kenya. *American Journal of Tropical Medicine and Hygiene*, *70*, 443–448. https://doi.org/10.4269/ajtmh.2004.70.443

CONDER. (2016). *Painel de Informações Dados Socioeconômicos Do Município de Salvador* (5th ed.). CONDER Informs.

18651682, 2022, 6, Downloaded from https://onlinelibrary.wiley.com/doi/10.1111/tbed.14692 by Tulane University Hml, Wiley Online Library on [25/07/2025]. See the Terms and Conditions (https://onlinelibrary.wiley.com/terms-and-conditions) on Wiley Online Library for rules of use; OA articles are governed by the applicable Creative Commons License

Cormack, R. M., Hartl, D. L., & Clark, A. G. (1990). Principles of population genetics. *Biometrics*, *46*, 546, https://doi.org/10.2307/2531471
Dresden, M. H., & Payne, D. C. (1981). A sieving method for the collection of schistosome eggs from mouse intestines. *Journal of Parasitology*, *67*, 450–452. https://doi.org/10.2307/3280573
Freeman, J., & Jackson, J. E. (1992). A user's guide to principal components. *Journal of the Operational Research Society*, *43*(6), 641–641.
Gomes, E. C. S., da Silva, I. E. P., do Nascimento, W. R. C., Loyo, R. M., Domingues, A. L. C., & Barbosa, C. S. (2022). Urban schistosomiasis: An ecological study describing a new challenge to the control of this neglected tropical disease. *Lancet Reg. Heal. - Am.*, *8*, 100144, https://doi.org/10.1016/j.lana.2021.100144
Hammer, Ø., Harper, D. A., & Ryan, P. D. (2001). Past paleontological statistics software package for education and data analysis Øyvind. *Palaeontol. Electron.*, *4*, 9.
Jost, L. (2008). GST and its relatives do not measure differentiation. *Molecular Ecology*, *17*, 4015–4026. https://doi.org/10.1111/j.1365-294X.2008.03887.x
Kabuyaya, M., Chimbari, M. J., Manyangadze, T., & Mukaratirwa, S. (2017). Schistosomiasis risk factors based on the infection status among school-going children in the Ndumo area, uMkhanyakude district, South Africa. *South. African J. Infect. Dis.*, *32*, 67–72. https://doi.org/10.1080/23120053.2016.1266139
Katz, N. (2008). The discovery of *Schistosomiasis mansoni* in Brazil. *Acta Tropica*, *108*, 69–71. https://doi.org/10.1016/j.actatropica.2008.05.002
Katz, N. (2018). *Inquérito Nacional de Prevalência Da Esquistossomose Mansoni e Geo-Helmintoses*. CPqRR.
Katz, N. A. C, & Pellegrino, J. (1972). A simple device for quantitative stool thick-smear technique in *Schistosomiasis mansoni*. *Revista Do Instituto De Medicina Tropical De Sao Paulo*, *14*, 397–400.
Kivelä, M., Arnaud-Haond, S., & Saramäki, J. (2015). EDENetworks: A user-friendly software to build and analyse networks in biogeography, ecology and population genetics. *Molecular Ecology Resources*, *15*, 117–122. https://doi.org/10.1111/1755-0998.12290
Klohe, K., Koudou, B. G., Fenwick, A., Fleming, F., Garba, A., Gouvras, A., Harding-Esch, E. M., Knopp, S., Molyneux, D., D'souza, S., Utzinger, J., Vounatsou, P., Waltz, J., Zhang, Y., & Rollinson, D. (2021). A systematic literature review of schistosomiasis in urban and peri-urban settings. *PLoS Negl. Trop. Dis.*, *15*, 1–19. https://doi.org/10.1371/journal.pntd.0008995
Kovach, J. D., Long, J. C., Barbosa, L. M., Moura, A. R. S. S., Silva, L. K., Reis, M. G., & Blanton, R. E. (2021). A *Schistosoma mansoni* tri- and tetramer microsatellite catalog for genetic population diversity and differentiation. *Int. J. Parasitol*, https://doi.org/10.1016/j.ijpara.2021.04.002
Long, J. C., Taylor, S. E., Barbosa, L. M., Silva, L. K., Reis, M. G., & Blanton, R. E. (2022). Cryptic population structure and transmission dynamics uncovered for *Schistosoma mansoni* populations by genetic analyses. *Science Reports*, *12*, 1–10. https://doi.org/10.1038/s41598-022-04776-0
McManus, D. P., Dunne, D. W., Sacko, M., Utzinger, J., Vennervald, B. J., & Zhou, X. N. (2018). Schistosomiasis. *Nat. Rev. Dis. Prim.*, *4*, 1–19. https://doi.org/10.1038/s41572-018-0013-8
Ministério da Saúde do Brasil (2014): *Vigilância Da Esquistossomose Mansoni-Diretrizes Técnicas* (4th ed.). Ministério da Saúde.
Mogeni, P., Vandormael, A., Cuadros, D., Appleton, C., & Tanser, F. (2020). Impact of community piped water coverage on re-infection with urogenital schistosomiasis in rural South Africa. *Elife* 9, https://doi.org/10.7554/eLife.54012
Montgomery, S. (2020). Schistosomiasis. In G. W. Brunette & J. B. Nemhauser (Eds.), *CDC Yellow Book 2020: Health information for international travel*. Centers for Disease Control and Prevention. https://wwwnc.cdc.gov/travel/yellowbook/2020/travel-related-infectious-diseases/schistosomiasis
Santana, A., & Batista, M. (2012). *Manual do Saneamento Básico*. Instituto Trata Brasil.
Silva, L. K., Barbosa, L. M., Kovach, J. D., dos Santos Teixeira, R., Soares, Ê. S., Cardoso, C. W., Marques, R. V. M., dos Santos, T. P., Reis, M. G., & Blanton, R. E. (2020). The changing profile of schistosomiasis in a changing urban landscape. *International Journal for Parasitology*, *50*, 27–34. https://doi.org/10.1016/j.ijpara.2019.10.003
Souza, S. S., Barbosa, L. M., Guimarães, I. C., Blank, W. A., Reis, R. B., Reis, M. G., Blanton, R. E., & Andrade, Z. A. (2012). Genetic population structure of cercariae from an urban foci of *Schistosoma mansoni*, Brazil. *American Journal of Tropical Medicine and Hygiene*, *87*, 843–849. https://doi.org/10.4269/ajtmh.2012.11-0641
United Nations. (2018). *World urbanization prospects 2018: Country profiles*. Author.
WHO Expert Committee. (2002). *Prevention and control of schistosomiasis and soil-transmitted helminthiasis* (Vol. 912). World Health Organization.
William, S. (1970). King Lear. In A. Harbridge (Ed.), *The Complete Pelican Shakespeare* (1st ed. revised, p. 1083). Baltimore, MD, Act 3, Scene 2.
World Health Organization. (2019). *Schistosomiasis: Key facts*. Author.
Zanardi, V. S., Barbosa, L. M., Simões, F. M., Thiengo, S. C., Blanton, R. E., Junior, G. R., Silva, L. K., & Dos Reis, M. G. (2019). Prevalence of Infection of *Biomphalaria glabrata* by *Schistosoma mansoni* and the risk of urban *Schistosomiasis mansoni* in Salvador, Bahia, Brazil. *Revista Da Sociedade Brasileira De Medicina Tropical*, *52*, e20190171. https://doi.org/10.1590/0037-8682-0171-2019

SUPPORTING INFORMATION

Additional supporting information can be found online in the Supporting Information section at the end of this article.

How to cite this article: Chaves, C. F., Sabino-Santos, G., Cedraz, F. M.-A., Santos-Muccillo, P., Filho, J. R., Zanardi, V. S., Moretto, V. T., Santos, A. P. C., Simões, F., Barbosa, L. M., Silva, L. K., Reis, M. G., & Blanton, R. E. (2022). Evidence for local transmission and maintenance of schistosomiasis in an urban neighbourhood in Northeast Brazil. *Transboundary and Emerging Diseases*, *69*, 3153–3159. https://doi.org/10.1111/tbed.14692

18651682, 2022, 6, Downloaded from https://onlinelibrary.wiley.com/doi/10.1111/tbed.14692 by Tulane University Hml, Wiley Online Library on [25/07/2025]. See the Terms and Conditions (https://onlinelibrary.wiley.com/terms-and-conditions) on Wiley Online Library for rules of use; OA articles are governed by the applicable Creative Commons License
