# Supplementary material for: Schistosomiasis as an urban disease: 13 years of studies in the neighborhoods of Salvador, BA, Brazil
Source: PLoS Negl Trop Dis. 2026 Oct 23;20(10):e0013418. doi: 10.1371/journal.pntd.0013418 (PMC13734582; doi:10.1371/journal.pntd.0013418)
Supplement: Supplementary file 2 [file pntd.0013418.s001.docx]

**S1 Table**

| Study Site | Abbreviations | Years | Initial Enrollment | Study Design |
| --- | --- | --- | --- | --- |
| São Bartolomeu | SB2011, SB2015, SB2024 | 2011, 2015, 2024 | 1050 | Pilot, longitudinal |
| Dique Do Cabrito | DC2017 | 2017 | 1253 | Pilot, cross-sectional |
| Instituto Anísio Teixeira | IAT | 2018 | - | Pilot |
| Amazonas | AMZ | 2018 | - | Pilot |
| Saramandaia | SA2018, SA2020 | 2018, 2020 | 1786 | Pilot, longitudinal |
| Pirajá | PI2019, PI2022 | 2019, 2022 | 1128 | Pilot, longitudinal |
| Lagoa da Paixão | LP2022 | 2022 | 1035 | Pilot, cross-sectional |
| Bate Facho | BF2023 | 2023 | 611 | Pilot, cross-sectional |

Site selection and study design for urban schistosomiasis surveys in Salvador, Brazil (2011–2024)
