# Supplementary material for: Schistosomiasis as an urban disease: 13 years of studies in the neighborhoods of Salvador, BA, Brazil
Source: PLoS Negl Trop Dis. 2026 Oct 23;20(10):e0013418. doi: 10.1371/journal.pntd.0013418 (PMC13734582; doi:10.1371/journal.pntd.0013418)
Supplement: Supplementary file 3 [file pntd.0013418.s002.docx]

**S1 Data**

a) São Bartolomeu 2011, 2015 and 2024 (SB2011, SB,2015 and SB2024): São Bartolomeu is located along the outflow of the Cobre Basin that drains to the All-Saints Bay. The community is adjacent to São Bartolomeu Park and the community of Pirajá. At some point before 2011, the Park area was invaded for the construction of illegal houses and the establishment of agricultural activities. In 2013 and 2018, the state of Bahia implemented urban renewal with new home construction and improved sanitation for part of the community through the Minha Casa, Minha Vida (MCMV) program and the park area has been redefined. Many residents were relocated to the Pirajá neighborhood (see below) and others returned to SB later, but residents did not participate in survey and treatment in 2015. The urban garden remains active until today and all the urban agricultural workers were treated recurrently by us in 2015, 2017 and 2024 due to reinfection with high parasitic loads.
b) Dique do Cabrito, 2017 (DC2017): Dique do Cabrito is a small artificial lake formed early in the 20^th^ century by damming a stream originating from an underground spring. This flowed into the Cobre Basin at the lake’s northern pole and into the Camarajipe Basin at its southern pole.
c) Saramandaia 2018, 2019 and 2023 (SA2018, SA2019 and SA2023): Saramandaia developed following the influx of workers to construct the nearby interstate bus terminal and the Department of Motor Vehicles in the 1970’s. The area is drained by small streams that flow into the Camarajipe Basin. It was home to one of the largest urban gardens in the city. In 2016, the urban agricultural workers in this location were the target of treatment by the Municipal Health Department, but not the adjacent community. *Biomphalaria* *glabrata*. shedding *S. mansoni* cercariae have been found in these waters (Zanardi et al., 2019a). In 2019, this community underwent environmental changes with the replacement of old gardens with the construction of new houses and a square, and improved sanitation.
d) Pirajá 2019, 2021 and 2023 (PI2019, PI2021 and PI2023): This community is adjacent to the Cobre reservoir, approximately 1,000m from the SB neighborhood. It also borders the São Bartolomeu city park and the Cobre reservoir. Here some daily activities are gardening, recreation, bathing, fishing, and washing animals. *Biomphalaria* *glabrata*. shedding *S. mansoni* cercariae have been found also in these waters (Zanardi et al., 2019a). In 2014, two condominiums from MCMV program were built in this neighborhood receiving old residents from SB.
e) Lagoa da Paixão 2022 (LP2022): This community borders lake Lagoa da Paixao, the source of the Cobre River. The community initially consisted of squatters in informal housing. The community expanded its traditional size in 2013 and 2015 with the opening of four condominiums from MCMV program bringing new residents from other areas of the city.
f) Bate-Facho 2023 (BF2023): This community is next to Pituaçu Park which is part of the Pituaçu River Basin and reservoir. It consists of informal housing arrayed along a stretch of the river’s immediate outflow from its dam. The city’s zoonosis control center has frequently identified snails shedding cercariae in areas associated with this river. Despite the low prevalence of schistosome infection in residents from pilot studies, the Municipal Department of Health requested us to perform a survey of the area in 2023.
