# Supplementary material for: Schistosomiasis as an urban disease: 13 years of studies in the neighborhoods of Salvador, BA, Brazil
Source: PLoS Negl Trop Dis. 2026 Oct 23;20(10):e0013418. doi: 10.1371/journal.pntd.0013418 (PMC13734582; doi:10.1371/journal.pntd.0013418)
Supplement: Supplementary file 4 [file pntd.0013418.s005.docx]

Response to critiques

PNTD-D-25-01291

Schistosomiasis as an urban disease: 13 years of studies in the neighborhoods of Salvador, Bahia, Brazil

PLOS Neglected Tropical Diseases

**Journal Requirements:**

1) CRediT author contributions. Oversight. Contributions listed in the manuscript, but perhaps not in the system. Will add contributions in on submission form.

2) Author summary added.

3) Should not be unpublished, but rather personal communication by one of the authors who discussed the issue with the workers. Is personal communication allowed?

4) The following statement was added to the Author Statements “Written informed consent was obtained from all participants or their guardians.”

5) Done

6) All maps were open source as now indicated in the figure legends. Previous inset was believed to be from the Brazilian census, but could not be verified and was substituted for one from Wikimedia Commons with attribution.

7) This is the complete functional link address: dx.doi.org/10.25833/fhx7-7127

8) The following was added as Acknowledgement: "This work was supported by the National Institutes of Health (NIH R01AI121330 to REB), the Fundação de Amparo à Pesquisa do Estado da Bahia (FAPESB/PRONEX PNX0001/2015 to MGR) and the Coordenação de Aperfeiçoamento de Pessoal de Nível Superior - Brasil (CAPES Finance Code 001 to PSM). The funders had no role in study design, data collection and analysis, decision to publish, or preparation of the manuscript. REB received partial salary funding from NIH.

Added: **Author Summary.**

Rapid urbanization and the lack of accompanying sanitation is urbanizing diseases previously considered rural. While sanitation in Salvador is generally good, *Schistosoma mansoni* infection was identified in all poor neighborhoods examined, and most evidence indicates this is a result of active transmission. Studies in urban environments pose difficulties not experienced or experienced to a lesser degree in most rural areas. Participation in research is lower, and violence is generally higher. Urban areas also offer opportunities due to the greater impact of infrastructure modifications and population density. Interventions tend to more easily affect a greater number of people. The prevalence of infection and the size of the city make urban infection an important focus for management. Brazil may preview what to expect in other rapidly urbanizing areas of the world.

Reviewer 1

1. Manuscript now with line numbers. Active link to raw data provided.
2. Study design clarified: *Methods* now describe a multi-site prospective design with cross-sectional and longitudinal components (six neighborhoods, 2011–2024), specifying which were single vs repeated surveys.
3. Added clarification that residence near surface water and the capacity to sample up to 2,000 participants per survey were determinants of site selection.
4. Flow diagram added in *Methods* to show study design and another in Results showing recruitment, inclusion/exclusion numbers, and follow-up by neighborhood and time point.
5. Sample size calculation described as based on detecting both prevalence differences and odds ratios, assuming 80% power and 5% significance level.
6. Models adjusted for demographic and socioeconomic covariates (sex, age, sanitation, travel, etc.) to account for potential social confounders.
7. Local vs imported infections differentiated: Added *Results 3.3 (Demographic patterns and migration)* with data on birthplace, travel history, and occupational exposure. *Discussion* emphasizes local transmission dominance, supported by parasite genetic data (Refs. 7, 9) and spatial association with surface water.
8. Participation bias and representativeness discussed: Neighborhoods were selected based on proximity to surface water and are not representative of the entire city. Added to *Limitations* in *Conclusions*.
9. Contextual environmental information added: New *Results 3.5 (Environmental and structural changes)* describes *Minha Casa Minha Vida (MCMV)* projects, sanitation upgrades, and paving of water-contact points; discussed in relation to declining prevalence.
10. Flow and consistency improved: *Introduction* aims rewritten to align with revised results; *Discussion* restructured for logical sequence (in order: temporal change, factors associated with infection, migration, sanitation, control implications).

Reviewer 2

11. Overall clarity improved: Strengthened narrative coherence between objectives, analyses, and implications. *Introduction* revised with explicit study aims; *Conclusions* rewritten to summarize key findings, limitations, and implications for surveillance and control.
12. Prevalence and incidence clarified: Defined “apparent incidence” in *Methods 2.6* and described its estimation in *Results 3.4*.
13. Limitations integrated: Incorporated into *Conclusions*, including site-selection bias (water-adjacent zones), partial neighborhood coverage, uneven survey intervals, and infrastructural/socioeconomic changes during data collection.
14. Public-health relevance emphasized: *Discussion* and *Conclusions* now state that, despite declining prevalence, urban schistosomiasis remains a relevant public-health concern. Quantitative comparison: “Even if 1% of Salvador’s population were infected, this would exceed the total infected population of 70% of municipalities in Bahia.” Highlighted the need for continued surveillance, targeted control, and integration of sanitation planning.
15. Comparative context expanded: *Discussion* compares observed age-sex and sanitation patterns with previous rural and peri-urban studies in Brazil and elsewhere (Refs. 19–24), clarifying differences in population mobility, occupational exposure, and challenges unique to urban control.

Reviewer 3

16. Readability and flow improved: Manuscript reorganized into a coherent structure with consistent transitions between *Introduction*, *Methods*, *Results*, and *Discussion*.
17. Figures enhanced: All figures rebuilt at high resolution with standardized legends, scales, and captions; added schematic overview (Figure 2) and flow diagrams (Figures 1 & 3).
18. Diagnostic detail expanded: *Methods 2.5* now describes Kato-Katz examination of three stool samples per participant, slide counts, and 30-day post-treatment re-examination. Distinction between reinfection and persistent infection clarified.
19. Causal language revised: All statements implying causation replaced with neutral phrasing. *Discussion* rewritten to emphasize observational interpretation.
20. Kato-Katz sensitivity addressed: The *Discussion* now acknowledges that “While three stool samples were examined per participant to improve diagnostic sensitivity, low-intensity infection may still have been missed. This limitation may have led underestimation of the prevalence and apparent incidence, particularly in later surveys when infection intensity was lower.”
