# Supplementary material for: Schistosomiasis as an urban disease: 13 years of studies in the neighborhoods of Salvador, BA, Brazil
Source: PLoS Negl Trop Dis. 2026 Oct 23;20(10):e0013418. doi: 10.1371/journal.pntd.0013418 (PMC13734582; doi:10.1371/journal.pntd.0013418)
Supplement: Supplementary file 5 [file pntd.0013418.s006.docx]

**To the Editor,**

We appreciate the opportunity to improve our manuscript. We believe, all review and editor requests and comments have been addressed in the following sections of this response. We await the next stage of the evaluation process.

The authors

**Point-by-point response to the Reviewer´s Comments and Suggestions for the second revision.**

Thank you very much for taking the time to review this manuscript. Please find detailed responses below and the corresponding revisions/corrections highlighted/in track changes in the re-submitted files.

**Reviewer #1**

**Methods, Results, Conclusions & Editorial and Data Presentation Modifications:** *See Summary and General Comments*

**Summary and General Comments**

*Many comments I made to the original manuscript in the annotated version were not addressed. I also make the authors note that the "track changes" version of the R1 manuscript basically did not have track changes. I still cannot reconcile the methods with the results: there are many mismatches in both concepts and numbers between methods, results text and results figures/tables and some results do not even have a link to the methods. As it stand, it would be very difficult to make line-by-line comments because that would involve all the manuscript. I am very sorry about this situation because I believe producing data about urban Sm infection would have been extremely useful, but the manuscript is still not usable.*

**Response:**

The authors apologize that we failed to notice the attachment with the initial critique as well as a failure to submit the file with tracked changes. We have now addressed the comments from this reviewer from the first critique in the revised version of the manuscript. Response to those initial comments have strengthened the introduction, clarified the study design and mirror comments of other reviewers about the methods that were addressed in the first revision. Most reviewers, however, found the revision coherent with some minor defects.

**Reviewer #2**

**Methods:** *Revised*

**Results**: *Revised*

**Conclusions:** *Revised*

**Editorial and Data Presentation Modifications:** *Revised*

**Summary and General Comments:** *Revised*

**Reviewer #4**

**Methods:**

*- Yes, the author DID NOT state in the methods section, if the participant CONSENTED to participate in the study, although it was mentioned in the results*

**Response**:

Thank you for mentioning the lack of participant consent information in the manuscript. Although this information was available in the Author´s Statements section, we agree with this comment. Therefore, we added the following phrase into the Abstract / Methods paragraph:

“All subjects provided written informed consent or in the case of minors, written consent was obtained from their guardians.”

We also moved the following paragraph from the Author´s statement to the Methods / Recruitment section:

“All subjects provided written informed consent or in the case of minors, written consent was obtained from their guardians. The study was approved by the Research Ethics Committee (CEP) of the Oswaldo Cruz Foundation, Bahia, the Brazilian Commission for Research Ethics (CONEP) under registry no. 42424915·9·0000·0040, the Institutional Review Boards of University Hospitals of Cleveland UHCMC IRB number: 01-14-32, and Tulane University administrative review 2019-1799.”

**Results**:

*The figures (except for Figure 6, which looks blurred when zoomed)*

**Response:**

All figures were saved again in high resolution, 300-600 dpi. Special attention was given to Fig 6. Each image that makes up this panel was recreated with a larger font and saved in the same way.

**Conclusions:** *Revised*

**Editorial and Data Presentation Modifications:**

*- In the methods section, the authors DID NOT indicate the time period in the day the stool samples were collected as there is a recommended optimal time for stool sampling as it influences egg detection.*

*ABSTRACT*

*1. Consented should be added to the participants*

*2. Line 41 (now 47) 'is' to be replaced by 'in'*

*3. line 45 -49 (now 44-46) too lengthy, consider breaking it into two sentences*

*4. Conclusion not too clear*

**Response:**

*-* The authors appreciate the reviewer’s comment regarding the timing of sample collection. However, it is important to clarify that the recommendation for an 'optimal time' (typically between 10:00 AM and 2:00 PM) is standard for the detection of *Schistosoma haematobium* eggs in urine due to circadian excretion patterns. For *Schistosoma mansoni*, which is the species prevalent in Salvador, Brazil, there is no established evidence in the literature suggesting that the time of day for stool collection significantly influences egg detection sensitivity. Instead, sensitivity for *S. mansoni* is primarily affected by the number of samples collected over different days and the number of slides prepared per sample (e.g., Kato-Katz method). In our study, participants were provided with containers and instructed to collect the first bowel movement of the day, following standard epidemiological protocols for field surveys in Brazil.

1. This comment has already been addressed above, with the inclusion of consent statements in the Abstract and Methods/Recruitment sections.

2. Line 41 (now 47) ‘is’ was replaced by ‘in’

*3.* Lines 45-49 (now 44-46), the referred sentence was broken into two sentences as recommended: “Overall, the study provides one of the first individual-level longitudinal assessments of schistosomiasis persistence in an urban Brazilian setting, combining repeated parasitological surveys with demographic and environmental data across multiple neighborhoods. These findings highlighting the need for integrated control strategies that address urban sanitation and surveillance.*”*

4. To take care of this comment, the Abstract / Interpretation section was revised to:

“Overall, the study provides one of the first individual-level longitudinal assessments of schistosomiasis persistence in an urban Brazilian setting, combining repeated parasitological surveys with demographic and environmental data across multiple neighborhoods. These findings indicate that schistosomiasis transmission in Salvador is largely local rather than imported. The decline in prevalence coincides with observed community-level improvements in sanitation and reduced contact with surface water. Recently published parasite genetic analysis supports localized transmission patterns, highlighting the need for integrated control strategies that address urban sanitation and surveillance. If prevalence rates were applied across the population of Salvador, this city could be considered one of the state’s most important endemic areas.”

*INTRODUCTION*

*Line 96 (now 99) comma (,) after Salvador*

*Line 118 (now 121) full stop (.) after confidence*

*Line 167 (now 177) soil transmitted helminth replaced with soil-transmitted helminths*

**Response:**

All minor revisions have been made.

*RESULTS*

*Line 202 (now 212), add 's' to male*

*Line 205-206 (now 215-216) add 'of the participants'*

*Line 210 (now 220) mention some of the similar characteristics*

*Line 244 (now 254) add "with" after 100%*

*Line 293 (now 303) add full stop (.) after treatment*

*Line 336-337 (now line 346-348) "in part the areas’ very vitality" This sentence is not clear*

*Line 348-350 the sentence not too clear, re-word it*

**Response:**

All minor revisions have been made.

Line 336-337 (now line 343-345) was rephrased to:

“We have studied schistosomiasis using similar approaches in both rural and urban areas. One of the main challenges for controlling schistosomiasis in urban areas lies in part with urban dynamics. Urban populations are more internally mobile and have more intense commercial activity.”

Line (348-350) (now 357-360) was reviewed to:

“Given urban density, any control measure will affect a much larger number of many more individuals. Also, since the area is more compact, structural changes to entire neighborhoods can be contemplated. Hopefully, not all changes involve cementing over rivers and streams, but emphasize preventing feces going in rather than restricting access to them.”

**Summary and General Comments:**

*This manuscript presents a long-term, longitudinal investigation of the transmission of schistosomiasis in urban areas in Salvador, Brazil. It was based on parasitological surveys conducted over 13 years involving multiple urban neighborhoods. The study addressed an important and underexplored issue: the persistence of the transmission of schistosomiasis in urban settings in spite of improvements in sanitation coverage. Overall, the manuscript is methodologically sound, scientifically robust, and highly relevant to public health and*

*management of neglected tropical diseases in increasingly urbanizing environments. The integration of spatial and environmental data, individual-level follow-up and longitudinal design are important strengths. With minor revisions as outlined, the manuscript is suitable for publication*

**Response:**

The authors are sincerely grateful to the reviewer for the thorough and supportive assessment of our work.
