# Supplementary material for: Schistosomiasis as an urban disease: 13 years of studies in the neighborhoods of Salvador, BA, Brazil
Source: PLoS Negl Trop Dis. 2026 Oct 23;20(10):e0013418. doi: 10.1371/journal.pntd.0013418 (PMC13734582; doi:10.1371/journal.pntd.0013418)
Supplement: Supplementary file 6 [file pntd.0013418.s007.docx]

Authors’ Responses

Reviewer #1

There is a mismatch between methods and results and consequently conclusions. Please see the "general comments" for a point by point detail of the issues

Line 48-50: improvement in sanitation and contact with water are mentioned in the “interpretation” section of the abstract but there is no mention in the methods of such data being collected

**This refers to a comparison published in 2015. The methods used in that study are now included in the methods section Lines 169-174.**

Line 106: No formal comparison of urban vs rural

**Done. Publications for rural studies reference on Lines 359-375**

Write clearly how household members were chosen.

**To line 160 we added “For baseline and follow-up studies” to “…all households… and ” Data from the interviews for all household members were directly entered into Android-based devices (Android version 9·1, Samsung Galaxy Tab A 8·0) using a REDCap tool (version 13·1·26-2024) ^22^”**

Sample size calculation was 72 but 100 targeted in pilots and >6000 collected during the study. Explain

**The pilot study was a sample, thus a sample size was calculated, and this was per neighborhood. It is routine to collect above this number to account for unexpected losses (failure to provide both demographic information and fecal sample). The pilot was to identify the population to be studied where all willing subjects were included and not a sample. As described, all households and all household members were examined.**

It is unclear if the same individuals were re-examined.

**The reviewer has correctly observed that both the same individuals and some newly enrolled individuals were examined in subsequent years.**

Line 260-263 No data about occupation or water contact in text or raw data file

**Raw data file has been modified to include economic class and number of surface water sites contacted.**

Lin 269. Migrants not defined

**Definition of migrants now provided in methods lines 207-208.**

Reviewer #4

No comments

Reviewer #5

Line 122. Why were 72 individuals examined?

**A minimum of 72 was calculated to give sufficient power for the sample which was the pilot. It is common practice to add 20-30% above the minimum to cover potential losses, incomplete data etc. To clarify line 119 was amended to: “A sample of at least 72 individuals was calculated to identify an infection prevalence of 5% with 95% confidence.”**

Line 174. Quantity of stool examined should be stated.

**Done. The quantity of stool examined (38.5 mg) has been included on line 179.**
