# Supplementary material for: Schistosomiasis as an urban disease: 13 years of studies in the neighborhoods of Salvador, BA, Brazil
Source: PLoS Negl Trop Dis. 2026 Oct 23;20(10):e0013418. doi: 10.1371/journal.pntd.0013418 (PMC13734582; doi:10.1371/journal.pntd.0013418)
Supplement: Supplementary file 8 [file pntd.0013418.s009.docx]

Response to Reviewers

Reviewer #1	None of previous observations addressed

We find ourselves again having to apologize for the oversight. Only some of the comments were addressed. We have in fact found this reviewer’s comments quite helpful. We will respond to the original critique from reviewer #1 first followed by responses to the second review.

**Editorial and Data Presentation (from 1st review)**

Reviewer #1 (from 1^st^ review)

Line 37 Kato-Katz now cited in the abstract

Line 38 Revised

Author Summary: Should be a lay version of the abstract. As indicated in the Author Guidelines, the Author Summary should be distinct from the abstract. In fact “lay” is never mentioned. The instructions ask authors to “highlight how the work fits in a broader context of life science knowledge and why these findings are important to an audience that includes both scientists and non-scientists. “

Line 73-74 “but” removed.

Line 75 rephrased

Line 77 Find a better reference. Done

Line 91 Reference was substituted

Line 93: Write out *Schistosoma haematobium*	Done

Line 90-91 Revise as recommended	Done

Line 138-139 Amended to : The observed usage of these waters by the population for work, fishing and recreation with was an additional criterion.

Line 221 (now line 231) “Some” was replaced with “Any” as in Table 1.

Line 233-234 Done

Line 245: The reviewer’s suggestion is now addressed in the discussion section rather than in the results. Line 346-348 revised manuscript

Line 259 We understand the suggestion, but “sons” was used since only sons worked in the plots and all were older than 18.

Line 265-266 and 285. For lines 265-266 we don’t have a number, but lower than the year before. For line 285 (Line 290 revised manuscript-Prevalence decreased substantially), exact numbers are given in the text and table after this statement.

Line 283-284. Need to calculate % reinfection vs new infections

Lines 290-295 revised manuscript The incidence of infection and % reinfection are cited in this parafraph.

Line 287-288. We have changed the wording of round to “study period” or “treatment period”. We cannot agree that treatment had no effect, although we emphasize that it may not be the only factor long-term, however, socio-economic changes from one year to the next are unlikely to explain a steep decline in prevalence where the interval between study periods was 1 year. We think it was clearly a factor in São Bartolomeu where there had been an intentional improvement in housing, and 5 years had passed between interventions. Both treatments and changes in economic conditions may have played a role.

The buffer areas were designed to include a total population of 2000-2500. % interviewed and % with stool exams (ll 223-227). These results also available in Table 3.

Discussion: Zoonotic transmission

Trapping rodents in the city for leptospirosis studies identified primarily Norway rats that can be infected but are not permissive. Very few *Rattus rattus* trapped were trapped and no *Nectomys squamipes* both of which are permissive hosts. Lines 397-399 (revised manuscript).

Line 312-315: this sentence is unclear, seems repetitious. The second sentence was eliminated.

Line 317: ref 31 is about Ethiopia, not Brazil.

Reference was deleted.

Line 338-340. The phrase “…through local transmission” was removed.

Line 350. Please provide the basics of this concept.

We feel that only a few additional lines would not clarify the concepts significantly more for most readers and more lines would become unwieldly.

Line 357-358 References to studies in rural areas had been included

These references were inclded [28,37,38].

Line 376-378 Please clarify.

The sentence was amended.

Line 392-397: Limitations moved to discussion

**Summary and General Comments (1^st^ review)**

Reviewer #1

Major issues

Line 48-50 No mention of changes in sanitation.

The changes in sanitation refers mainly to results in São Bartolomeu where urban renewal took place and an evaluation made before and after. We also showed advance in economic status. This was published [reference 27] and results referred to in the text [Lines 234-237]. There is sanitation data presented in tables where adequate sanitation went from 67.8 to 95%. In other areas we see only minimal change, but baseline coverage is reportedly high and the interval between survey periods was short. The statement in the methods that number of surface water sites contacted was included in the epidemiologic survey.

Line 106: the point iv not covered.

Removed.

How were households and participants selected?

Within the buffer zone all households were invited to participate, and all household members were invited to enroll. After the buffer zone was drawn, there was no further selection. This is emphasized in the revised manuscript. (Lines 165-174)

Why 6000 needed when sample size was 72.

The sample size calculation was for the pilot studies. This was used to select neighborhoods. The study itself was of the neighborhood and neighborhood dynamics. The impact of treating most infections would not be observed treating 72 individuals.

Are the same individuals examined on follow-up surveys?

Yes, and those not enrolled in the previous survey were allowed to enroll in subsequent surveys. This is obviated in the revised manuscript Lines 131-132.

Line 260-263 Water contact not mentioned

Occupations and water contact are now included in the raw data files. These are as the respondents describe their work.

Line 269: “Migrants” was defined on line 207 (213-214 revised manuscript)

Reviewer #5

Lines 120-123. Why more than 72 enrolled

A sample size calculation indicated that 72 individuals would give sufficient power for the pilots whose function was to identify a neighborhood for study. We elected to examine 10% over this number thus aimed to collect 100 samples. The description of the pilots has been amended for clarity in the revised manuscript.

Line 174 Kato-Katz stool mass added (Line 185 Revised manuscript 41.7 mg)

---------------------------------------------------------------------------------------------

**Methods**

Reviewer #8 Statistics: age should be displayed as median with interquartile range

We thank the reviewer for this insightful comment. We understand that, in isolation, certain variables would be more accurately represented by the median and interquartile range (IQR). However, we chose to present all variables in [Table X / Figure X] using the mean and standard deviation (SD) for the sake of methodological consistency and visual standardization. Since most variables in our study follow a normal distribution and are best represented by the mean, mixing different descriptive statistics within the same display would complicate the presentation and reduce the clarity of the illustration. We believe that maintaining a homogeneous format simplifies the overall data interpretation for the reader without compromising scientific rigor.

**Results**

Reviewer #8: Figure 2 legend is awkward.

Data was moved to supplementary table.

**Editorial and Data Presentation (from 2nd review)**

Reviewer #1. Apologies again as above.

Reviewer #8

Comment 1	Line 227 Report median age and interquartile range

Response as above

Comment 2	Figure 2 table moved to supplemental data

Comment 3	Critique noted and some changes performed.

**Specific comments**

Line 93: Write out *S. haematobium*.

Done

Line 37 Delete “Some”

Done

Line 62-64 What opportunities?

The phrase “…for effective intervention” added. There were 2 phrases immediately following that indicated what were the opportunities. These were further detailed in the discussion (LL. 377-383).

Line 66-67. The sentence was rephrased.

Line 90 The sentence was rephrased

**Summary and General Comments 2nd review)**

Reviewer #1	As noted above. We did indeed overlook the reviewer’s comments and apologize.

Reviewer #7

Lines 135-136 We now provide a clarification statement on which data were previously published.

Line 100-102 The 3 single, cross-sectional studies are now mentioned here.

Line 117. The timing of studies is in the table now in Supplemental data. The table was previously in Figure 2.

Line 118. We now specify that they were shedding *S. mansoni* cercariae and *S. mansoni* infections were recorded.

Line 120 The Kato-Katz is now specified (Line 125 revised manuscript)

Line 122 The basis of the sample size for the pilots is now included in lines 121-124.

Line 140 See Lines 135-136 above.

Figure 3. Also make clear that these are single surveys at different times.

These surveys are presented in separate boxes with the dates of study associated with their labels.

Reviewer #8. Revise language to be precise, specific and suitable for a scientific audience.

There were some places where the language could and should have been more precise. e.g. The use of “some” when the following statement was an exact number, “stool examination” when Kato-Katz was the precise examination. These and other areas have been addressed. The language at the point of saying what the study means as we see the results vs what the data say does seem appropriate to us. Plain language is appropriate as long as it is accurate.
