# Supplementary material for: Schistosomiasis as an urban disease: 13 years of studies in the neighborhoods of Salvador, BA, Brazil
Source: PLoS Negl Trop Dis. 2026 Oct 23;20(10):e0013418. doi: 10.1371/journal.pntd.0013418 (PMC13734582; doi:10.1371/journal.pntd.0013418)
Supplement: Supplementary file 9 [file pntd.0013418.s010.docx]

Response to Reviewers

Dear Editors,

The attachment from Reviewer #1 is from an earlier version to which I have responded. Reviewer #7 place edits and comments on the manuscript v.9. We have accepted most of these edits and responded to the comments on the Revised Article with Changes Highlighted.

The Authors
