# Supplementary material for: Schistosomiasis as an urban disease: 13 years of studies in the neighborhoods of Salvador, BA, Brazil
Source: PLoS Negl Trop Dis. 2026 Oct 23;20(10):e0013418. doi: 10.1371/journal.pntd.0013418 (PMC13734582; doi:10.1371/journal.pntd.0013418)
Supplement: Peer review history

# Peer review: Decision Letter 0

**DOI:** 10.1371/journal.pntd.0013418.r001

**Authors:** jong-Yil Chai, Feng Xue

28 Aug 2025

PNTD-D-25-01291

Schistosomiasis as an urban disease: 13 years of studies in the neighborhoods of Salvador, Bahia, Brazil

PLOS Neglected Tropical Diseases

Dear Dr. Blanton,

Thank you for submitting your manuscript to PLOS Neglected Tropical Diseases. After careful consideration, we feel that it has merit but does not fully meet PLOS Neglected Tropical Diseases's publication criteria as it currently stands. Therefore, we invite you to submit a revised version of the manuscript that addresses the points raised during the review process.

Please submit your revised manuscript within 60 days Oct 27 2025 11:59PM. If you will need more time than this to complete your revisions, please reply to this message or contact the journal office at plosntds@plos.org. When you're ready to submit your revision, log on to https://www.editorialmanager.com/pntd/ and select the 'Submissions Needing Revision' folder to locate your manuscript file.

Please include the following items when submitting your revised manuscript:

* A rebuttal letter that responds to each point raised by the editor and reviewer(s). You should upload this letter as a separate file labeled 'Response to Reviewers'. This file does not need to include responses to any formatting updates and technical items listed in the 'Journal Requirements' section below.

* A marked-up copy of your manuscript that highlights changes made to the original version. You should upload this as a separate file labeled 'Revised Manuscript with Track Changes'.

* An unmarked version of your revised paper without tracked changes. You should upload this as a separate file labeled 'Manuscript'.

If you would like to make changes to your financial disclosure, competing interests statement, or data availability statement, please make these updates within the submission form at the time of resubmission. Guidelines for resubmitting your figure files are available below the reviewer comments at the end of this letter.

We look forward to receiving your revised manuscript.

Kind regards,

Feng Xue, Ph.D.

Guest Editor

PLOS Neglected Tropical Diseases

Jong-Yil Chai

Section Editor

PLOS Neglected Tropical Diseases

Shaden Kamhawi

co-Editor-in-Chief

PLOS Neglected Tropical Diseases

orcid.org/0000-0003-4304-636XX

Paul Brindley

co-Editor-in-Chief

PLOS Neglected Tropical Diseases

orcid.org/0000-0003-1765-0002

**Journal Requirements:**

1) Please ensure that the CRediT author contributions listed for every co-author are completed accurately and in full.

At this stage, the following Authors/Authors require contributions: Vanessa T. Moretto, and Ronald Edward Blanton. Please ensure that the full contributions of each author are acknowledged in the "Add/Edit/Remove Authors" section of our submission form.

The list of CRediT author contributions may be found here: https://journals.plos.org/plosntds/s/authorship#loc-author-contributions

2) Please provide an Author Summary. This should appear in your manuscript between the Abstract (if applicable) and the Introduction, and should be 150-200 words long. The aim should be to make your findings accessible to a wide audience that includes both scientists and non-scientists. Sample summaries can be found on our website under Submission Guidelines:

https://journals.plos.org/plosntds/s/submission-guidelines#loc-parts-of-a-submission

3) We noticed that you used the phrase 'unpublished ' in the manuscript. We do not allow these references, as the PLOS data access policy requires that all data be either published with the manuscript or made available in a publicly accessible database. Please amend the supplementary material to include the referenced data or remove the references.

4) Thank you for including an Ethics Statement for your study. Please include:

i) A statement that formal consent was obtained (must state whether verbal/written) OR the reason consent was not obtained (e.g. anonymity). NOTE: If child participants, the statement must declare that formal consent was obtained from the parent/guardian.].

5) Tables should not be uploaded as individual files. Please remove these files and include the Tables in your manuscript file as editable, cell-based objects. For more information about how to format tables, see our guidelines:

https://journals.plos.org/plosntds/s/tables

6) Some material included in your submission may be copyrighted. According to PLOSu2019s copyright policy, authors who use figures or other material (e.g., graphics, clipart, maps) from another author or copyright holder must demonstrate or obtain permission to publish this material under the Creative Commons Attribution 4.0 International (CC BY 4.0) License used by PLOS journals. Please closely review the details of PLOSu2019s copyright requirements here: PLOS Licenses and Copyright. If you need to request permissions from a copyright holder, you may use PLOS's Copyright Content Permission form.

Please respond directly to this email and provide any known details concerning your material's license terms and permissions required for reuse, even if you have not yet obtained copyright permissions or are unsure of your material's copyright compatibility. Once you have responded and addressed all other outstanding technical requirements, you may resubmit your manuscript within Editorial Manager.

Potential Copyright Issues:

i) Figure 1. Please (a) provide a direct link to the base layer of the map (i.e., the country or region border shape) and ensure this is also included in the figure legend; and (b) provide a link to the terms of use / license information for the base layer image or shapefile. We cannot publish proprietary or copyrighted maps (e.g. Google Maps, Mapquest) and the terms of use for your map base layer must be compatible with our CC BY 4.0 license.

Note: if you created the map in a software program like R or ArcGIS, please locate and indicate the source of the basemap shapefile onto which data has been plotted.

If your map was obtained from a copyrighted source please amend the figure so that the base map used is from an openly available source. Alternatively, please provide explicit written permission from the copyright holder granting you the right to publish the material under our CC BY 4.0 license.

If you are unsure whether you can use a map or not, please do reach out and we will be able to help you. The following websites are good examples of where you can source open access or public domain maps:

* U.S. Geological Survey (USGS) - All maps are in the public domain. (http://www.usgs.gov)

* PlaniGlobe - All maps are published under a Creative Commons license so please cite u201cPlaniGlobe, http://www.planiglobe.com, CC BY 2.0u201d in the image credit after the caption. (http://www.planiglobe.com/?lang=enl)

* Natural Earth - All maps are public domain. (http://www.naturalearthdata.com/about/terms-of-use/).

ii) Figure 4: Thank you for stating "Source: Open Street Map (https://www.openstreetmap.org/). Please note, the maps sourced form OpenStreet maps, need proper attribution in the figure legend, please see https://osmfoundation.org/wiki/Licence/Attribution_Guidelines

7) Thank you for indicating that "De-identified individual participant data collected during the study is freely available through the Digital Object Identifier (DOI 10.25833/1acj-zj58)." Please provide a direct link to access the dataset.

8) Please amend your detailed Financial Disclosure statement. This is published with the article. It must therefore be completed in full sentences and contain the exact wording you wish to be published.

1) State the initials, alongside each funding source, of each author to receive each grant. For example: "This work was supported by the National Institutes of Health (####### to AM; ###### to CJ) and the National Science Foundation (###### to AM)."

2) State what role the funders took in the study. If the funders had no role in your study, please state: "The funders had no role in study design, data collection and analysis, decision to publish, or preparation of the manuscript."

3) If any authors received a salary from any of your funders, please state which authors and which funders.

Note: If the reviewer comments include a recommendation to cite specific previously published works, please review and evaluate these publications to determine whether they are relevant and should be cited. There is no requirement to cite these works unless the editor has indicated otherwise.

**Comments to the Authors:**

**Please note that one review is uploaded as an attachment.**

**Reviewers' Comments:**

Reviewer's Responses to Questions

**Key Review Criteria Required for Acceptance?**

As you describe the new analyses required for acceptance, please consider the following:

**Methods**

-Are the objectives of the study clearly articulated with a clear testable hypothesis stated?

-Is the study design appropriate to address the stated objectives?

-Is the population clearly described and appropriate for the hypothesis being tested?

-Is the sample size sufficient to ensure adequate power to address the hypothesis being tested?

-Were correct statistical analysis used to support conclusions?

-Are there concerns about ethical or regulatory requirements being met?

Reviewer #1: Since there were no row numbers, it is impossible to provide a line-by-line reference to the comments, so please refer to the annotated text for precise issues to be addressed. In general, the methods are very unclear. The reader gets an idea of what was done by reading methods, results and discussion, but instead the structure of the study should be clearly presented (even with the aid of a flow diagram or something like that) straight from the methods. Then the results and the discussion should follow the same structure for clarity. the raw data are unaccassible: the DOI provided is not opening (at least, I could not find it by copy-pasting in the browser) so I could not check the original questionnaire. It seems that some important questions (e.g. occupation and exposure to water) were not asked, or maybe asked and not analyzed. It is very unclear how the sample size was worked out, how sampling was done at T0 and at following time points.

Reviewer #2: see the comments, please

Reviewer #3: If possible, please provide more details on the association between the sample size and the choice of statistical tests.

**********

**Results**

-Does the analysis presented match the analysis plan?

-Are the results clearly and completely presented?

-Are the figures (Tables, Images) of sufficient quality for clarity?

Reviewer #1: Since methods were not very clear, also results did not read very clearly (see annotated manuscript). Some results are not mentioned in the methods and not thoroughly presented in tables. Figures have very low quality even when accessing them from the link provided, so it was impossible for me to revise them.

Reviewer #2: adequate

Reviewer #3: Results are clearly presented

**********

**Conclusions**

-Are the conclusions supported by the data presented?

-Are the limitations of analysis clearly described?

-Do the authors discuss how these data can be helpful to advance our understanding of the topic under study?

-Is public health relevance addressed?

Reviewer #1: In the discussion is diffucult to discern what is discussed from the results of THIS paper and what from previous/other studies. Some paragraphs are very unclear and require reqìvision (see always annotated manuscript). There in no real "conclusions" while the study limitations should be integrated in the text and commented, rather than provided as a final list similar to a bullet point list.

Reviewer #2: Are the conclusions supported by the data presented? Partially

-Are the limitations of analysis clearly described? Partially

-Do the authors discuss how these data can be helpful to advance our understanding of the topic under study? Partially

-Is public health relevance addressed? Yes

Reviewer #3: Discussion:

The discussion should explore more deeply the comparative perspective: how do these results align with or differ from findings in other endemic regions?

Some sections read more like a repetition of the results; the authors should be encouraged to focus more on interpretation.

For example: The sentences below could be improved by integrating them with findings from other studies, rather than simply restating the results.

Salvador through local transmission. In neighborhoods such as São Bartolomeu in 2011, where only 67·8% of households had inadequate sewage disposal, the prevalence of S. mansoni was significantly higher;

By contrast, in this study, across all neighborhoods, urban schistosomiasis, was most prevalent in the age group 21–30 years, with certain neighborhoods such as São Bartolomeu in 2011 peaking in an even older age groups (31–40 years);

Infection prevalence was related to the proximity of homes near surface water bodies, except in the case of São Bartolomeu in 2011. We believe that transmission there was sufficiently high at this time that no correlation could be observed at the narrow geographic scale studied. The relationship of distance to a water body and risk may be dependent on degree of sanitation as well as scale since sanitation was also poorest in São Bartolomeu in 2011.

… and others.

Conclusions:

My suggestion is that the conclusion could be more concise and directly aligned with the research objectives, with stronger emphasis on the practical impact of the study (e.g., implications for One Health, zoonoses, or surveillance).

**********

**Editorial and Data Presentation Modifications?**

Use this section for editorial suggestions as well as relatively minor modifications of existing data that would enhance clarity. If the only modifications needed are minor and/or editorial, you may wish to recommend “Minor Revision” or “Accept”.

Reviewer #1: The text overall needs substantial revision for clarity. many statements of the introduction and discussion are missing references

Reviewer #2: need modifications

Reviewer #3: (No Response)

**********

**Summary and General Comments**

Use this section to provide overall comments, discuss strengths/weaknesses of the study, novelty, significance, general execution and scholarship. You may also include additional comments for the author, including concerns about dual publication, research ethics, or publication ethics. If requesting major revision, please articulate the new experiments that are needed.

Reviewer #1: The authors present an interesting topic and aspect of schistosomiasis. However, the confusing presentation does not allow a thorough appraisal of the manuscript and its content. Please reefre to the annotated manuscript for point-by-point issues that I suggest to address.

Reviewer #2: The study addresses an important topic: the persistence and transmission of a traditionally rural parasite within rapidly urbanising settings. The integration of demography, sanitation indicators, and geospatial proximity to water is a key strength, and the longitudinal element across neighbourhoods adds valuable insight.

However, several important methodological and interpretive issues require clarification and strengthening.

General remarks

1) Clarity and consistency of study design

- The manuscript oscillates between cross-sectional, longitudinal, and repeated-treatment frameworks. Some sites were surveyed once, others multiple times, with follow-ups 1–5 years apart. A single, clear design description is essential (e.g., “multi-site prospective with repeated cross-sectional surveys in six neighbourhoods, with treatment cycles following positive diagnoses”). The current text blurs between study types, and this confuses causal interpretation.

- The abstract uses causal language about decreases in prevalence following interventions. Given the observational, quasi-longitudinal design, temper causal claims or reframe them as associations observed in the context of programmatic interventions.

2) Incidence vs prevalence and interpretation of temporal trends

- The abstract states that incidence decreased to <0.5%/year following a second round of treatment. How is incidence estimated in this study design (especially with intermittent follow-up up to 5 years and multiple surveys per site)? Provide a clear definition, calculation method (person-time at risk, number of new infections per person-year, etc.), and assumptions. If incidence is not directly estimable, this should be acknowledged and reframed as a decline in observed new infections or apparent risk after interventions.

- When reporting declines, present time-specific prevalence and incidence estimates with confidence intervals and the number at risk for each interval. If possible, include a figure showing prevalence over time by site.

3) Representativeness and generalizability

- The sampling was a convenience sample of 100 residents per neighbourhood initially, focusing on areas near water bodies with heavy faecal contamination. The six neighbourhoods with follow-up are not necessarily representative of Salvador as a whole. The authors should explicitly acknowledge potential selection bias and limit overgeneralisation to the city or state endemic areas. Consider including a supplementary section on limitations of external validity.

4) Attrition, follow-up, and potential bias

- Of 13,040 residents in the buffer areas, 10,383 were contacted and consented, with 6,863 participating. Please quantify and discuss nonresponse bias, differential loss to follow-up, and how these might affect prevalence and incidence estimates over time.

- Provide a flow diagram of recruitment, participation, follow-up, and treatment, including reasons for non-participation and loss to follow-up by neighbourhood and time point.

5) Diagnostic methods and disease status classification

- While Kato-Katz with three stool samples improves sensitivity, limitations remain, especially for low-intensity infections. The authors should discuss diagnostic sensitivity/specificity, potential misclassification at baseline and follow-ups, and how this might influence observed declines.

- Define “egg-negativity” criteria precisely (e.g., three consecutive negative stool samples across the same individual, or other confirmatory tests). Clarify how re-infection vs. persistent infection was distinguished, particularly with long intervals between assessments.

6) Causality and contributing factors

- The manuscript references “structural changes in the communities that improved sanitation and prevented contact with surface waters” as drivers of improvement. Provide concrete evidence for these changes (e.g., data on latrine coverage, water supply improvements, snail habitat modification, street/policy changes) or temper this claim accordingly. If such data are available, present them; if not, frame as a plausible contributor rather than a proven cause.

- Environmental and social confounders (e.g., economic shifts, migration patterns, parallel public health campaigns) should be discussed. Consider adjusting analyses for these factors or performing sensitivity analyses if data exist.

7) Genetic data and interpretation

- The abstract mentions population genetic studies of the parasite supporting local transmission, but the Methods/Results presented do not include any genetic analyses. If genetic data are part of the manuscript, they should be described and integrated with explicit methods and results. If not, either remove the genetic claim or provide a clear plan for integrating genetic data in the final manuscript. Inconsistency between the abstract and body text weakens credibility.

8) Language, clarity, and consistency

- Minor but important: ensure consistency in species nomenclature (Schistosoma mansoni),

- Some sections would benefit from tighter editing to improve readability and logical flow, especially the transition from broad urbanisation context to the study-specific aims.

Specific questions that need to be addressed

- How is incidence defined and calculated in this framework? Provide the formula and assumptions.

- What is the exact follow-up scheme for each neighbourhood, including numbers at risk at each time point?

- How do you distinguish re-infection from persistent infection after treatment, given follow-up intervals up to 5 years?

- What concrete sanitation and water-contact changes occurred in the study communities, and how were these measured?

- Do you have genetic data to support local transmission? If yes, provide methods and results; if not, remove the genetic language from the abstract.

- Please provide a complete Results section (mend the truncated sentence and supply all numbers, CIs, and p-values).

Recommendation

- Major Revision. The study tackles an important topic with rich, longitudinal, geospatial data. However, to meet the standards of rigorous epidemiology and to support the conclusions drawn, the manuscript requires:

- A clearly defined study design and consistent terminology.

- Explicit handling of incidence estimation and temporal trends.

- Transparent discussion of biases, loss to follow-up, and generalizability.

- Complete results with complete tables/figures and properly qualified claims.

- Alignment of abstract and main text (and resolution of any genetic data claims that are not presented in the body).

Reviewer #3: The abstract is informative, but it could be clearer in highlighting the novelty of the study, please verify.

Please revise the sentence referring to Brazilian urbanization. My suggestion is to make a change, because the term urbanized does not necessarily reflect the reality experienced by most people living in urban regions. While urbanized often refers to areas with high population density and significant infrastructure, the lived experience of urban areas in Brazil can vary widely. Unfortunately, Brazil faces serious infrastructure problems, even within urban areas. My suggestion is: Today, in Brazil, a higher proportion of people live in cities compared to the United States, with over 88% of the Brazilian population residing in urban areas.

**********

PLOS authors have the option to publish the peer review history of their article (what does this mean?). If published, this will include your full peer review and any attached files.

If you choose “no”, your identity will remain anonymous but your review may still be made public.

**Do you want your identity to be public for this peer review?**  For information about this choice, including consent withdrawal, please see our Privacy Policy.

Reviewer #1: No

Reviewer #2: **Yes:**  Ahmed Hassan Fahal

Reviewer #3: **Yes:**  Eduardo José Lopes-Torres

[NOTE: If reviewer comments were submitted as an attachment file, they will be attached to this email and accessible via the submission site. Please log into your account, locate the manuscript record, and check for the action link "View Attachments". If this link does not appear, there are no attachment files.]

**Figure resubmission:**

While revising your submission, we strongly recommend that you use PLOS’s NAAS tool (https://ngplosjournals.pagemajik.ai/artanalysis) to test your figure files. NAAS can convert your figure files to the TIFF file type and meet basic requirements (such as print size, resolution), or provide you with a report on issues that do not meet our requirements and that NAAS cannot fix.  After uploading your figures to PLOS’s NAAS tool - https://ngplosjournals.pagemajik.ai/artanalysis, NAAS will process the files provided and display the results in the "Uploaded Files" section of the page as the processing is complete. If the uploaded figures meet our requirements (or NAAS is able to fix the files to meet our requirements), the figure will be marked as "fixed" above. If NAAS is unable to fix the files, a red "failed" label will appear above. When NAAS has confirmed that the figure files meet our requirements, please download the file via the download option, and include these NAAS processed figure files when submitting your revised manuscript.

**Reproducibility:**

To enhance the reproducibility of your results, we recommend that authors of applicable studies deposit laboratory protocols in protocols.io, where a protocol can be assigned its own identifier (DOI) such that it can be cited independently in the future. Additionally, PLOS ONE offers an option to publish peer-reviewed clinical study protocols. Read more information on sharing protocols at https://plos.org/protocols?utm_medium=editorial-email&utm_source=authorletters&utm_campaign=protocols

Attachment Submitted filename: PNTD-D-25-01291.pdf

# Author comment: Author response to Decision Letter 1

**DOI:** 10.1371/journal.pntd.0013418.r002

27 Nov 2025

Attachment Submitted filename: Response_to_critiques_FC_v2.docx

# Peer review: Decision Letter 1

**DOI:** 10.1371/journal.pntd.0013418.r003

**Authors:** jong-Yil Chai, Feng Xue

11 Jan 2026

PNTD-D-25-01291R1

Schistosomiasis as an urban disease: 13 years of studies in the neighborhoods of Salvador, Bahia, Brazil

PLOS Neglected Tropical Diseases

Dear Dr. Blanton,

Thank you for submitting your manuscript to PLOS Neglected Tropical Diseases. After careful consideration, we feel that it has merit but does not fully meet PLOS Neglected Tropical Diseases's publication criteria as it currently stands. Therefore, we invite you to submit a revised version of the manuscript that addresses the points raised during the review process.

Please submit your revised manuscript within by Feb 10 2026 11:59PM. If you will need more time than this to complete your revisions, please reply to this message or contact the journal office at plosntds@plos.org. When you're ready to submit your revision, log on to https://www.editorialmanager.com/pntd/ and select the 'Submissions Needing Revision' folder to locate your manuscript file.

Please include the following items when submitting your revised manuscript:

* A letter that responds to each point raised by the editor and reviewer(s). You should upload this letter as a separate file labeled 'Response to Reviewers'. This file does not need to include responses to any formatting updates and technical items listed in the 'Journal Requirements' section below.

* A marked-up copy of your manuscript that highlights changes made to the original version. You should upload this as a separate file labeled 'Revised Manuscript with Track Changes'.

* An unmarked version of your revised paper without tracked changes. You should upload this as a separate file labeled 'Manuscript'.

If you would like to make changes to your financial disclosure, competing interests statement, or data availability statement, please make these updates within the submission form at the time of resubmission. Guidelines for resubmitting your figure files are available below the reviewer comments at the end of this letter.

We look forward to receiving your revised manuscript.

Kind regards,

Feng Xue, Ph.D.

Guest Editor

PLOS Neglected Tropical Diseases

Jong-Yil Chai

Section Editor

PLOS Neglected Tropical Diseases

Shaden Kamhawi

co-Editor-in-Chief

PLOS Neglected Tropical Diseases

orcid.org/0000-0003-4304-636XX

Paul Brindley

co-Editor-in-Chief

PLOS Neglected Tropical Diseases

orcid.org/0000-0003-1765-0002

**Journal Requirements:**

If the reviewer comments include a recommendation to cite specific previously published works, please review and evaluate these publications to determine whether they are relevant and should be cited. There is no requirement to cite these works unless the editor has indicated otherwise.

1) Please amend your detailed Financial Disclosure statement. This is published with the article. It must therefore be completed in full sentences and contain the exact wording you wish to be published.

1) State the initials, alongside each funding source, of each author to receive each grant. For example: "This work was supported by the National Institutes of Health (####### to AM; ###### to CJ) and the National Science Foundation (###### to AM)."

2) State what role the funders took in the study. If the funders had no role in your study, please state: "The funders had no role in study design, data collection and analysis, decision to publish, or preparation of the manuscript."

3) If any authors received a salary from any of your funders, please state which authors and which funders..

If you did not receive any funding for this study, please simply state: u201cThe authors received no specific funding for this work.u201d

**Reviewers' Comments:**

Reviewer's Responses to Questions

**Key Review Criteria Required for Acceptance?**

As you describe the new analyses required for acceptance, please consider the following:

**Methods**

-Are the objectives of the study clearly articulated with a clear testable hypothesis stated?

-Is the study design appropriate to address the stated objectives?

-Is the population clearly described and appropriate for the hypothesis being tested?

-Is the sample size sufficient to ensure adequate power to address the hypothesis being tested?

-Were correct statistical analysis used to support conclusions?

-Are there concerns about ethical or regulatory requirements being met?

Reviewer #1: See general comments

Reviewer #2: Revised

Reviewer #4: -The objectives were clearly articulated in the study

-The study design is appropriate to address the said objectives

-Yes ,the population was clearly described

_ The sample size was clearly explained and is sufficient

- (I'm not too good with statistics, so i believe the other reviewers will handle this well)

- Yes, the author DID NOT state in the methods section, if the participant CONSENTED to participate in the study, although it was mentioned in the results

**********

**Results**

-Does the analysis presented match the analysis plan?

-Are the results clearly and completely presented?

-Are the figures (Tables, Images) of sufficient quality for clarity?

Reviewer #1: See generl comments

Reviewer #2: Revised

Reviewer #4: -Yes, the analysis presented matches the analysis plan

- Yes, the results are clear and completely presented

- The figures (except for Figure 6, which looks blurred when zoomed) are all clear

**********

**Conclusions**

-Are the conclusions supported by the data presented?

-Are the limitations of analysis clearly described?

-Do the authors discuss how these data can be helpful to advance our understanding of the topic under study?

-Is public health relevance addressed?

Reviewer #1: See general comments

Reviewer #2: Revised

Reviewer #4: - Yes, the conclusions are supported by the data presented

- Yes, the limitations of the study are clearly stated

- Yes, the authors discuss the data and support it with relevant literature

**********

**Editorial and Data Presentation Modifications?**

Use this section for editorial suggestions as well as relatively minor modifications of existing data that would enhance clarity. If the only modifications needed are minor and/or editorial, you may wish to recommend “Minor Revision” or “Accept”.

Reviewer #1: See general comments

Reviewer #2: Revised

Reviewer #4: MINOR REVISIONS

- In the methods section, the authors DID NOT indicate the time period in the day the stool samples were collected as there is a recommended optimal time for stool sampling as it influences egg detection.

ABSTRACT

1. Consented should be added to the participants

2. Line 41 'is' to be replaced by 'in'

3. line 45 -49 too lengthy, consider breaking it into two sentences

4. Conclusion not too clear

INTRODUCTION

Line 96, comma (,) after Salvador

Line 118 full stop (.) after confidence

Line 167 soil transmitted helminth replaced with soil-transmitted helminths

RESULTS

Line 202, add 's' to male

Line 205 - 206 add 'of the participants'

Line 210 mention some of the similar characteristics

Line 244 add "with" after 100%

Line 298 add full stop (.) after treatment

Line 336 - 337 "in part the areas’ very vitality" This sentence is not clear

Line 348-350 the sentence not too clear, re-word it

**********

**Summary and General Comments**

Use this section to provide overall comments, discuss strengths/weaknesses of the study, novelty, significance, general execution and scholarship. You may also include additional comments for the author, including concerns about dual publication, research ethics, or publication ethics. If requesting major revision, please articulate the new experiments that are needed.

Reviewer #1: I am verysorry to say that the manuscript did not improve from the original version and actually the expanded sections produced more confusion. Many comments I made to the original manuscript in the annotated version were not addressed. I also make the autghors note that the "track changes" version of the R1 manuscript basically did not have track changes. I still cannot reconcile the methods with the results: there are many mismatches in both concepts and numbers between methods, results text and results figures/tables and some results do not even have a link to the methods. As it stand, it would be very difficult to make line-by-line comments bacuse that would involve all the manuscript. I am very sorry about this situation because I believe producing data about urban Sm infection would have been extremely useful, but the manuscript is still not usable.

Reviewer #2: Revised

Reviewer #4: This manuscript presents a long-term, longitudinal investigation of the transmission of schistosomiasis in urban areas in Salvador, Brazil. It was based on parasitological surveys conducted over 13 years involving multiple urban neighborhoods. The study addressed an important and underexplored issue: the persistence of the transmission of schistosomiasis in urban settings in spite of improvements in sanitation coverage.

Overall, the manuscript is methodologically sound, scientifically robust, and highly relevant to public health and management of neglected tropical diseases in increasingly urbanizing environments. The integration of spatial and environmental data, individual-level follow-up and longitudinal design are important strengths. With minor revisions as outlined, the manuscript is suitable for publication

**********

PLOS authors have the option to publish the peer review history of their article (what does this mean?). If published, this will include your full peer review and any attached files.

If you choose “no”, your identity will remain anonymous but your review may still be made public.

**Do you want your identity to be public for this peer review?**  For information about this choice, including consent withdrawal, please see our Privacy Policy.

Reviewer #1: No

Reviewer #2: **Yes:**  none

Reviewer #4: No

[NOTE: If reviewer comments were submitted as an attachment file, they will be attached to this email and accessible via the submission site. Please log into your account, locate the manuscript record, and check for the action link "View Attachments". If this link does not appear, there are no attachment files.]

**Figure resubmission:**

While revising your submission, we strongly recommend that you use PLOS’s NAAS tool (https://ngplosjournals.pagemajik.ai/artanalysis) to test your figure files. NAAS can convert your figure files to the TIFF file type and meet basic requirements (such as print size, resolution), or provide you with a report on issues that do not meet our requirements and that NAAS cannot fix.

After uploading your figures to PLOS’s NAAS tool - https://ngplosjournals.pagemajik.ai/artanalysis, NAAS will process the files provided and display the results in the "Uploaded Files" section of the page as the processing is complete. If the uploaded figures meet our requirements (or NAAS is able to fix the files to meet our requirements), the figure will be marked as "fixed" above. If NAAS is unable to fix the files, a red "failed" label will appear above. When NAAS has confirmed that the figure files meet our requirements, please download the file via the download option, and include these NAAS processed figure files when submitting your revised manuscript.

**Reproducibility:**

To enhance the reproducibility of your results, we recommend that authors of applicable studies deposit laboratory protocols in protocols.io, where a protocol can be assigned its own identifier (DOI) such that it can be cited independently in the future. Additionally, PLOS ONE offers an option to publish peer-reviewed clinical study protocols. Read more information on sharing protocols at https://plos.org/protocols?utm_medium=editorial-email&utm_source=authorletters&utm_campaign=protocols

# Author comment: Author response to Decision Letter 2

**DOI:** 10.1371/journal.pntd.0013418.r004

12 Feb 2026

Attachment Submitted filename: Response to reviewers vII.docx

# Peer review: Decision Letter 2

**DOI:** 10.1371/journal.pntd.0013418.r005

**Authors:** jong-Yil Chai, Feng Xue

18 Mar 2026

PNTD-D-25-01291R2Schistosomiasis as an urban disease: 13 years of studies in the neighborhoods of Salvador, BA, BrazilPLOS Neglected Tropical Diseases Dear Dr. Blanton, Thank you for submitting your manuscript to PLOS Neglected Tropical Diseases. After careful consideration, we feel that it has merit but does not fully meet PLOS Neglected Tropical Diseases's publication criteria as it currently stands. Therefore, we invite you to submit a revised version of the manuscript that addresses the points raised during the review process. Please submit your revised manuscript by Apr 17 2026 11:59PM. If you will need more time than this to complete your revisions, please reply to this message or contact the journal office at plosntds@plos.org. When you're ready to submit your revision, log on to https://www.editorialmanager.com/pntd/ and select the 'Submissions Needing Revision' folder to locate your manuscript file. Please include the following items when submitting your revised manuscript:* A letter that responds to each point raised by the editor and reviewer(s). You should upload this letter as a separate file labeled 'Response to Reviewers'. This file does not need to include responses to any formatting updates and technical items listed in the 'Journal Requirements' section below.* A marked-up copy of your manuscript that highlights changes made to the original version. You should upload this as a separate file labeled 'Revised Manuscript with Track Changes'.* An unmarked version of your revised paper without tracked changes. You should upload this as a separate file labeled 'Manuscript'.

If you would like to make changes to your financial disclosure, competing interests statement, or data availability statement, please make these updates within the submission form at the time of resubmission. Guidelines for resubmitting your figure files are available below the reviewer comments at the end of this letter.

We look forward to receiving your revised manuscript.

Kind regards, Feng Xue, Ph.D.Guest EditorPLOS Neglected Tropical Diseases Jong-Yil ChaiSection EditorPLOS Neglected Tropical Diseases

Shaden Kamhawi

co-Editor-in-Chief

PLOS Neglected Tropical Diseases

orcid.org/0000-0003-4304-636XX

Paul Brindley

co-Editor-in-Chief

PLOS Neglected Tropical Diseases

orcid.org/0000-0003-1765-0002

**********

Note: If the reviewer comments include a recommendation to cite specific previously published works, please review and evaluate these publications to determine whether they are relevant and should be cited. There is no requirement to cite these works unless the editor has indicated otherwise.

**Reviewers' comments:** Reviewer's Responses to Questions

**Key Review Criteria Required for Acceptance?**

As you describe the new analyses required for acceptance, please consider the following:

**Methods**

-Are the objectives of the study clearly articulated with a clear testable hypothesis stated?

-Is the study design appropriate to address the stated objectives?

-Is the population clearly described and appropriate for the hypothesis being tested?

-Is the sample size sufficient to ensure adequate power to address the hypothesis being tested?

-Were correct statistical analysis used to support conclusions?

-Are there concerns about ethical or regulatory requirements being met?

Reviewer #1: The major issues persisting in the manuscript regard the mismatch between methods and results and consequently conclusions. Please see the "general comments" for a point by point detail of the issues

Reviewer #4: -The objectives were clearly articulated in the study

-The study design is appropriate to address the said objectives

-Yes ,the population was clearly described

_ The sample size was clearly explained and is sufficient

- The ethical concerns have been addressed

Reviewer #5: Generally objectives are clearly stated, study design appropriate and ethical requirements have been met. However, please note the following:

"Specifically, we sought to (i) determine the

103 prealence and temporal changes in infection following treatment, (ii) identify

104 sociodemographic and environmental factors associated with S. mansoni infection; (iii)

105 distinguish locally acquired infections from potentially imported through migration or

106 travel; and (iv) explore differences between urban and traditionally rural schistosomiasis." These are the objectives. However, I fail to see clearly that iv has been addressed in the results.

**********

**Results**

-Does the analysis presented match the analysis plan?

-Are the results clearly and completely presented?

-Are the figures (Tables, Images) of sufficient quality for clarity?

Reviewer #1: The major issues persisting in the manuscript regard the mismatch between methods and results and consequently conclusions. Please see the "general comments" for a point by point detail of the issues

Reviewer #4: -Yes, the analysis presented matches the analysis plan

- Yes, the results are clear and completely presented

- The figures are all clear now

Reviewer #5: Results are OK. However, I would have liked number of individuals in figure 1 (are presented in figure 2) and number of individuals in each age group in figure 5.

**********

**Conclusions**

-Are the conclusions supported by the data presented?

-Are the limitations of analysis clearly described?

-Do the authors discuss how these data can be helpful to advance our understanding of the topic under study?

-Is public health relevance addressed?

Reviewer #1: The major issues persisting in the manuscript regard the mismatch between methods and results and consequently conclusions. Please see the "general comments" for a point by point detail of the issues

Reviewer #4: - Yes, the conclusions are supported by the data presented

- Yes, the limitations of the study are clearly stated

- Yes, the authors discuss the data and support it with relevant literature

Reviewer #5: This section is OK and the data presented are relevant in a public health context.

**********

**Editorial and Data Presentation Modifications?**

Use this section for editorial suggestions as well as relatively minor modifications of existing data that would enhance clarity. If the only modifications needed are minor and/or editorial, you may wish to recommend “Minor Revision” or “Accept”.

Reviewer #1: - Line 37: please mention Kato-Katz also in the abstract

- Line 37: what does it mean “SOME” 3964 were…”? Please rephrase by providing the actual numbers

- Line 38: what does it mean “after XXX years of infection status”? is here maybe missing something like “after XX years after PREVIOUS ASSESSMENT of infectious status”? please rephrase

- AUTHORS SUMMARY”: As per PLoS NTD requirements, the Autor Summary must be a lay version of the abstract, while this is a different comment competely, not providing data on the actual study but general considerations. It needs to be rewritten as per PLoS NTD requirements.

- Line 73-74: this sentence is not fluent. I think either the "Although" or the "but" is in excess. Please revise.

- Line 75: I suggest changing “will focus” with "will likely focus" or “should focus” or a similar toning, since the authors cannot be sure about a future trend.

- Line 77: Ref 4 is not a document focusing/demonstrating rural-to-urban migration rather that increased reproduction in cities. This concept is presented in the cited reference just in the introduction. Please change this reference with a document actually investigating or showing officially the scientific concept expressed.

- Line 91: Ref 11 is not appropriate. This is a review about morbidity factors in hotspots, and does not provide original data on morbidity figures nor comparison with malaria. Please change the reference with an appropriate one. For example, what is now ref 12 could do here.

- Line 138-139: please explain better what this contact is and how you measured this "degree".

- Line 221: “some”: please provide an actual number. Also, please explain whether for these data you relied on the interviews or if you cross-checked the real situation yourself and how you discovered and managed in the analysis of discrepancies between what said in the interview and then reality

- Line 233-234: I suggest moving this sentence upwards, after the sentence finishing with ref 28 in line 229

- Line 245: It is widely known hat lack of adequate sanitation in a house increases at community level the risk of schistosomiasis. However, why this happens might not be straightly evident to the reader. In the discussion please expand on how lack of adequate sanitation in a house increases the risk of Sm which is not directly transmissible in that households but needs contact with open water after development in a snail? And how do you explain no risk factor of not having piped water, since one might think that those people would more likely use (i.e. get into contact) contaminated water? Similarly, line 295: please explain how these interventions (paved streets, sewage system) might impact on the cycle of Sm.

- Line 259: please change “sons” to “children” (to account for M and F)

- Line 265-266 and 285: differences and changes in respect to what? another place? another time?

- Line 283-284 is indicated “only a small proportion became re-infected”. Please write actual numbers. Is indicated “Most reinfections…”, again here please provide actual numbers and data about this.

- Line 287-288: the term "round of treatment" is generally used for Mass Drug Administration (MDA) when a whole population is treated independently of individual positivity or negativity for an infection. However, from the methods I understood that treatment was at individual level, only given to eggs-positive people, not done as MDA. So please explain or change the wording. Also, by stressing that infection reduction declined “after two rounds of treatment” (as opposed to writing “along the follow-up”) makes the reader assuming that the authors explain this decline thanks to treatment- However, as it becomes more clear in the discussion, even the authors seem agreeing that treating a bunch of egg-pos people (detected by KK that has low sensitivity) cannot likely decrease transmission and other factors were more likely responsible for the change in infection figures between timepoints likely. So, please explain and amend accordingly. Finally, to grossly evaluate the impact of treatment, please provide the estimated N of people living in each neighborhood, and % coverage with questionnaires and stool exam, so that one can see if you covered (and treated) almost all people in the area or a fraction of what size.

- Discussion: rats have been identified as reservoirs of Sm maintaining the cycle even if control measures are done on the human side. Please include this aspect in the discussion

- Line 312-315: this sentence is unclear .. it seems a repetition of the sentence before

- Line 317: ref 31 is about Ethiopia, not Brazil

- Line 338-340: the sentence is unclear, please rephrase

- Line 350: please expand this concept a bit, since it is not immediately evident to the reader . Although there is ref 36, a couple of lines to provide the reader with the basics of this concept would be nice

- Line 357-358: please add references, since this very paper did not address the rural part

- Line 376-378: the sentence is unclear, please rephrase

- Line 392-397: The limitations must be placed in the discussion (not in the conclusions) and expanded

Reviewer #4: All the earlier minor errors spelt out have been addressed

Reviewer #5: Introduction: No comments

Materials/methods:

"The

120 pilot served to select study areas and inform sample size calculation, using stool

121 examinations from a convenience sample of 100 residents per site (Figure 1). A sample

122 of 72 individuals was calculated to identify an infection prevalence of 5% with 95%

123 confidence." If 100 people are sampled why do the authors use 72 individuals to calculate prevalence? This is not clear.

"A single Kato-Katz smear was prepared from

174 each sample and examined microscopically for S. mansoni" The authors should state how much fecal material was examined (50mg, 41.7mg, 25mg, other?).

**********

**Summary and General Comments**

Use this section to provide overall comments, discuss strengths/weaknesses of the study, novelty, significance, general execution and scholarship. You may also include additional comments for the author, including concerns about dual publication, research ethics, or publication ethics. If requesting major revision, please articulate the new experiments that are needed.

Reviewer #1: I revised again with interest the manuscript submitted by Santos-Muccillo and colleagues. I thank the authors for acknowledging the missed corrections after the first revision, and for having now included lines numbers, which allows a more precise feedback.

The manuscript had improved considerably for the previous versions, but there are still some issues to be solved before considering it for publication.

MAJOR ISSUES

The major issues persisting in the manuscript regard the mismatch between methods and results and consequently conclusions.

- Line 48-50: improvement in sanitation and contact with water are mentioned in the “interpretation” section of the abstract but there is no mention in the methods of such data being collected

- Line 106: the point iv) of the list of objectives is not carried out in the study, it is just narratively discussed in the discussion, but the study did not formally compare urban vs rural schistosomiasis. So please remove it from the objectives

- Please clearly write how households and individuals within households were chosen to obtain the study cohort, on the total of houses and residents living near water bodies in the buffer zone of 300-500m.

- In the methods it is indicated that 100 (pilot) and then 72 (crossectional/longitudinal study) individuals per neighborhood were needed for your aims. If the sample size was 72 per neighborhood, why were samples from >6000 people collected? This must be explained and the wording "at least" put in the sample size calculations, if you calculated the min sample size requested but you did not stick to it to stop the survey and you continued… please then write if you had a stopping criterion to recruit or what in practice was done.

- It is also unclear if the same individuals were re-examined in the longitudinal cohort. In some parts of the manuscript it seems so because the authors describe "reinfections" (= new positivity in a person who got egg-negative after previous treatment). However from table 3 one can see a "new participants" group, so it seems that you recruited different people in the follow-up. This is very unclear and must be clearly described in the methods.

- Line 260-263 it is mentioned results in the light of occupation. However, nowhere else in the methods, raw data file, and results, the analysis by occupation is carried out. Also, and likely related to this, there is no data about “contact with water”

- Line 269: this is the first time the category "migrants" are mentioned, Please explain in methods and results how this category was classified and data are presented

Reviewer #4: As stated previously, This manuscript presents a long-term, longitudinal investigation of the transmission of schistosomiasis in urban areas in Salvador, Brazil. It was based on parasitological surveys conducted over 13 years involving multiple urban neighborhoods. The study addressed an important and underexplored issue: the persistence of the transmission of schistosomiasis in urban settings in spite of improvements in sanitation coverage.

Overall, the manuscript is methodologically sound, scientifically robust, and highly relevant to public health and management of neglected tropical diseases in increasingly urbanizing environments. The integration of spatial and environmental data, individual-level follow-up and longitudinal design are important strengths. Since all the minor errors have been addressed, the manuscript is suitable for publication

Reviewer #5: I recommend publication with minor changes.

**********

PLOS authors have the option to publish the peer review history of their article (what does this mean?). If published, this will include your full peer review and any attached files.

If you choose “no”, your identity will remain anonymous but your review may still be made public.

**Do you want your identity to be public for this peer review?**  For information about this choice, including consent withdrawal, please see our Privacy Policy.

Reviewer #1: No

Reviewer #4: No

Reviewer #5: No

[NOTE: If reviewer comments were submitted as an attachment file, they will be attached to this email and accessible via the submission site. Please log into your account, locate the manuscript record, and check for the action link "View Attachments". If this link does not appear, there are no attachment files.]

**Figure resubmission:**

While revising your submission, we strongly recommend that you use PLOS’s NAAS tool (https://ngplosjournals.pagemajik.ai/artanalysis) to test your figure files. NAAS can convert your figure files to the TIFF file type and meet basic requirements (such as print size, resolution), or provide you with a report on issues that do not meet our requirements and that NAAS cannot fix.

After uploading your figures to PLOS’s NAAS tool - https://ngplosjournals.pagemajik.ai/artanalysis, NAAS will process the files provided and display the results in the "Uploaded Files" section of the page as the processing is complete. If the uploaded figures meet our requirements (or NAAS is able to fix the files to meet our requirements), the figure will be marked as "fixed" above. If NAAS is unable to fix the files, a red "failed" label will appear above. When NAAS has confirmed that the figure files meet our requirements, please download the file via the download option, and include these NAAS processed figure files when submitting your revised manuscript. **Reproducibility:**  To enhance the reproducibility of your results, we recommend that authors of applicable studies deposit laboratory protocols in protocols.io, where a protocol can be assigned its own identifier (DOI) such that it can be cited independently in the future. Additionally, PLOS ONE offers an option to publish peer-reviewed clinical study protocols. Read more information on sharing protocols at https://plos.org/protocols?utm_medium=editorial-email&utm_source=authorletters&utm_campaign=protocols

# Author comment: Author response to Decision Letter 3

**DOI:** 10.1371/journal.pntd.0013418.r006

26 Apr 2026

Attachment Submitted filename: Authors Responses.docx

# Peer review: Decision Letter 3

**DOI:** 10.1371/journal.pntd.0013418.r007

**Authors:** jong-Yil Chai, Feng Xue

8 Jun 2026

PNTD-D-25-01291R3Schistosomiasis as an urban disease: 13 years of studies in the neighborhoods of Salvador, BA, BrazilPLOS Neglected Tropical Diseases Dear Dr. Blanton, Thank you for submitting your manuscript to PLOS Neglected Tropical Diseases. After careful consideration, we feel that it has merit but does not fully meet PLOS Neglected Tropical Diseases's publication criteria as it currently stands. Therefore, we invite you to submit a revised version of the manuscript that addresses the points raised during the review process. Please submit your revised manuscript by Jul 08 2026 11:59PM. If you will need more time than this to complete your revisions, please reply to this message or contact the journal office at plosntds@plos.org. When you're ready to submit your revision, log on to https://www.editorialmanager.com/pntd/ and select the 'Submissions Needing Revision' folder to locate your manuscript file. Please include the following items when submitting your revised manuscript:* A letter that responds to each point raised by the editor and reviewer(s). You should upload this letter as a separate file labeled 'Response to Reviewers'. This file does not need to include responses to any formatting updates and technical items listed in the 'Journal Requirements' section below.* A marked-up copy of your manuscript that highlights changes made to the original version. You should upload this as a separate file labeled 'Revised Manuscript with Track Changes'.* An unmarked version of your revised paper without tracked changes. You should upload this as a separate file labeled 'Manuscript'. If you would like to make changes to your financial disclosure, competing interests statement, or data availability statement, please make these updates within the submission form at the time of resubmission. Guidelines for resubmitting your figure files are available below the reviewer comments at the end of this letter. As the corresponding author, your ORCID iD is verified in the submission system and will appear in the published article. PLOS supports the use of ORCID, and we encourage all coauthors to register for an ORCID iD and use it as well. Please encourage your coauthors to verify their ORCID iD within the submission system before final acceptance, as unverified ORCID iDs will not appear in the published article. Only the individual author can complete the verification step; PLOS staff cannot verify ORCID iDs on behalf of authors.We look forward to receiving your revised manuscript. Kind regards, Feng Xue, Ph.D.Guest EditorPLOS Neglected Tropical Diseases Jong-Yil ChaiSection EditorPLOS Neglected Tropical Diseases

Shaden Kamhawi

co-Editor-in-Chief

PLOS Neglected Tropical Diseases

orcid.org/0000-0003-4304-636XX

Paul Brindley

co-Editor-in-Chief

PLOS Neglected Tropical Diseases

orcid.org/0000-0003-1765-0002

**Additional Editor Comments (if provided):**

**Reviewers' comments:**

Reviewer's Responses to Questions

**Key Review Criteria Required for Acceptance?**

As you describe the new analyses required for acceptance, please consider the following:

**Methods**

-Are the objectives of the study clearly articulated with a clear testable hypothesis stated?

-Is the study design appropriate to address the stated objectives?

-Is the population clearly described and appropriate for the hypothesis being tested?

-Is the sample size sufficient to ensure adequate power to address the hypothesis being tested?

-Were correct statistical analysis used to support conclusions?

-Are there concerns about ethical or regulatory requirements being met?

Reviewer #1: See general comments

Reviewer #6: (No Response)

Reviewer #7: See general comments

Reviewer #8: The objectives of the study are clearly articulated, and the manuscript presents a clear and testable hypothesis. The study design is appropriate for addressing the stated objectives, and the population is adequately described and suitable for the hypothesis being tested. The sample size appears sufficient to provide adequate power for the analyses and to support the study conclusions. The statistical methods used are generally appropriate for the study design and are sufficient to support the conclusions drawn from the data. However, as noted above, the authors should consider reporting age as median with interquartile range rather than mean with standard deviation, particularly because the standard deviation is large, suggesting skewness. I do not have major concerns regarding ethical or regulatory requirements, as these appear to have been adequately addressed in the manuscript.

**********

**Results**

-Does the analysis presented match the analysis plan?

-Are the results clearly and completely presented?

-Are the figures (Tables, Images) of sufficient quality for clarity?

Reviewer #1: See general comments

Reviewer #6: (No Response)

Reviewer #7: See general comments

Reviewer #8: The analysis presented appears to be consistent with the stated analysis plan. The results are generally clearly and completely presented, although some areas would benefit from improved wording and more precise reporting, as noted below. The tables and figures are generally of sufficient quality and contribute to the clarity of the manuscript. However, the presentation of tabulated information within the Figure 2 legend may be awkward in the final published version, and I recommend converting this information into a supplementary table and referring to it appropriately in the legend.

**********

**Conclusions**

-Are the conclusions supported by the data presented?

-Are the limitations of analysis clearly described?

-Do the authors discuss how these data can be helpful to advance our understanding of the topic under study?

-Is public health relevance addressed?

Reviewer #1: See general comments

Reviewer #6: (No Response)

Reviewer #7: See general comments

Reviewer #8: The conclusions are generally supported by the data presented. The authors also describe the limitations of the study. The manuscript discusses how the data contribute to understanding the topic under study. The public health relevance is also adequately addressed, as the findings have implications for surveillance, treatment strategies, and schistosomiasis control in rapidly changing or urbanising settings.

**********

**Editorial and Data Presentation Modifications?**

Use this section for editorial suggestions as well as relatively minor modifications of existing data that would enhance clarity. If the only modifications needed are minor and/or editorial, you may wish to recommend “Minor Revision” or “Accept”.

Reviewer #1: None of my previous observations was addressded

Reviewer #6: (No Response)

Reviewer #7: (No Response)

Reviewer #8: Comment 1: Line 227: It would be more informative to report the median age, preferably with the interquartile range, rather than the average age with standard deviation, especially due to the large SD.

Comment 2: Figure 2 legend: Including a table within a figure legend may be awkward in the final printed version. The table should be converted into a supplementary table and referred to appropriately within the Figure 2 legend.

Comment 3: The manuscript would benefit from careful English-language revision to improve clarity, specificity, and syntax. Several sentences are ambiguous, imprecisely worded, or syntactically awkward. The examples below represent only a small selection of the language and clarity issues observed; similar problems occur throughout the manuscript and should be addressed comprehensively.

Specific comments

1. Line 93: Schistosoma haematobium should be written in full at first mention before using the abbreviated form S. haematobium.

2. Line 37: The sentence is imprecise. If the exact number of participants tested was 3,964, the word “some” should be deleted. The sentence could be revised as follows: “Of the participants, 3,964 were tested, treated, and re-examined up to three times at varying intervals within 1–5 years of positive diagnosis.”

5. Lines 62–64: The sentence beginning “Urban areas also offer opportunities…” is unclear. It is not evident what “opportunities” refers to in the context of the preceding statements. The authors should revise this sentence to specify the nature of these opportunities.

6. Lines 66–67: The meaning of the phrase “Brazil may preview what to expect in other rapidly urbanizing areas of the world” is unclear. This statement should be rephrased to clarify whether the authors mean that the Brazilian setting provides a model for understanding schistosomiasis transmission in other rapidly urbanising regions.

7. Lines 90–91: The sentence would be clearer if revised as follows: “Schistosomiasis is considered the second most important parasitic disease of public health significance after malaria.”

**********

**Summary and General Comments**

Use this section to provide overall comments, discuss strengths/weaknesses of the study, novelty, significance, general execution and scholarship. You may also include additional comments for the author, including concerns about dual publication, research ethics, or publication ethics. If requesting major revision, please articulate the new experiments that are needed.

Reviewer #1: I need to not that the authors did not consider important to thoroughly address in both text and reply the observations made to R2 version. I perceive this as a complete lack of respect for the time spent on the paper and the attention given to the topic. I will be heppy to revise a version that throughly adress ALL the observations.

Reviewer #6: This is a well-executed study that demonstrates findings of considerable public health significance regarding the epidemiology of intestinal Schistosomiasis (S. mansoni) in an urban setting of Brazil. The methods and statistical approaches are appropriate to the objective of the study, which are fairly modest and descriptive in scope. The conclusions are fairly well supported by the results, and the discussion situates these findings within the wider body of evidence successfully, while acknowledging some limitations. The finding that intestinal Schistosomiasis exhibits a high prevalence in adult men engaged in certain occupations, is an important one and will be of interest to the journal's readership. This manuscript has already undergone several rounds of review, and I see no reason to delay it's publication any further. I consider it to be worthy of publication in PLOS NTDs in its current form.

Reviewer #7: The authors present informative data on the changes in S. mansoni prevalence in the urban environments of Salvador. This presents considerable work over a very long time period. The authors have greatly improved the paper over several iterations. The main recommendation now is to make it very clear what data has come from previous publications and make this clear throughout the introduction, methods and results. Specific comments are below.

Line 99 – 106 – there is no mention of the single cross-sectional surveys. Also explain here that some of the data is from other published work. Make it also clear that the baseline and follow-ups are not uniform across all neighbourhoods.

Line 117 – make it clear when the pilot was done

Line 118 – be more detailed in what you mean by “snails shedding cercariae” and “recorded schistosomiasis”

Line 122- How was the sample size calculated

Figure 1 suggests that all selected neighbourhoods had a>5% prevalence, but IN the text it says 3 had <5%?

Line 120 – make it clear what stool examination was done.

Make it clear in the text that the timing of the surveys was not uniform across the neighbourhoods, and it is not clear if the pilot was at a different time in all neighbourhoods or not, especially as baseline prevalences shown in Figure 4 are from different years.

Line 140 – when writing, the authors need to make it very clear when data is not from this study, e.g. they should say “Previous studies had shown ……”

Also, in the figures and text, it needs to be very clear what survey has been published previously, and the authors need to have that as a separate section in the methods, as it comes in as just data analysis rather than data collection. This also needs to be very clear in the presentation of the results. If data is presented from previous publications, the reference must be added.

In Figure 3 – split the 3 single surveys so they are presented longitudinally so it is clear there are 3.

In the discussion, discuss morbidity in terms of whether low levels of infection/transmission are imported to monitor and treat or not.

Reviewer #8: Overall, the scientific methodology is sound but the manuscript requires comprehensive language editing to ensure that the arguments are clearly presented and that technical statements are precise, specific, and suitable for a scientific audience.

**********

PLOS authors have the option to publish the peer review history of their article (what does this mean?). If published, this will include your full peer review and any attached files.

If you choose “no”, your identity will remain anonymous but your review may still be made public.

**Do you want your identity to be public for this peer review?**  For information about this choice, including consent withdrawal, please see our Privacy Policy.

Reviewer #1: No

Reviewer #6: **Yes:**  Josh M Colston

Reviewer #7: No

Reviewer #8: No

[NOTE: If reviewer comments were submitted as an attachment file, they will be attached to this email and accessible via the submission site. Please log into your account, locate the manuscript record, and check for the action link "View Attachments". If this link does not appear, there are no attachment files.] **Figure resubmission:**   While revising your submission, we strongly recommend that you use PLOS’s NAAS tool (https://ngplosjournals.pagemajik.ai/artanalysis) to test your figure files. NAAS can convert your figure files to the TIFF file type and meet basic requirements (such as print size, resolution), or provide you with a report on issues that do not meet our requirements and that NAAS cannot fix.

After uploading your figures to PLOS’s NAAS tool - https://ngplosjournals.pagemajik.ai/artanalysis, NAAS will process the files provided and display the results in the "Uploaded Files" section of the page as the processing is complete. If the uploaded figures meet our requirements (or NAAS is able to fix the files to meet our requirements), the figure will be marked as "fixed" above. If NAAS is unable to fix the files, a red "failed" label will appear above. When NAAS has confirmed that the figure files meet our requirements, please download the file via the download option, and include these NAAS processed figure files when submitting your revised manuscript. **Reproducibility:**  To enhance the reproducibility of your results, we recommend that authors of applicable studies deposit laboratory protocols in protocols.io, where a protocol can be assigned its own identifier (DOI) such that it can be cited independently in the future. Additionally, PLOS ONE offers an option to publish peer-reviewed clinical study protocols. Read more information on sharing protocols at https://plos.org/protocols?utm_medium=editorial-email&utm_source=authorletters&utm_campaign=protocols

# Author comment: Author response to Decision Letter 4

**DOI:** 10.1371/journal.pntd.0013418.r008

5 Aug 2026

Attachment Submitted filename: Response to Reviewers.docx

# Peer review: Decision Letter 4

**DOI:** 10.1371/journal.pntd.0013418.r009

**Authors:** jong-Yil Chai, Feng Xue

25 Aug 2026

PNTD-D-25-01291R4Schistosomiasis as an urban disease: 13 years of studies in the neighborhoods of Salvador, BA, BrazilPLOS Neglected Tropical DiseasesDear Dr. Blanton,Thank you for submitting your manuscript to PLOS Neglected Tropical Diseases. After careful consideration, we feel that it has merit but does not fully meet PLOS Neglected Tropical Diseases's publication criteria as it currently stands. Therefore, we invite you to submit a revised version of the manuscript that addresses the points raised during the review process.Please submit your revised manuscript by Sep 24 2026 11:59PM. If you will need more time than this to complete your revisions, please reply to this message or contact the journal office at plosntds@plos.org. When you're ready to submit your revision, log on to https://www.editorialmanager.com/pntd/ and select the 'Submissions Needing Revision' folder to locate your manuscript file.Please include the following items when submitting your revised manuscript:* A letter that responds to each point raised by the editor and reviewer(s). You should upload this letter as a separate file labeled 'Response to Reviewers'. This file does not need to include responses to any formatting updates and technical items listed in the 'Journal Requirements' section below.* A marked-up copy of your manuscript that highlights changes made to the original version. You should upload this as a separate file labeled 'Revised Manuscript with Track Changes'.* An unmarked version of your revised paper without tracked changes. You should upload this as a separate file labeled 'Manuscript'.If you would like to make changes to your financial disclosure, competing interests statement, or data availability statement, please make these updates within the submission form at the time of resubmission. Guidelines for resubmitting your figure files are available below the reviewer comments at the end of this letter.As the corresponding author, your ORCID iD is verified in the submission system and will appear in the published article. PLOS supports the use of ORCID, and we encourage all coauthors to register for an ORCID iD and use it as well. Please encourage your coauthors to verify their ORCID iD within the submission system before final acceptance, as unverified ORCID iDs will not appear in the published article. Only the individual author can complete the verification step; PLOS staff cannot verify ORCID iDs on behalf of authors.We look forward to receiving your revised manuscript.Kind regards,Feng Xue, Ph.D.Guest EditorPLOS Neglected Tropical DiseasesJong-Yil ChaiSection EditorPLOS Neglected Tropical Diseases

Shaden Kamhawi

co-Editor-in-Chief

PLOS Neglected Tropical Diseases

orcid.org/0000-0003-4304-636XX

Paul Brindley

co-Editor-in-Chief

PLOS Neglected Tropical Diseases

orcid.org/0000-0003-1765-0002

**Reviewers' comments:**

Reviewer's Responses to Questions

**Key Review Criteria Required for Acceptance?**

As you describe the new analyses required for acceptance, please consider the following:

**Methods**

-Are the objectives of the study clearly articulated with a clear testable hypothesis stated?

-Is the study design appropriate to address the stated objectives?

-Is the population clearly described and appropriate for the hypothesis being tested?

-Is the sample size sufficient to ensure adequate power to address the hypothesis being tested?

-Were correct statistical analysis used to support conclusions?

-Are there concerns about ethical or regulatory requirements being met?

Reviewer #6: (No Response)

Reviewer #7: The methods are much improved but still need further detail (see comments on the track change document).

Reviewer #8: (No Response)

**********

**Results**

-Does the analysis presented match the analysis plan?

-Are the results clearly and completely presented?

-Are the figures (Tables, Images) of sufficient quality for clarity?

Reviewer #6: (No Response)

Reviewer #7: The results are much improved but still need further detail and clarification of data from past publications (see comments on the track change document).

Reviewer #8: I thank the authors for the clarification. I agree that consistency of presentation can be desirable; however, the choice of descriptive statistics should primarily be determined by the distribution of the data rather than by visual standardization. Please confirm whether the variables presented as mean ± SD were approximately normally distributed and indicate how distributional assumptions were assessed. If any variables show substantial skewness or important outliers, they should be reported as median (IQR), irrespective of the summary statistic used for the other variables.

**********

**Conclusions**

-Are the conclusions supported by the data presented?

-Are the limitations of analysis clearly described?

-Do the authors discuss how these data can be helpful to advance our understanding of the topic under study?

-Is public health relevance addressed?

Reviewer #6: (No Response)

Reviewer #7: The methods are much improved some comments are detailed in the track change document.

Reviewer #8: (No Response)

**********

**Editorial and Data Presentation Modifications?**

Use this section for editorial suggestions as well as relatively minor modifications of existing data that would enhance clarity. If the only modifications needed are minor and/or editorial, you may wish to recommend “Minor Revision” or “Accept”.

Reviewer #6: (No Response)

Reviewer #7: (No Response)

Reviewer #8: (No Response)

**********

**Summary and General Comments**

Use this section to provide overall comments, discuss strengths/weaknesses of the study, novelty, significance, general execution and scholarship. You may also include additional comments for the author, including concerns about dual publication, research ethics, or publication ethics. If requesting major revision, please articulate the new experiments that are needed.

Reviewer #6: I previously recommended that this manuscript be accepted for publication, and I am still of this opinion now that it has undergone a further round of minor revisions and some editorial concerns have been addressed. Congratulations to the authors on a noteworthy contribution to the subject area.

Reviewer #7: The paper is much improved. However, the rebuttal was very confusing, so I went through the track-changes document in detail and added comments. Overall clarity and details needed input. The authors also need to find a way to make it very clear which data are from the previous publications. The authors should also not try to merge lots of past publications of their own with this one. I hope the detailed comments in the attachment will help, as I feel the data and findings are valuable. No supplementary tables were submitted.

Reviewer #8: (No Response)

**********

PLOS authors have the option to publish the peer review history of their article (what does this mean?). If published, this will include your full peer review and any attached files.

If you choose “no”, your identity will remain anonymous but your review may still be made public.

**Do you want your identity to be public for this peer review?**  For information about this choice, including consent withdrawal, please see our Privacy Policy.

Reviewer #6: **Yes:**  Josh M. Colston

Reviewer #7: No

Reviewer #8: No

[NOTE: If reviewer comments were submitted as an attachment file, they will be attached to this email and accessible via the submission site. Please log into your account, locate the manuscript record, and check for the action link "View Attachments". If this link does not appear, there are no attachment files.] **Figure resubmission:**   While revising your submission, we strongly recommend that you use PLOS’s NAAS tool (https://ngplosjournals.pagemajik.ai/artanalysis) to test your figure files. NAAS can convert your figure files to the TIFF file type and meet basic requirements (such as print size, resolution), or provide you with a report on issues that do not meet our requirements and that NAAS cannot fix.

After uploading your figures to PLOS’s NAAS tool - https://ngplosjournals.pagemajik.ai/artanalysis, NAAS will process the files provided and display the results in the "Uploaded Files" section of the page as the processing is complete. If the uploaded figures meet our requirements (or NAAS is able to fix the files to meet our requirements), the figure will be marked as "fixed" above. If NAAS is unable to fix the files, a red "failed" label will appear above. When NAAS has confirmed that the figure files meet our requirements, please download the file via the download option, and include these NAAS processed figure files when submitting your revised manuscript. **Reproducibility:**  To enhance the reproducibility of your results, we recommend that authors of applicable studies deposit laboratory protocols in protocols.io, where a protocol can be assigned its own identifier (DOI) such that it can be cited independently in the future. Additionally, PLOS ONE offers an option to publish peer-reviewed clinical study protocols. Read more information on sharing protocols at https://plos.org/protocols?utm_medium=editorial-email&utm_source=authorletters&utm_campaign=protocols

Attachment Submitted filename: Urban Schisto PLoS NTD_v.9 Tracked.docx

# Author comment: Author response to Decision Letter 5

**DOI:** 10.1371/journal.pntd.0013418.r010

27 Sep 2026

Attachment Submitted filename: Response_to_Reviewers_auresp_5.docx

# Peer review: Decision Letter 5

**DOI:** 10.1371/journal.pntd.0013418.r011

**Authors:** jong-Yil Chai, Feng Xue

5 Oct 2026

Dear Dr. Blanton,

We are pleased to inform you that your manuscript 'Schistosomiasis as an urban disease: 13 years of studies in the neighborhoods of Salvador, BA, Brazil' has been provisionally accepted for publication in PLOS Neglected Tropical Diseases.

Before your manuscript can be formally accepted you will need to complete some formatting changes, which you will receive in a follow up email. A member of our team will be in touch with a set of requests.

Please note that your manuscript will not be scheduled for publication until you have made the required changes, so a swift response is appreciated.

IMPORTANT: The editorial review process is now complete. PLOS will only permit corrections to spelling, formatting or significant scientific errors from this point onwards. Requests for major changes, or any which affect the scientific understanding of your work, will cause delays to the publication date of your manuscript.

Should you, your institution's press office or the journal office choose to press release your paper, you will automatically be opted out of early publication. We ask that you notify us now if you or your institution is planning to press release the article. All press must be co-ordinated with PLOS.

Thank you again for supporting Open Access publishing; we are looking forward to publishing your work in PLOS Neglected Tropical Diseases.

Best regards,

Feng Xue, Ph.D.

Guest Editor

PLOS Neglected Tropical Diseases

Jong-Yil Chai

Section Editor

PLOS Neglected Tropical Diseases

Shaden Kamhawi

co-Editor-in-Chief

PLOS Neglected Tropical Diseases

orcid.org/0000-0003-4304-636XX

Paul Brindley

co-Editor-in-Chief

PLOS Neglected Tropical Diseases

orcid.org/0000-0003-1765-0002

***********************************************************

Reviewer's Responses to Questions

**Key Review Criteria Required for Acceptance?**

As you describe the new analyses required for acceptance, please consider the following:

**Methods**

-Are the objectives of the study clearly articulated with a clear testable hypothesis stated?

-Is the study design appropriate to address the stated objectives?

-Is the population clearly described and appropriate for the hypothesis being tested?

-Is the sample size sufficient to ensure adequate power to address the hypothesis being tested?

-Were correct statistical analysis used to support conclusions?

-Are there concerns about ethical or regulatory requirements being met?

Reviewer #6: (No Response)

Reviewer #8: (No Response)

**********

**Results**

-Does the analysis presented match the analysis plan?

-Are the results clearly and completely presented?

-Are the figures (Tables, Images) of sufficient quality for clarity?

Reviewer #6: (No Response)

Reviewer #8: (No Response)

**********

**Conclusions**

-Are the conclusions supported by the data presented?

-Are the limitations of analysis clearly described?

-Do the authors discuss how these data can be helpful to advance our understanding of the topic under study?

-Is public health relevance addressed?

Reviewer #6: (No Response)

Reviewer #8: (No Response)

**********

**Editorial and Data Presentation Modifications?**

Use this section for editorial suggestions as well as relatively minor modifications of existing data that would enhance clarity. If the only modifications needed are minor and/or editorial, you may wish to recommend “Minor Revision” or “Accept”.

Reviewer #6: (No Response)

Reviewer #8: (No Response)

**********

**Summary and General Comments**

Use this section to provide overall comments, discuss strengths/weaknesses of the study, novelty, significance, general execution and scholarship. You may also include additional comments for the author, including concerns about dual publication, research ethics, or publication ethics. If requesting major revision, please articulate the new experiments that are needed.

Reviewer #6: As was my assessment in the preceding two rounds of revisions, this manuscript can be accepted for publication in PLOS NTDs.

Reviewer #8: The authors have an unusual way of responding to comments and this has been consistently noted by several of the reviewers who reviewed this manuscript. Ideally every single comment at every round need a specific response. My comment in this round was simply ignored. I do not have a recommendation for this round.

**********

PLOS authors have the option to publish the peer review history of their article (what does this mean?). If published, this will include your full peer review and any attached files.

If you choose “no”, your identity will remain anonymous but your review may still be made public.

**Do you want your identity to be public for this peer review?**  For information about this choice, including consent withdrawal, please see our Privacy Policy.

Reviewer #6: No

Reviewer #8: No

# Editor report: Acceptance letter

**DOI:** 10.1371/journal.pntd.0013418.r012

**Authors:** jong-Yil Chai, Feng Xue

Dear Dr. Blanton,

We are delighted to inform you that your manuscript, "Schistosomiasis as an urban disease: 13 years of studies in the neighborhoods of Salvador, BA, Brazil," has been formally accepted for publication in PLOS Neglected Tropical Diseases.

We have now passed your article onto the PLOS Production Department who will complete the rest of the publication process. All authors will receive a confirmation email upon publication.

The corresponding author will soon be receiving a typeset proof for review, to ensure errors have not been introduced during production. Please review the PDF proof of your manuscript carefully, as this is the last chance to correct any scientific or type-setting errors. Please note that major changes, or those which affect the scientific understanding of the work, will likely cause delays to the publication date of your manuscript. Note: Proofs for Front Matter articles (Editorial, Viewpoint, Symposium, Review, etc...) are generated on a different schedule and may not be made available as quickly.

Soon after your final files are uploaded, the early version of your manuscript will be published online unless you opted out of this process. The date of the early version will be your article's publication date. The final article will be published to the same URL, and all versions of the paper will be accessible to readers.

For Research Articles, you will receive an invoice from PLOS for your publication fee after your manuscript has reached the completed accept phase. If you receive an email requesting payment before acceptance or for any other service, this may be a phishing scheme. Learn how to identify phishing emails and protect your accounts at https://explore.plos.org/phishing.

Thank you again for supporting open-access publishing; we are looking forward to publishing your work in PLOS Neglected Tropical Diseases.

Best regards,

Shaden Kamhawi

co-Editor-in-Chief

PLOS Neglected Tropical Diseases

Paul Brindley

co-Editor-in-Chief

PLOS Neglected Tropical Diseases
